# Mn(III)-Catalyzed Synthesis of Selenophosphates and Tellurophosphates

**DOI:** 10.3390/molecules31122103

**Published:** 2026-06-15

**Authors:** Jialu Wang, Jun Liu, Yuhui Xie, Changjiang Wu, Xinyu Wang, Gong-Qing Liu

**Affiliations:** Organoselenium Synthesis and Function Laboratory, School of Pharmacy, Nantong University, Nantong 226019, China; jialuwang191@163.com (J.W.); xyh031016@163.com (Y.X.); hyzc357@163.com (C.W.); wangxy@163.com (X.W.)

**Keywords:** manganese(III) acetate, selenophosphates, tellurophosphates, dichalcogenides, phosphites

## Abstract

Selenophosphates and tellurophosphates are important classes of organophosphorus compounds that exhibit unique reactivity, tunable redox properties, and significant biological activity. Herein, we report a mild and efficient Mn(III)-catalyzed method for synthesizing selenophosphates and tellurophosphates. Using Mn(OAc)_3_ as the catalyst and tetrahydrofuran (THF) as the solvent, various H-phosphonates and H-phosphine oxides undergo radical coupling with diselenides or ditellurides at room temperature in the presence of air, affording good-to-excellent yields of the corresponding chalcogenophosphates. The protocol features a broad substrate scope, favorable functional group tolerance, and low catalyst loading while avoiding the use of toxic reagents or strong oxidants. Mechanistic studies suggest that the reaction proceeds via the phosphinyl radicals generated through single-electron oxidation mediated by Mn(III).

## 1. Introduction

**Figure 1:**
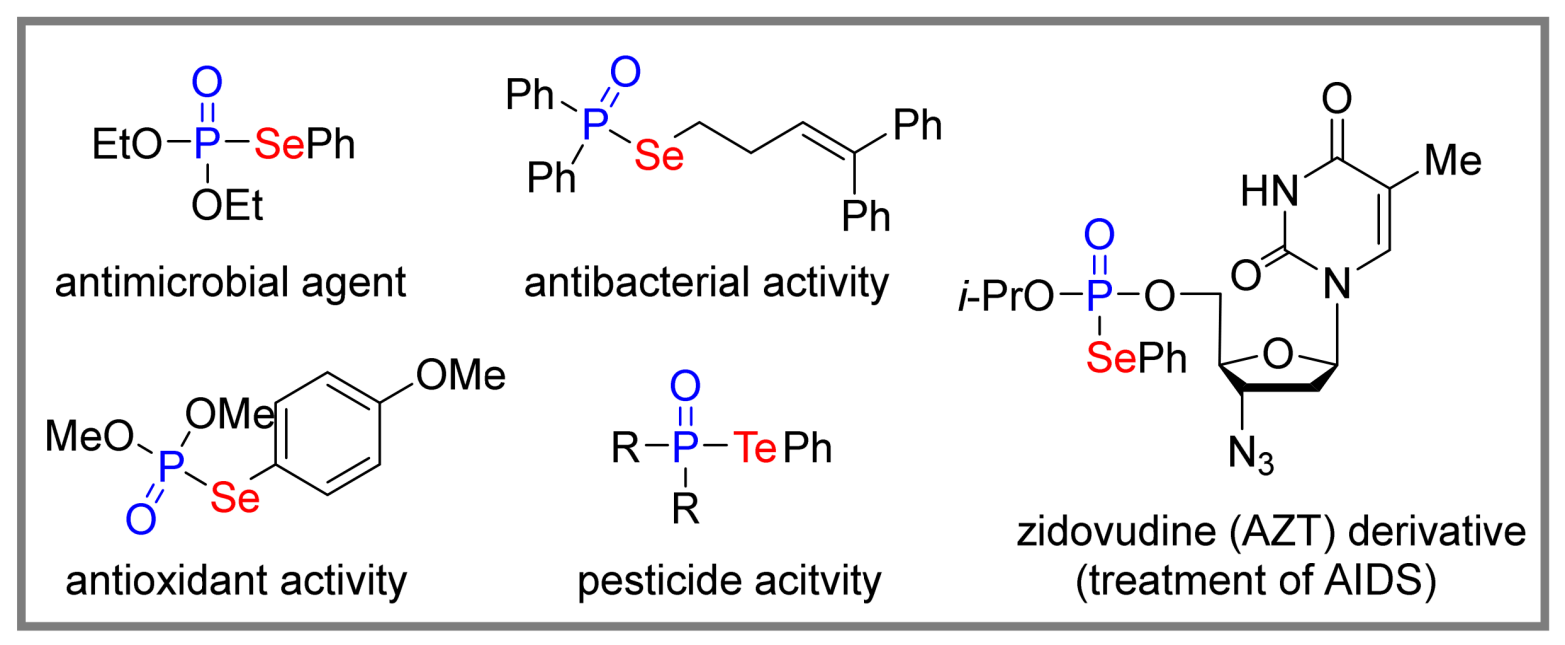
Representative biologically active selenophosphates and tellurophosphates.

Organophosphates are fundamental to both chemistry and life, serving as indispensable structural motifs in pharmaceuticals, agrochemicals, materials science, and organic synthesis. Their significance is most clearly exemplified by their role as the backbone of DNA and RNA [1,2,3]. In this context, selenophosphates and tellurophosphates have attracted considerable attention. Compared with their oxygen analogs, these heavier chalcogenophosphates often exhibit unique reactivity, tunable redox behavior, and distinctive spectroscopic features (e.g., ^77^Se NMR), making them valuable in medicinal chemistry, agrochemistry, and biopharmaceutical research [4,5,6,7]. As illustrated in Figure 1, representative selenophosphates and tellurophosphates display a range of biological activities, including antimicrobial, antioxidant, and pesticidal effects, as well as potential applications in AIDS treatment.

**Scheme 1:**
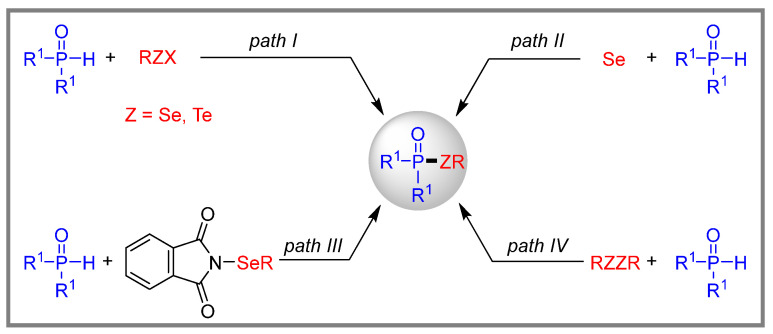
Synthetic strategies for chalcogenophosphates.

Consequently, substantial efforts have been devoted to developing efficient synthetic strategies for these compounds. Traditional methods for preparing chalcogenophosphates typically rely on activated chalcogenation reagents such as RZX (X = H, Br, Cl, etc.) (Scheme 1, path I) [8,9]. However, their practical use is substantially limited by issues such as unpleasant odor and sensitivity to oxygen and moisture. Elemental selenium and tellurium are considered ideal candidates for synthesizing chalcogenophosphates because of their nontoxic nature and high chemical stability (Scheme 1, path II) [10,11,12]. However, to date, direct activation of elemental tellurium with phosphorus sources for tellurophosphate synthesis has not been reported. Additionally, *N*-selenophthalimide has also been used as an excellent selenium source for the catalyst-free synthesis of selenophosphates (Scheme 1, path III), [13,14], but its high cost and limited structural diversity restrict broader application. By contrast, dichalcogenides are highly stable and easy-to-handle chalcogenation reagents and have therefore become preferred substrates. Various strategies for synthesizing chalcogenophosphates from dichalcogenides and phosphites including transition metal catalysis, [15,16,17]; base-promoted reactions, [18,19]; oxidant-mediated processes, [20,21,22]; radical initiation, [23,24]; photoredox catalysis, [25,26]; and electrochemical methods [27,28] (Scheme 1, path IV) have been developed.

**Scheme 2:**
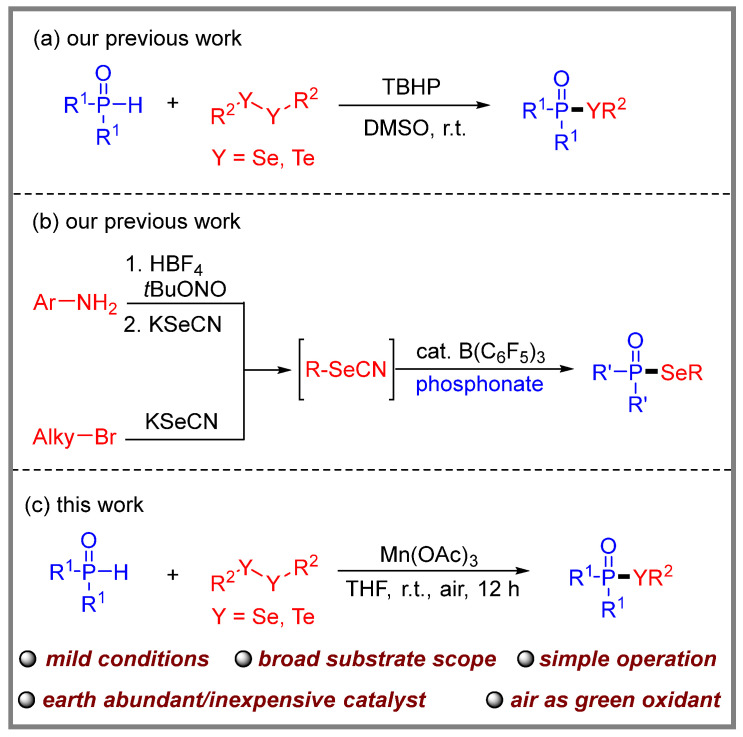
Previous and present work on the synthesis of selenophosphates and tellurophosphates.

Recently, we developed several general and mild methods for synthesizing chalcogen-containing compounds using hypervalent iodine, [29] N–F reagents, [30] photochemical strategies [31,32], and borane catalysts [33]. As part of this effort, we reported a *tert*-butyl hydroperoxide (TBHP)-mediated oxidative coupling of phosphites with diselenides or ditellurides to synthesize phosphoroselenoates and phosphorotelluroates (Scheme 2a) [34]. However, this method requires 2.5 equivalents of TBHP, raising concerns regarding scalability and safety; moreover, the use of the high-boiling-point solvent dimethyl sulfoxide (DMSO) complicates product isolation on a larger scale. More recently, we developed a protocol for synthesizing selenophosphates, wherein selenocyanates generated in situ from anilines or alkyl bromides are coupled with phosphites under B(C_6_F_5_)_3_ catalysis to form the P–Se bond (Scheme 2b) [33]. Nevertheless, this approach generates hydrogen cyanide (HCN), a highly toxic and volatile inorganic compound that poses significant risks to both human health and the environment, even at low concentrations.

Manganese has attracted increasing attention in catalysis because of its natural abundance, low cost, and low toxicity [35,36]. In addition, manganese exhibits multiple stable oxidation states, endowing it with versatile redox reactivity and the ability to participate in electron transfer-mediated redox transformations. In particular, Mn(III) catalysis facilitates the formation of diverse chemical bonds through single-electron oxidation pathways [37,38,39]. For example, Wang et al. reported a Mn(OAc)_3_-promoted cross-coupling reaction of benzothiazole/thiazole derivatives with organophosphorus compounds [40]. In 2020, Li, Song and co-workers developed manganese(III)-promoted tandem phosphinoylation/cyclization of 2-arylindoles/2-arylbenzimidazoles with disubstituted phosphine oxides [41]. Very recently, Sun et al. reported a Mn(OAc)_3_-mediated radical cascade cyclization of *N*-propargyl enamides with various H-phosphine oxides, H-phosphinates and H-phosphonates, enabling the synthesis of multi-substituted 3-phosphorylpyridines [42]. In these cases, the single-electron oxidation of phosphonates by Mn(OAc)_3_ generates a phosphinyl radical, which subsequently triggers further transformation.

Although Mn(III)-catalyzed reactions have been widely applied in C–P bond formation, [43], the formation of Se–P and Te–P bonds via Mn(III) catalysis remains underexplored. Given the high electron density at the phosphorus center, phosphorus-centered radicals can be readily generated through Mn(III)-mediated single-electron oxidation. We hypothesized that these phosphinyl radicals could be intercepted by diselenides and ditellurides to form Se–P and Te–P bonds efficiently, thereby enabling selenophosphate and tellurophosphate synthesis.

Herein, we report a Mn(III)-catalyzed method for synthesizing selenophosphates and tellurophosphates from H-phosphonates or H-phosphine oxides with dichalcogenides (diselenides and ditellurides) (Scheme 2c) as part of our ongoing studies on chalcogen chemistry [29,30,31,32,33,34]. This catalytic system possesses prominent merits over previously developed methodologies. First, Mn(III) is commercially accessible and low-cost, which circumvents the use of toxic catalysts or noble metals. Second, the transformation proceeds smoothly at room temperature under an ambient air atmosphere, without requiring inert gas protection or strong oxidants like TBHP. Third, THF, adopted herein, is a low-boiling-point solvent, which simplifies product isolation and favors large-scale synthesis. Fourth, the method can be further applied to the synthesis of rarely explored tellurophosphates. Collectively, this approach serves as a practical and eco-friendly complement to existing synthetic strategies for such compounds.

## 2. Results and Discussion

With these considerations in mind, we initiated our study by optimizing the reaction conditions for the coupling of dimethyl phosphonate (**1a**) with Ph_2_Se_2_ (**2a**). The results are summarized in Table 1. Initially, a series of manganese catalysts were screened using ethanol as the solvent at room temperature for 6 h (Table 1, entries 1–8). Among the catalysts examined, Mn(III) species exhibited markedly superior performance compared to Mn(II) salts, with Mn(OAc)_3_ displaying the highest catalytic activity and affording the product an isolated yield of 73% (entry 8).

With Mn(OAc)_3_ identified as the optimal catalyst, a range of solvents were subsequently evaluated (entries 9–16). The reaction proceeded with moderate-to-good yields across polar protic, polar aprotic, and nonpolar solvents. Among these, tetrahydrofuran (THF) proved to be the most effective solvent, delivering an isolated yield of 89% of the product (entry 16). Reducing the catalyst loading to 5 mol% or 2 mol% decreased yields (entries 17–18). Notably, extending the reaction time to 12 h at a catalyst loading value of 5 mol% Mn(OAc)_3_ afforded a comparable yield of 87% (entry 19). By contrast, a control experiment conducted in the absence of a manganese catalyst only produced a 26% yield (entry 20), confirming the essential role of Mn(OAc)_3_ in this transformation. Although a longer reaction time was required at lower catalyst loading, 5 mol% Mn(OAc)_3_ was selected as the standard condition for subsequent studies due to its increased cost-effectiveness and reduced environmental impact.

**Scheme 3:**
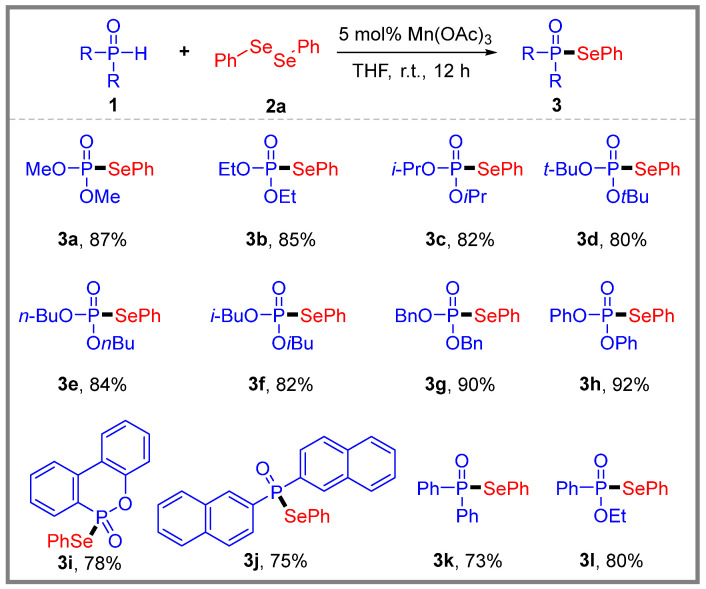
Substrate scope of phosphonates. Reaction conditions: **1** (0.20 mmol), **2a** (0.12 mmol), Mn(OAc)_3_ (0.010 mmol), and THF (2 mL), air, r.t., 12 h.

With the optimized conditions established, we next explored the substrate scope of phosphonates. As shown in Scheme 3, several dialkyl phosphonates bearing diverse alkyl substituents—including methyl (**3a**), ethyl (**3b**), isopropyl (**3c**), *tert*-butyl (**3d**), *n*-butyl (**3e**), isobutyl (**3f**), and benzyl (**3g**)—were well tolerated, affording good yields of the desired products. Notably, the sterically hindered *tert*-butyl-substituted phosphonate produced the product in 80% yield, highlighting the remarkable steric tolerance of this method. Diphenyl phosphonate also proved to be a suitable substrate, delivering the corresponding product in 92% yield (**3h**).

To further expand the scope, cyclic and structurally diverse P(III)-H compounds were examined. The cyclic H-phosphonate 9,10-dihydro-9-oxa-10-phosphaphenanthrene 10-oxide (DOPO) smoothly underwent the reaction to afford the selenylated product (**3i**) with a 78% yield. Additionally, both bis(2-naphthyl) phosphine oxide and diphenylphosphine oxide were converted efficiently to the corresponding products **3j** (75%) and **3k** (73%), respectively. Ethyl phenylphosphinate, an unsymmetrical compound bearing both ethoxy and phenyl groups, was also compatible with the reaction conditions (**3l**).

**Scheme 4:**
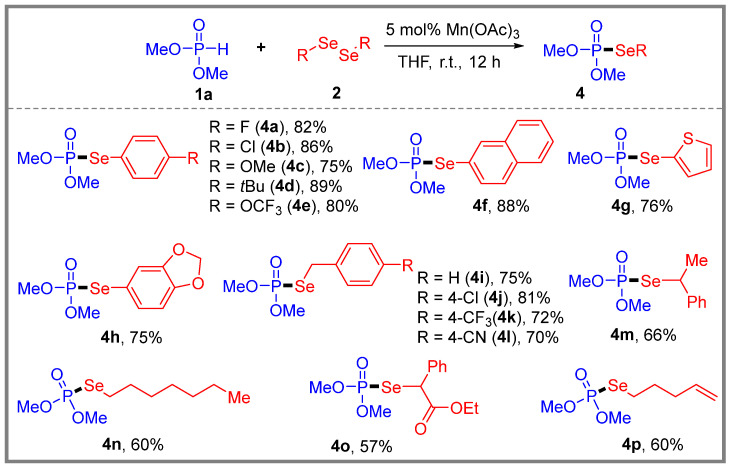
Substrate scope of diselenides. Reaction conditions: **1a** (0.20 mmol), **2** (0.12 mmol), Mn(OAc)_3_ (0.010 mmol), and THF (2 mL), air, r.t., 12 h.

Next, the scope of diselenides was investigated (Scheme 4). Aryl diselenides bearing either electron-withdrawing (**4a**–**4b**) or electron-donating groups (**4c**–**4e**) reacted well to afford good-to-excellent yields of the corresponding P–Se products, demonstrating excellent functional group tolerance. Heteroaryl diselenides, such as 2-naphthyl and 2-thiophene derivatives, also furnished the desired products (**4f** and **4g**) efficiently. Furthermore, a benzodioxole-derived diselenide afforded the selenylated product **4h** in 75% yield, expanding the structural diversity of the products.

Notably, although aliphatic diselenides are generally less reactive and less commonly employed in selenylation reactions, this protocol proved highly effective for various aliphatic diselenide substrates. As shown in Scheme 4, a series of aliphatic diselenides, including benzyl (**4i**–**4l**), secondary alkyl (**4m**), linear alkyl (**4n**), α-ester-substituted alkyl (**4o**), and terminal alkene-containing alkyl (**4p**) derivatives, participated in the reaction, affording the desired products in moderate-to-good yields.

**Scheme 5:**
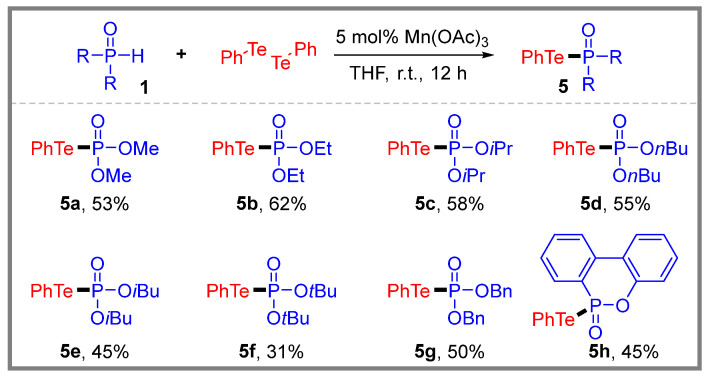
Synthesis of aryltellurophosphates. Reaction conditions: **1** (0.20 mmol), Ph_2_Te_2_ (0.12 mmol), Mn(OAc)_3_ (0.010 mmol), and THF (2 mL), air, r.t., 12 h.

As a congener of selenium, Te-aryl tellurophosphates have been far less frequently reported due to their inherent instability. Nevertheless, tellurophosphates are valuable chalcogen-substituted phosphorus compounds with potential biological and synthetic applications [44,45]. Following the successful synthesis of selenophosphates, we extended this methodology to the preparation of Te-aryl tellurophosphates. As shown in Scheme 5, the desired products were obtained in moderate yields. This reduced efficiency is primarily attributed to the instability of tellurium-containing compounds, which are prone to decomposition during column chromatography. Despite these limitations, this method provides a rare and practical route to tellurophosphates and a foundational step toward exploring their potential biological and synthetic applications.

**Scheme 6:**
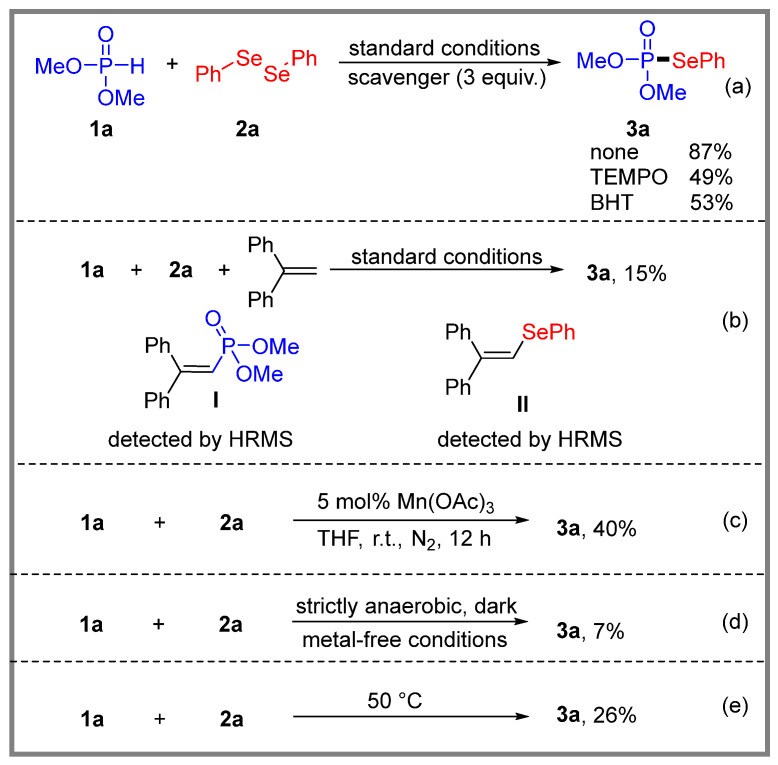
Control experiments for mechanistic studies.

To gain mechanistic insight, several control experiments were conducted (Scheme 6). Radical trapping experiments using TEMPO (2,2,6,6-tetramethyl-1-piperidinyloxy) and BHT (butylated hydroxytoluene) significantly suppressed product formation, indicating the involvement of a radical pathway (Scheme 6a). Furthermore, 1,1-diphenylethylene addition inhibited the reaction (Scheme 6b), and two adducts (**I** and **II**) were detected by ESI-MS, suggesting the generation of both phosphonyl and selenyl radicals during coupling. When the reaction of **1a** with **2a** was carried out under a nitrogen atmosphere, the yield of **3a** decreased to 40% (Scheme 6c), highlighting the crucial promoting role of molecular oxygen. Nevertheless, the relatively moderate yield (40%) under anaerobic conditions may result from incomplete removal of trace oxygen. More plausibly, however, a catalyst-free background reaction also contributes significantly.

Given that a 26% yield of **3a** was obtained in the absence of Mn(OAc)_3_ (Table 1, entry 20), a control experiment was conducted under strictly anaerobic, dark, and metal-free conditions, wherein all glassware was pre-cleaned with freshly prepared aqua regia and reaction vessels were fully wrapped with aluminum foil to exclude ambient light. Remarkably, trace quantities of product **3a** were still detected despite these rigorous experimental precautions (Scheme 6d). Furthermore, the target product could also be successfully afforded when the reaction was conducted at 50 °C under the aforementioned rigorous control conditions (Scheme 6e). These results unambiguously verify the existence of an intrinsic background radical pathway independent of exogenous radical initiators.

**Scheme 7:**
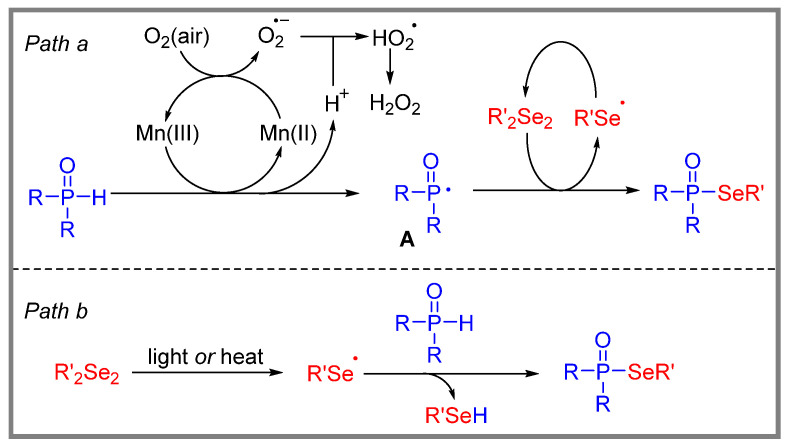
Proposed reaction mechanism.

Based on these results and previous reports, a plausible mechanism for the reaction of phosphonate with diselenide is proposed (Scheme 7). The reaction is initiated by Mn(III)-mediated single-electron oxidation of the phosphonate, generating phosphinyl radical **A** [40,41,42]. This radical is then trapped by the diselenide to form the desired selenophosphate and a selenyl radical. The selenyl radical can combine and undergo dimerization to regenerate diselenide, thereby sustaining the catalytic cycle. Meanwhile, reduced Mn(II) is reoxidized by atmospheric oxygen to regenerate the active Mn(III) species (Path a). An alternative mechanism for the synthesis of selenophosphates proceeding through a selenium radical pathway (path b), though not the major one, is also plausible.

## 3. Materials and Methods

### 3.1. General Information

All chemicals were of reagent grade and used without further purification unless otherwise noted. Solvents were dried and distilled prior to use, and petroleum ether (boiling point range: 60–90 °C) was used for chromatography. Reaction progress was monitored through thin-layer chromatography (TLC) on 60 precoated silica gel plates (Qingdao, China, 0.25 mm thickness), and the products were purified through flash column chromatography on silica gel (200−300 mesh). NMR spectra were recorded on a Bruker Avance-III HD spectrometer (Billerica, MA, USA, ^1^H NMR: 400 MHz, ^13^C NMR: 100 MHz), with chemical shifts reported in parts per million (ppm) relative to residual solvent peaks (CDCl_3_: 7.26 ppm and 77.16 ppm for ^1^H NMR and ^13^C NMR, respectively) and reported in parts per million (ppm) relative to tetramethylsilane (TMS) (see Supplymentary Meterials). Spin−spin coupling constants (*J*) were reported in Hz, and melting points were determined using glass slides and a WRX-4 digital (Shanghai, China) display microscopic melting point apparatus and left uncorrected. H-phosphonates were purchased from Bide (Shanghai, China) and Leyan (Shanghai, China)., whereas diselenides and ditellurides were prepared from the corresponding iodides with elemental selenium/tellurium according to a procedure from the listerature [46].

### 3.2. General Procedure for the Synthesis of Chalcogenophosphates

A 20 mL test tube equipped with a magnetic stir bar was charged with phosphite (0.2 mmol), dichalcogenide (0.12 mmol), Mn(OAc)_3_ (0.010 mmol), and THF (2 mL). The reaction mixture was stirred at room temperature under air for 12 h. Then, the solvent was removed with a rotary evaporator under reduced pressure. The crude product was purified through flash column chromatography on silica gel using petroleum ether/ethyl acetate as the eluent to afford the desired product.

***O*,*O*-Dimethyl *Se*-phenyl phosphoroselenoate** (**3a**). Compound **3a** was prepared according to the general procedure and isolated as an oil (46 mg, 87% yield) after flash chromatography (petroleum ether/ethyl acetate = 5/1). ^1^H NMR (400 MHz, CDCl_3_) δ 7.56 (d, *J* = 6.7 Hz, 2H), 7.44–6.97 (m, 3H), 3.73 (s, 3H), 3.69 (s, 3H). ^13^C NMR (100 MHz, CDCl_3_) δ 134.6 (d, *J*_C-P_ = 4.7 Hz), 128.6 (d, *J*_C-P_ = 2.2 Hz), 127.9 (d, *J*_C-P_ = 2.7 Hz), 122.2 (d, *J*_C-P_ = 8.5 Hz), 53.0 (d, *J*_C-P_ = 5.7 Hz). ^31^P NMR (162 MHz, CDCl_3_) δ 21.8. Spectral data are in good agreement with literature values [9].

***O*,*O*-Diethyl *Se*-phenyl phosphoroselenoate** (**3b**). Compound **3b** was prepared according to the general procedure and isolated as an oil (50 mg, 85% yield) after flash chromatography (petroleum ether/ethyl acetate = 5/1). ^1^H NMR (400 MHz, CDCl_3_) δ 7.66–7.63 (m, 2H), 7.47–7.17 (m, 3H), 4.40–3.75 (m, 4H), 1.31 (t, *J* = 7.1 Hz, 6H). ^13^C NMR (100 MHz, CDCl_3_) δ 134.5 (d, *J*_C-P_ = 4.6 Hz), 128.5 (d, *J*_C-P_ = 2.1 Hz), 127.8 (d, *J*_C-P_ = 2.6 Hz), 122.7 (d, *J*_C-P_ = 8.5 Hz), 62.8 (d, *J*_C-P_ = 5.9 Hz), 14.9 (d, *J*_C-P_ = 7.4 Hz). ^31^P NMR (162 MHz, CDCl_3_) δ 18.0. Spectral data are in good agreement with literature values [27].

***O,O*-Diisopropyl *Se*-phenyl phosphoroselenoate** (**3c**). Compound **3c** was prepared according to the general procedure and isolated as an oil (53 mg, 82% yield) after flash chromatography (petroleum ether/ethyl acetate = 5/1). ^1^H NMR (400 MHz, CDCl_3_) δ 7.59 (d, *J* = 8.1 Hz, 2H), 7.33–7.17 (m, 3H), 4.89–4.57 (m, 2H), 1.26 (d, *J* = 6.2 Hz, 6H), 1.18 (d, *J* = 6.2 Hz, 6H). ^13^C NMR (100 MHz, CDCl_3_) δ 134.1 (d, *J*_C-P_ = 5.0 Hz), 128.3 (d, *J*_C-P_ = 1.9 Hz), 127.5 (d, *J*_C-P_ = 2.5 Hz), 123.5 (d, *J*_C-P_ = 8.3 Hz), 72.1 (d, *J*_C-P_ = 6.6 Hz), 22.9 (d, *J*_C-P_ = 3.8 Hz), 22.5 (d, *J*_C-P_ = 5.9 Hz). ^31^P NMR (162 MHz, CDCl_3_) δ 14.7. Spectral data are in good agreement with literature values [9].

***O*,*O*-Di-*tert*-butyl *Se*-phenyl phosphoroselenoate** (**3d**). Compound **3d** was prepared according to the general procedure and isolated as an oil (56 mg, 80% yield) after flash chromatography (petroleum ether/ethyl acetate = 4/1). ^1^H NMR (400 MHz, CDCl_3_) δ 7.62 (d, *J =* 8.0 Hz, 2H), 7.46–7.02 (m, 3H), 1.39 (s, 18H). ^13^C NMR (100 MHz, CDCl_3_) δ 134.7 (d, *J*_C-P_ = 4.7 Hz), 128.0 (d, *J*_C-P_ = 2.2 Hz), 127.2 (d, *J*_C-P_ = 2.7 Hz), 124.9 (d, *J*_C-P_ = 8.9 Hz), 84.4 (d, *J*_C-P_ = 10.4 Hz), 29.2 (d, *J*_C-P_ = 4.4 Hz). ^31^P NMR (162 MHz, CDCl_3_) δ 5.0. Spectral data are in good agreement with literature values [34].

***O*,*O*-Dibutyl *Se*-phenyl phosphoroselenoate** (**3e**). Compound **3e** was prepared according to the general procedure and isolated as an oil (59 mg, 84% yield) after flash chromatography (petroleum ether/ethyl acetate = 5/1). ^1^H NMR (400 MHz, CDCl_3_) δ 7.57 (d, *J* = 8.4 Hz, 2H), 7.40–7.07 (m, 3H), 4.31–3.78 (m, 4H), 1.55 (dq, *J* = 8.2, 6.6 Hz, 4H), 1.38–1.21 (m, 4H), 0.82 (t, *J* = 7.4 Hz, 6H). ^13^C NMR (100 MHz, CDCl_3_) δ 134.5 (d, *J*_C-P_ = 4.7 Hz), 128.4 (d, *J*_C-P_ = 2.1 Hz), 127.7 (d, *J*_C-P_ = 2.5 Hz), 122.8 (d, *J*_C-P_ = 8.3 Hz), 66.5 (d, *J*_C-P_ = 6.5 Hz), 31.0 (d, *J*_C-P_ = 7.3 Hz), 17.7, 12.5. ^31^P NMR (162 MHz, CDCl_3_) δ 18.0. Spectral data are in good agreement with literature values [9].

***O*,*O*-Diisobutyl *Se*-phenyl phosphoroselenoate** (**3f**). Compound **3f** was prepared according to the general procedure and isolated as an oil (57 mg, 82% yield) after flash chromatography (petroleum ether/ethyl acetate = 5/1). ^1^H NMR (400 MHz, CDCl_3_) δ 7.58 (d, *J* = 6.6 Hz, 2H), 7.38–7.11 (m, 3H), 3.96–3.64 (m, 4H), 1.85 (dp, *J* = 13.3, 6.7 Hz, 2H), 0.83 (d, *J* = 1.6 Hz, 6H), 0.82 (d, *J* = 1.6 Hz, 6H). ^13^C NMR (100 MHz, CDCl_3_) δ 134.5 (d, *J*_C-P_ = 4.7 Hz), 128.4 (d, *J*_C-P_ = 1.9 Hz), 127.7 (d, *J*_C-P_ = 2.5 Hz), 122.7 (d, *J*_C-P_ = 8.5 Hz), 72.6 (d, *J*_C-P_ = 7.0 Hz), 27.9 (d, *J*_C-P_ = 7.6 Hz), 17.66, 17.65. ^31^P NMR (162 MHz, CDCl_3_) δ 17.8. Spectral data are in good agreement with literature values [27].

***O*,*O*-Dibenzyl *Se*-phenyl phosphoroselenoate** (**3g**). Compound **3g** was prepared according to the general procedure and isolated as an oil (75 mg, 90% yield) after flash chromatography (petroleum ether/ethyl acetate = 4/1). ^1^H NMR (400 MHz, CDCl_3_) δ 7.48 (d, *J* = 8.1 Hz, 2H), 7.30–7.21 (m, 7H), 7.21–7.11 (m, 6H), 5.07–4.98 (m, 4H). ^13^C NMR (100 MHz, CDCl_3_) δ 134.8 (d, *J*_C-P_ = 4.7 Hz), 134.2 (d, *J*_C-P_ = 7.8 Hz), 128.5 (d, *J*_C-P_ = 2.2 Hz), 127.9 (d, *J*_C-P_ = 2.7 Hz), 127.52, 127.50, 127.0, 122.2 (d, *J*_C-P_ = 8.6 Hz), 68.1 (d, *J*_C-P_ = 6.1 Hz). ^31^P NMR (162 MHz, CDCl_3_) δ 18.5. Spectral data are in good agreement with literature values [9].

***O*,*O*,*Se*-Triphenyl phosphoroselenoate** (**3h**). Compound **3h** was prepared according to the general procedure and isolated as an oil (72 mg, 92% yield) after flash chromatography (petroleum ether/ethyl acetate = 6/1). ^1^H NMR (400 MHz, CDCl_3_) δ 7.55–7.37 (m, 2H), 7.33–7.14 (m, 7H), 7.15–7.07 (m, 6H). ^13^C NMR (100 MHz, CDCl_3_) δ 149.2 (d, *J*_C-P_ = 8.6 Hz), 135.2 (d, *J*_C-P_ = 4.8 Hz), 128.7 (d, *J*_C-P_ = 1.3 Hz), 128.5 (d, *J*_C-P_ = 2.5 Hz), 128.3 (d, *J*_C-P_ = 2.9 Hz), 124.6 (d, *J*_C-P_ = 1.6 Hz), 121.6 (d, *J*_C-P_ = 9.1 Hz), 119.6 (d, *J*_C-P_ = 5.1 Hz). ^31^P NMR (162 MHz, CDCl_3_) δ 9.5. Spectral data are in good agreement with literature values [9].

**6-(Phenylselanyl)dibenzo[*c*,*e*][1,2]oxaphosphinine 6-oxid*e*** (**3i**). Compound **3i** was prepared according to the general procedure and isolated as an oil (59 mg, 78% yield) after flash chromatography (petroleum ether/ethyl acetate = 5/1). ^1^H NMR (400 MHz, CDCl_3_) δ 7.80 (dd, *J =* 14.9, 7.7 Hz, 1H), 7.69 (dd, *J =* 8.1, 6.5 Hz, 1H), 7.60–7.49 (m, 2H), 7.39 (tdd, *J =* 7.5, 3.6, 1.0 Hz, 1H), 7.31–7.20 (m, 1H), 7.17 (dq, *J =* 8.2, 2.1 Hz, 2H), 7.12–7.02 (m, 3H), 6.92 (t, *J =* 7.6 Hz, 2H). ^13^C NMR (100 MHz, CDCl_3_) δ 149.3 (d, *J*_C-P_ = 9.8 Hz), 135.9 (d, *J*_C-P_ = 3.7 Hz), 134.9 (d, *J*_C-P_ = 7.5 Hz), 132.7 (d, *J*_C-P_ = 2.7 Hz), 129.5 (t, *J*_C-P_ = 5.5 Hz), 128.0 (dd, *J*_C-P_ = 5.2, 2.7 Hz), 127.4 (d, *J*_C-P_ = 15.0 Hz), 125.6, 124.4, 123.9, 123.9, 123.7, 122.2 (d, *J* = 11.2 Hz), 120.9 (d, *J*_C-P_ = 7.1 Hz), 120.8 (d, *J*_C-P_ = 11.7 Hz), 119.0 (d, *J*_C-P_ = 7.0 Hz). ^31^P NMR (162 MHz, CDCl_3_) δ 31.4. Spectral data are in good agreement with literature values [27].

***Se*-Phenyl di(naphthalen-2-yl)phosphinoselenoate** (**3j**). Compound **3j** was prepared according to the general procedure and isolated as a solid (69 mg, 75% yield) after flash chromatography (petroleum ether/ethyl acetate = 2/1). Mp= 230–231 °C. ^1^H NMR (400 MHz, CDCl_3_) δ 8.39–8.31 (m, 2H), 7.90–7.73 (m, 6H), 7.53–7.43 (m, 7H), 7.20–7.01 (m, 4H). ^13^C NMR (100 MHz, CDCl_3_) δ 136.4 (d, *J* = 3.3 Hz), 134.8 (d, *J* = 2.7 Hz), 133.6 (d, *J* = 9.6 Hz), 132.4 (d, *J* = 14.4 Hz), 131.5, 130.9 (d, *J* = 5.8 Hz), 129.3 (d, *J* = 1.7 Hz), 129.2, 128.5, 128.4, 127.8, 127.7, 127.1, 126.1 (d, *J* = 12.0 Hz). ^31^P NMR (162 MHz, CDCl_3_) δ 40.0. Spectral data are in good agreement with literature values [47].

***Se*-Phenyl diphenylphosphinoselenoate** (**3k**). Compound **3k** was prepared according to the general procedure and isolated as an oil (52 mg, 73% yield) after flash chromatography (petroleum ether/ethyl acetate = 5/1). ^1^H NMR (400 MHz, CDCl_3_) δ 7.87–7.64 (m, 4H), 7.51–7.26 (m, 8H), 7.16 (t, *J* = 8.0 Hz, 1H), 7.07 (t, *J* = 7.5 Hz, 2H). ^13^C NMR (100 MHz, CDCl_3_) δ 136.3 (d, *J*_C-P_ = 3.3 Hz), 133.9, 133.0, 132.3 (d, *J*_C-P_ = 3.3 Hz), 131.4 (d, *J*_C-P_ = 10.6 Hz), 129.3 (d, *J*_C-P_ = 1.7 Hz), 128.5 (d, *J*_C-P_ = 13.2 Hz), 123.7 (d, *J*_C-P_ = 15.7 Hz). ^31^P NMR (162 MHz, CDCl_3_) δ 40.0. Spectral data are in good agreement with literature values [9].

***O*-Ethyl *Se*-phenyl phenylphosphonoselenoate** (**3l**). Compound **3l** was prepared according to the general procedure and isolated as an oil (52 mg, 80% yield) after flash chromatography (petroleum ether/ethyl acetate = 5/1). ^1^H NMR (400 MHz, CDCl_3_) δ 7.58–7.47 (m, 2H), 7.44–7.40 (m, 1H), 7.32–7.25 (m, 4H), 7.23–7.18 (m, 2H), 7.11 (t, *J* = 7.6 Hz, 1H), 4.39–4.18 (m, 2H), 1.34 (t, *J* = 7.1 Hz, 3H). ^13^C NMR (100 MHz, CDCl_3_) δ 136.5 (d, *J*_C-P_ = 3.6 Hz), 132.8 (d, *J*_C-P_ = 138.0 Hz), 132.5 (d, *J*_C-P_ = 3.3 Hz), 131.1 (d, *J*_C-P_ = 11.0 Hz), 129.4 (d, *J*_C-P_ = 2.2 Hz), 128.8 (d, *J*_C-P_ = 2.5 Hz), 128.2 (d, *J*_C-P_ = 14.9 Hz), 124.2 (d, *J*_C-P_ = 6.7 Hz), 62.6 (d, *J*_C-P_ = 7.1 Hz), 16.2 (d, *J*_C-P_ = 7.1 Hz). ^31^P NMR (162 MHz, CDCl_3_) δ 36.7. Spectral data are in good agreement with literature values [47].

***Se*-(4-Fluorophenyl) *O*,*O*-dimethyl phosphoroselenoate** (**4a**). Compound **4a** was prepared according to the general procedure and isolated as an oil (46 mg, 82% yield) after flash chromatography (petroleum ether/ethyl acetate = 3/1). ^1^H NMR (400 MHz, CDCl_3_) δ 7.54 (dd, *J* = 8.8, 5.2 Hz, 2H), 6.98–6.93 (m, 2H), 3.74 (s, 3H), 3.71 (s, 3H). ^13^C NMR (100 MHz, CDCl_3_) δ 162.3 (dd, *J*_C-F_ = 249.7, *J*_C-P_ = 3.0 Hz), 136.7 (dd, *J*_C-P_ = 8.3, *J*_C-P_ =4.4 Hz), 116.8 (dd, *J*_C-F_ = 8.7, *J*_C-P_ = 3.5 Hz), 115.9 (dd, *J*_C-F_ = 21.9, *J*_C-P_ = 2.3 Hz), 53.1 (d, *J*_C-P_ = 5.9 Hz). ^19^F NMR (376 MHz, CDCl_3_) δ −111.7 (d, *J*_P-F_ = 4.8 Hz). ^31^P NMR (162 MHz, CDCl_3_) δ 21.5 (d, *J*_P-F_ = 4.8 Hz). Spectral data are in good agreement with literature values [44].

***Se*-(4-Chlorophenyl) *O*,*O*-dimethyl phosphoroselenoate** (**4b**). Compound **4b** was prepared according to the general procedure and isolated as an oil (51 mg, 86% yield) after flash chromatography (petroleum ether/ethyl acetate = 5/1). ^1^H NMR (400 MHz, CDCl_3_) δ 7.50 (d, *J =* 8.5 Hz, 2H), 7.22 (d, *J =* 8.5 Hz, 2H), 3.74 (s, 3H), 3.71 (s, 3H). ^13^C NMR (100 MHz, CDCl_3_) δ 135.9 (d, *J*_C-P_ = 4.6 Hz), 134.5 (d, *J*_C-P_ = 3.1 Hz), 128.8 (d, *J*_C-P_ = 2.2 Hz), 120.3 (d, *J*_C-P_ = 8.8 Hz), 53.1 (d, *J*_C-P_ = 5.9 Hz). ^31^P NMR (162 MHz, CDCl_3_) δ 21.2. Spectral data are in good agreement with literature values [44].

***Se*-(4-Methoxyphenyl) *O*,*O*-dimethyl phosphoroselenoate** (**4c**). Compound **4c** was prepared according to the general procedure and isolated as an oil (44 mg, 75% yield) after flash chromatography (petroleum ether/ethyl acetate = 2/1). ^1^H NMR (400 MHz, CDCl_3_) δ 7.46 (d, *J* = 8.8 Hz, 2H), 6.78 (d, *J* = 8.8 Hz, 2H), 3.72 (s, 3H), 3.71 (s, 3H), 3.70 (s, 3H). ^13^C NMR (100 MHz, CDCl_3_) δ 159.4 (d, *J*_C-P_ = 2.8 Hz), 136.3 (d, *J*_C-P_ = 4.2 Hz), 114.3 (d, *J*_C-P_ = 2.4 Hz), 112.0 (d, *J*_C-P_ = 8.7 Hz), 54.3, 52.9 (d, *J*_C-P_ = 5.7 Hz). ^31^P NMR (162 MHz, CDCl_3_) δ 22.2. Spectral data are in good agreement with literature values [44].

***Se*-(4-(tert-Butyl)phenyl) *O*,*O*-dimethyl phosphoroselenoate** (**4d**). Compound **4d** was prepared according to the general procedure and isolated as an oil (57 mg, 89% yield) after flash chromatography (petroleum ether/ethyl acetate = 3/1). ^1^H NMR (400 MHz, CDCl_3_) δ 7.47 (dd, *J* = 8.5 Hz, 2H), 7.26 (d, *J* = 8.5 Hz, 2H), 3.75 (s, 3H), 3.71 (s, 3H), 1.23 (s, 9H). ^13^C NMR (100 MHz, CDCl_3_) δ 151.2 (d, *J*_C-P_ = 2.9 Hz), 134.3 (d, *J*_C-P_ = 4.4 Hz), 125.8 (d, *J*_C-P_ = 2.3 Hz), 118.5 (d, *J*_C-P_ = 8.6 Hz), 52.9 (d, *J*_C-P_ = 5.6 Hz), 30.2. ^31^P NMR (162 MHz, CDCl_3_) δ 22.3. Spectral data are in good agreement with literature values [34].

***O*,*O*-Dimethyl *Se*-(4-(trifluoromethoxy)phenyl) phosphoroselenoate** (**4e**). Compound **4e** was prepared according to the general procedure and isolated as an oil (56 mg, 80% yield) after flash chromatography (petroleum ether/ethyl acetate = 3/1). ^1^H NMR (400 MHz, CDCl_3_) δ 7.60 (dd, *J* = 8.8 Hz, 2H), 7.10 (d, *J* = 8.8 Hz, 2H), 3.75 (s, 3H), 3.72 (s, 3H). ^13^C NMR (100 MHz, CDCl_3_) δ 151.32–145.90 (m), 136.1 (d, *J*_C-P_ = 4.6 Hz), 120.9 (d, *J*_C-P_ = 2.1 Hz), 120.4 (d, *J*_C-P_ = 8.6 Hz), 119.3 (q, *J*_C-F_ = 256.0 Hz), 53.1 (d, *J*_C-P_ = 5.9 Hz). ^19^F NMR (376 MHz, CDCl3) δ −57.9. ^31^P NMR (162 MHz, CDCl_3_) δ 21.0. Spectral data are in good agreement with literature values [33].

***O*,*O*-Dimethyl *Se*-(naphthalen-2-yl) phosphoroselenoate** (**4f**). Compound **4f** was prepared according to the general procedure and isolated as an oil (55 mg, 88% yield) after flash chromatography (petroleum ether/ethyl acetate = 3/1). ^1^H NMR (400 MHz, CDCl_3_) δ 8.38 (d, *J* = 8.4 Hz, 1H), 8.04–7.84 (m, 1H), 7.86–7.80 (m, 1H), 7.79–7.74 (m, 1H), 7.53 (ddd, *J* = 8.4, 6.8, 1.4 Hz, 1H), 7.45 (ddd, *J* = 8.1, 6.9, 1.2 Hz, 1H), 7.34 (t, *J* = 7.7 Hz, 1H), 3.66 (s, 3H), 3.63 (s, 3H). ^13^C NMR (100 MHz, CDCl_3_) δ 135.5 (d, *J*_C-P_ = 4.9 Hz), 133.9 (d, *J*_C-P_ = 3.3 Hz), 133.2 (d, *J*_C-P_ = 2.2 Hz), 129.4 (d, *J*_C-P_ = 3.2 Hz), 127.7, 126.9, 126.2, 125.5, 124.9 (d, *J*_C-P_ = 3.2 Hz), 121.6 (d, *J*_C-P_ = 9.4 Hz), 53.1 (d, *J*_C-P_ = 6.0 Hz). ^31^P NMR (162 MHz, CDCl_3_) δ 21.2. Spectral data are in good agreement with literature values [9].

***O*,*O*-Dimethyl *Se*-(thiophen-2-yl) phosphoroselenoate** (**4g**). Compound **4g** was prepared according to the general procedure and isolated as an oil (41 mg, 76% yield) after flash chromatography (petroleum ether/ethyl acetate = 3/1). ^1^H NMR (400 MHz, CDCl_3_) δ 7.39 (ddd, *J* = 5.4, 2.6, 1.2 Hz, 1H), 7.20 (td, *J* = 3.3, 1.2 Hz, 1H), 6.95 (dd, *J* = 5.3, 3.6 Hz, 1H), 3.77 (s, 3H), 3.74 (s, 3H). ^13^C NMR (100 MHz, CDCl_3_) δ 136.3 (d, *J*_C-P_ = 5.8 Hz), 131.1 (d, *J*_C-P_ = 3.8 Hz), 127.3 (d, *J*_C-P_ = 3.3 Hz), 114.7 (d, *J*_C-P_ = 10.5 Hz), 53.2 (d, *J*_C-P_ = 5.5 Hz). ^31^P NMR (162 MHz, CDCl_3_) δ 20.2. Spectral data are in good agreement with literature values [34].

***Se*-(Benzo[*d*][1,3]dioxol-5-yl) *O*,*O*-dimethyl phosphoroselenoate** (**4h**). Compound **4h** was prepared according to the general procedure and isolated as an oil (46 mg, 75% yield) after flash chromatography (petroleum ether/ethyl acetate = 2/1). ^1^H NMR (400 MHz, CDCl_3_) δ 7.21–7.02 (m, 2H), 6.77 (d, *J* = 8.0 Hz, 1H), 5.99 (s, 2H), 3.82 (s, 3H), 3.79 (s, 3H). ^13^C NMR (100 MHz, CDCl_3_) δ 148.8 (d, *J*_C-P_ = 3.0 Hz), 148.4 (d, *J*_C-P_ = 2.5 Hz), 130.1 (d, *J*_C-P_ = 5.3 Hz), 113.9 (d, *J*_C-P_ = 9.0 Hz), 109.4 (d, *J*_C-P_ = 2.7 Hz), 101.9, 54.0 (d, *J*_C-P_ = 5.7 Hz). ^31^P NMR (162 MHz, CDCl_3_) δ 22.2. Spectral data are in good agreement with literature values [34].

***Se*-Benzyl *O*,*O*-dimethyl phosphoroselenoate** (**4i**). Compound **4i** was prepared according to the general procedure and isolated as an oil (42 mg, 75% yield) after flash chromatography (petroleum ether/ethyl acetate = 5/1). ^1^H NMR (400 MHz, CDCl_3_) δ 7.30–7.15 (m, 5H), 4.00 (d, *J* = 12.9 Hz, 2H), 3.61 (d, *J* = 13.3 Hz, 6H). ^13^C NMR (100 MHz, CDCl_3_) δ 138.3 (d, *J*_C-P_ = 4.5 Hz), 129.0, 128.7, 127.5, 53.55 (d, *J*_C-P_ = 5.3 Hz), 29.4 (d, *J*_C-P_ = 4.6 Hz). ^31^P NMR (162 MHz, CDCl_3_) δ 24.6. Spectral data are in good agreement with literature values [16].

***Se*-(4-Chlorobenzyl) *O*,*O*-dimethyl phosphoroselenoate** (**4j**). Compound **4j** was prepared according to the general procedure and isolated as an oil (51 mg, 81% yield) after flash chromatography (petroleum ether/ethyl acetate = 4/1). ^1^H NMR (400 MHz, CDCl_3_) δ 7.23 (d, *J* = 8.8 Hz, 2H), 7.20 (d, *J* = 8.8 Hz, 2H), 3.97 (d, *J* = 13.4 Hz, 2H), 3.62 (d, *J* = 13.3 Hz, 6H). ^13^C NMR (100 MHz, CDCl_3_) δ 137.0 (d, *J*_C-P_ = 4.2 Hz), 133.3, 130.3, 128.8, 53.6 (d, *J*_C-P_ = 5.5 Hz), 28.6 (d, *J*_C-P_ = 4.6 Hz). ^31^P NMR (162 MHz, CDCl_3_) δ 24.1. Spectral data are in good agreement with literature values [33].

***O,O*-Dimethyl *Se*-(4-(trifluoromethyl)benzyl) phosphoroselenoate** (**4k**). Compound **4k** was prepared according to the general procedure and isolated as an oil (50 mg, 72% yield) after flash chromatography (petroleum ether/ethyl acetate = 5/1). ^1^H NMR (400 MHz, CDCl_3_) δ 7.49 (d, *J* = 8.2 Hz, 2H), 7.41 (d, *J* = 8.1 Hz, 2H), 4.02 (d, *J* = 13.8 Hz, 2H), 3.61 (d, *J* = 13.3 Hz, 6H). ^13^C NMR (100 MHz, CDCl_3_) δ 144.14–141.47 (m), 129.4 (q, *J*_C-P_ = 44.0 Hz), 129.3, 125.6 (q, *J* = 3.8 Hz), 121.3 (q, *J*_C-F_ = 274.0 Hz), 53.6 (d, *J* = 5.6 Hz), 28.4 (d, *J* = 4.7 Hz). ^19^F NMR (376 MHz, CDCl_3_) δ −62.6. ^31^P NMR (162 MHz, CDCl_3_) δ 23.7. Spectral data are in good agreement with literature values [33].

***Se*-(4-Cyanobenzyl) *O*,*O*-dimethyl phosphoroselenoate** (**4l**). Compound **4l** was prepared according to the general procedure and isolated as a solid (43 mg, 70% yield) after flash chromatography (petroleum ether/ethyl acetate = 3/1). Mp= 62–63 °C. ^1^H NMR (400 MHz, CDCl_3_) δ 7.54 (d, *J* = 8.0 Hz, 2H), 7.42 (d, *J* = 8.0 Hz, 2H), 4.01 (d, *J* = 14.2 Hz, 2H), 3.61 (d, *J* = 13.3 Hz, 6H). ^13^C NMR (100 MHz, CDCl_3_) δ 144.3 (d, *J*_C-P_ = 3.7 Hz), 132.4, 129.7, 118.6, 111.1, 53.7 (d, *J*_C-P_ = 5.6 Hz), 28.5 (d, *J*_C-P_ = 4.7 Hz). ^31^P NMR (162 MHz, CDCl_3_) δ 23.3. Spectral data are in good agreement with literature values [33].

***O*,*O*-Dimethyl *Se*-(1-phenylethyl) phosphoroselenoate** (**4m**). Compound **4m** was prepared according to the general procedure and isolated as an oil (39 mg, 66% yield) after flash chromatography (petroleum ether/ethyl acetate = 4/1). ^1^H NMR (400 MHz, CDCl_3_) δ 7.35–7.30 (m, 2H), 7.25 (t, *J* = 7.6 Hz, 2H), 7.22–7.14 (m, 1H), 4.83–4.45 (m, 1H), 3.57 (dd, *J* = 13.3, 3.2 Hz, 6H), 1.84 (d, *J* = 7.2 Hz, 3H). ^13^C NMR (100 MHz, CDCl_3_) δ 143.6 (d, *J*_C-P_ = 5.4 Hz), 128.6, 127.6, 127.1, 53.5 (dd, *J*_C-P_ = 5.4, 3.4 Hz), 42.9 (d, *J*_C-P_ = 4.3 Hz), 24.3 (d, *J*_C-P_ = 5.9 Hz). ^31^P NMR (162 MHz, CDCl_3_) δ 24.6. Spectral data are in good agreement with literature values [33].

***Se*-Heptyl *O*,*O*-dimethyl phosphoroselenoate** (**4n**). Compound **4n** was prepared according to the general procedure and isolated as an oil (34 mg, 60% yield) after flash chromatography (petroleum ether/ethyl acetate = 3/1). ^1^H NMR (400 MHz, CDCl_3_) δ 3.73 (s, 3H), 3.70 (s, 3H), 2.80 (dt, *J* = 14.1, 7.4 Hz, 2H), 1.75–1.58 (m, 2H), 1.40–1.12 (m, 8H), 0.81 (t, *J* = 6.5 Hz, 3H). ^13^C NMR (100 MHz, CDCl_3_) δ 52.5 (d, *J*_C-P_ = 5.6 Hz), 30.6, 30.2 (d, *J*_C-P_ = 4.5 Hz), 28.5, 27.6, 25.6 (d, *J*_C-P_ = 4.6 Hz), 21.5, 13.0. ^31^P NMR (162 MHz, CDCl_3_) δ 25.4. Spectral data are in good agreement with literature values [33].

**Ethyl 2-((Dimethoxyphosphoryl)selanyl)-2-phenylacetate** (**4o**). Compound **4o** was prepared according to the general procedure and isolated as an oil (40 mg, 57% yield) after flash chromatography (petroleum ether/ethyl acetate = 3/1). ^1^H NMR (400 MHz, CDCl_3_) δ 7.47–7.39 (m, 2H), 7.31–7.19 (m, 3H), 4.96 (d, *J* = 10.0 Hz, 1H), 4.28–4.00 (m, 2H), 3.56 (dd, *J* = 13.4, 5.2 Hz, 6H), 1.19 (t, *J* = 7.2 Hz, 3H). ^13^C NMR (100 MHz, CDCl_3_) δ 170.2 (d, *J*_C-P_ = 6.2 Hz), 136.6 (d, *J*_C-P_ = 3.9 Hz), 128.8, 128.6, 128.5, 62.4, 53.7 (t, *J*_C-P_ = 4.7 Hz), 46.9 (d, *J*_C-P_ = 3.8 Hz), 14.0. ^31^P NMR (162 MHz, CDCl_3_) δ 23.1. Spectral data are in good agreement with literature values [33].

***O*,*O*-Dimethyl *Se*-(pent-4-en-1-yl) phosphoroselenoate** (**4p**). Compound **4p** was prepared according to the general procedure and isolated as an oil (31 mg, 60% yield) after flash chromatography (petroleum ether/ethyl acetate = 1/1). ^1^H NMR (400 MHz, CDCl_3_) δ 5.70 (ddt, *J* = 16.9, 10.2, 6.7 Hz, 1H), 5.05–4.64 (m, 2H), 3.72 (d, *J* = 13.2 Hz, 6H), 2.93–2.71 (m, 2H), 2.18–2.01 (m, 2H), 1.86–1.76 (m, 2H). ^13^C NMR (100 MHz, CDCl_3_) δ 137.0, 115.8, 53.6 (d, *J*_C-P_ = 5.6 Hz), 33.5, 30.3 (d, *J*_C-P_ = 4.3 Hz), 25.8 (d, *J*_C-P_ = 4.7 Hz). ^31^P NMR (162 MHz, CDCl_3_) δ 25.1. Spectral data are in good agreement with literature values [33].

***O*,*O*-Dimethyl *Te*-phenyl phosphorotelluroate** (**5a**). Compound **5a** was prepared according to the general procedure and isolated as an oil (33 mg, 53% yield) after flash chromatography (petroleum ether/ethyl acetate = 2/1). ^1^H NMR (400 MHz, CDCl_3_) δ 7.76 (dt, *J* = 8.1, 1.5 Hz, 2H), 7.33–7.23 (m, 1H), 7.18 (t, *J* = 7.6 Hz, 2H), 3.67 (s, 3H), 3.63 (s, 3H). ^13^C NMR (100 MHz, CDCl_3_) δ 139.0 (d, *J*_C-P_ = 3.9 Hz), 128.9 (d, *J*_C-P_ = 2.0 Hz), 128.0 (d, *J*_C-P_ = 2.3 Hz), 107.3 (d, *J*_C-P_ = 8.1 Hz), 52.7 (d, *J*_C-P_ = 5.4 Hz). ^31^P NMR (162 MHz, CDCl_3_) δ 3.6. Spectral data are in good agreement with literature values [34].

***O*,*O*-Diethyl *Te*-phenyl phosphorotelluroate** (**5b**). Compound **5b** was prepared according to the general procedure and isolated as an oil (42 mg, 62% yield) after flash chromatography (petroleum ether/ethyl acetate = 2/1). ^1^H NMR (400 MHz, CDCl_3_) δ 7.84 (dt, *J* = 8.1, 1.5 Hz, 2H), 7.36 (td, *J* = 7.3, 1.4 Hz, 1H), 7.25 (t, *J* = 7.6 Hz, 2H), 4.21–4.09 (m, 4H), 1.31 (t, *J* = 7.1 Hz, 6H). ^13^C NMR (100 MHz, CDCl_3_) δ 139.9 (d, *J*_C-P_ = 3.9 Hz), 129.7 (d, *J*_C-P_ = 1.9 Hz), 128.9 (d, *J*_C-P_ = 2.3 Hz), 108.9 (d, *J*_C-P_ = 8.0 Hz), 63.5 (d, *J*_C-P_ = 5.5 Hz), 15.8 (d, *J*_C-P_ = 7.5 Hz). ^31^P NMR (162 MHz, CDCl_3_) δ 0.9. Spectral data are in good agreement with literature values [22].

***O*,*O*-Diisopropyl *Te*-phenyl phosphorotelluroate** (**5c**) Compound **5c** was prepared according to the general procedure and isolated as an oil (43 mg, 58% yield) after flash chromatography (petroleum ether/ethyl acetate = 2/1). ^1^H NMR (400 MHz, CDCl_3_) δ 7.84 (dt, *J* = 8.1, 1.5 Hz, 2H), 7.34 (td, *J* = 7.4, 1.5 Hz, 1H), 7.26 (d, *J* = 8.0 Hz, 2H), 4.86–4.76 (m, 2H), 1.35 (d, *J* = 6.2 Hz, 6H), 1.26 (d, *J* = 6.1 Hz, 6H). ^13^C NMR (100 MHz, CDCl_3_) δ 139.5 (d, *J*_C-P_ = 4.2 Hz), 129.6 (d, *J*_C-P_ = 1.8 Hz), 128.7 (d, *J*_C-P_ = 2.2 Hz), 109.7 (d, *J*_C-P_ = 8.0 Hz), 72.7 (d, *J*_C-P_ = 6.1 Hz), 23.9 (d, *J*_C-P_ = 3.1 Hz), 23.5 (d, *J*_C-P_ = 6.2 Hz). ^31^P NMR (162 MHz, CDCl_3_) δ −5.2. Spectral data are in good agreement with literature values [20].

***O*,*O*-Dibutyl *Te*-phenyl phosphorotelluroate** (**5d**). Compound **5d** was prepared according to the general procedure and isolated as an oil (44 mg, 55% yield) after flash chromatography (petroleum ether/ethyl acetate = 3/1). ^1^H NMR (400 MHz, CDCl_3_) δ 7.76 (d, *J* = 8.1, 1.5 Hz, 2H), 7.29 (td, *J* = 7.4, 1.5 Hz, 1H), 7.23–7.03 (m, 2H), 4.17–3.80 (m, 4H), 1.73–1.42 (m, 4H), 1.33–1.07 (m, 4H), 0.82 (t, *J* = 7.4 Hz, 6H). ^13^C NMR (100 MHz, CDCl_3_) δ 139.9 (d, *J*_C-P_ = 4.0 Hz), 129.6 (d, *J*_C-P_ = 1.9 Hz), 128.9 (d, *J*_C-P_ = 2.2 Hz), 108.9 (d, *J*_C-P_ = 8.0 Hz), 67.2 (d, *J*_C-P_ = 6.1 Hz), 31.9 (d, *J*_C-P_ = 7.3 Hz), 18.8, 13.6. ^31^P NMR (162 MHz, CDCl_3_) δ −0.9. Spectral data are in good agreement with literature values [34].

***O*,*O*-Diisobutyl *Te*-phenyl phosphorotelluroate** (**5e**). Compound **5e** was prepared according to the general procedure and isolated as an oil (36 mg, 45% yield) after flash chromatography (petroleum ether/ethyl acetate = 5/1). ^1^H NMR (400 MHz, CDCl_3_) δ 7.89–7.71 (m, 2H), 7.42–7.31 (m, 1H), 7.24 (t, *J* = 7.5 Hz, 2H), 3.90–3.84 (m, 2H), 3.82–3.75 (m, 2H), 1.93 (dt, *J* = 13.3, 6.7 Hz, 2H), 0.90 (d, *J* = 2.1 Hz, 6H), 0.88 (d, *J* = 2.1 Hz, 6H). ^13^C NMR (100 MHz, CDCl_3_) δ 140.0 (d, *J*_C-P_ = 4.0 Hz), 129.6 (d, *J*_C-P_ = 1.9 Hz), 128.9 (d, *J*_C-P_ = 2.2 Hz), 108.7 (d, *J*_C-P_ = 8.0 Hz), 73.3 (d, *J*_C-P_ = 6.8 Hz), 28.8 (d, *J*_C-P_ = 7.5 Hz), 18.8 (d, *J*_C-P_ = 3.2 Hz). ^31^P NMR (162 MHz, CDCl_3_) δ −1.1. Spectral data are in good agreement with literature values [26].

***O*,*O*-Di-*tert*-butyl *Te*-phenyl phosphorotelluroate** (**5f**). Compound **5f** was prepared according to the general procedure and isolated as an oil (25 mg, 31% yield) after flash chromatography (petroleum ether/ethyl acetate = 5/1). ^1^H NMR (400 MHz, CDCl_3_) δ 7.94–7.86 (m, 2H), 7.34 (td, *J* = 7.3, 1.8 Hz, 1H), 7.28–7.17 (m, 2H), 1.47 (s, 18H). ^13^C NMR (100 MHz, CDCl_3_) δ 140.0 (d, *J*_C-P_ = 4.1 Hz), 129.3 (d, *J*_C-P_ = 2.0 Hz), 128.4 (d, *J*_C-P_ = 2.3 Hz), 111.3 (d, *J*_C-P_ = 8.5 Hz), 85.5 (d, *J*_C-P_ = 11.4 Hz), 30.5 (d, *J*_C-P_ = 4.3 Hz). ^31^P NMR (162 MHz, CDCl_3_) δ −21.2. HRMS: m/z [M + H]^+^ calcd for C_14_H_24_O_3_PTe, 401.0520; found, 401.0526.

***O*,*O*-Dibenzyl *Te*-phenyl phosphorotelluroate** (**5g**). Compound **5g** was prepared according to the general procedure and isolated as an oil (47 mg, 50% yield) after flash chromatography (petroleum ether/ethyl acetate = 5/1). ^1^H NMR (400 MHz, CDCl_3_) δ 7.68 (d, *J* = 8.0 Hz, 2H), 7.34–7.22 (m, 7H), 7.21–7.16 (m, 4H), 7.10 (t, *J* = 7.6 Hz, 2H), 5.01 (d, *J* = 3.0 Hz, 2H), 4.99 (d, *J* = 3.4 Hz, 2H). ^13^C NMR (100 MHz, CDCl_3_) δ 139.1 (d, *J*_C-P_ = 4.0 Hz), 134.2 (d, *J*_C-P_ = 7.9 Hz), 128.6 (d, *J*_C-P_ = 2.1 Hz), 128.0 (d, *J*_C-P_ = 2.5 Hz), 127.5 (d, *J*_C-P_ = 2.5 Hz), 127.0, 107.5 (d, *J*_C-P_ = 8.1 Hz), 67.7 (d, *J*_C-P_ = 5.9 Hz). ^31^P NMR (162 MHz, CDCl_3_) δ −1.8. Spectral data are in good agreement with literature values [34].

**6-(Phenyltellanyl)dibenzo[*c*,*e*][1,2]oxaphosphinine 6-oxide** (**5h**). Compound **5h** was prepared according to the general procedure and isolated as an oil (38 mg, 45% yield) after flash chromatography (petroleum ether/ethyl acetate = 5/1). ^1^H NMR (400 MHz, CDCl_3_) δ 7.84 (dd, *J* = 14.8, 7.6 Hz, 1H), 7.69 (t, *J* = 7.1 Hz, 1H), 7.65–7.54 (m, 2H), 7.48 (td, *J* = 7.4, 3.5 Hz, 1H), 7.39 (dt, *J* = 8.0, 1.4 Hz, 2H), 7.35–7.31 (m, 1H), 7.16 (dd, *J* = 8.8, 5.9 Hz, 3H), 6.91 (t, *J* = 7.5 Hz, 2H). ^13^C NMR (100 MHz, CDCl_3_) δ 149.9 (d, *J*_C-P_ = 9.9 Hz), 141.3 (d, *J*_C-P_ = 2.9 Hz), 135.3 (d, *J*_C-P_ = 7.7 Hz), 133.6 (d, *J*_C-P_ = 2.9 Hz), 130.5, 130.0 (d, *J*_C-P_ = 11.7 Hz), 129.2 (d, *J*_C-P_ = 2.4 Hz), 128.9 (d, *J*_C-P_ = 2.6 Hz), 128.4 (d, *J*_C-P_ = 14.7 Hz), 124.9 (d, *J*_C-P_ = 13.7 Hz), 123.1 (d, *J*_C-P_ = 10.5 Hz), 122.2 (d, *J*_C-P_ = 12.0 Hz), 120.3 (d, *J*_C-P_ = 6.7 Hz), 108.4. ^31^P NMR (162 MHz, CDCl_3_) δ 14.8. HRMS: m/z [M + H]^+^ calcd for C_18_H_14_O_2_PTe, 422.9788; found, 422.9786.

## 4. Conclusions

In summary, we developed a mild, efficient, and environmentally friendly Mn(III)-catalyzed radical coupling method for the synthesis of selenophosphates and tellurophosphates. Using commercially available Mn(OAc)_3_ as the catalyst and THF as the solvent, a wide range of H-phosphonates and H-phosphine oxides reacted smoothly with diselenides or ditellurides at room temperature under air, affording the corresponding chalcogenophosphates in high yields. This protocol features a broad substrate scope, excellent functional group tolerance, and low catalyst loading. Mechanistic studies indicate that the reaction proceeds via a radical pathway involving phosphinyl radicals generated through Mn(III)-mediated single-electron oxidation. We anticipate that this method will expand the synthetic toolbox for chalcogenophosphates, with ongoing work in our laboratory focused on manganese-catalyzed construction of C–Se bonds.

## Figures and Tables

**Table 1:** Optimization of reaction conditions *^a^*.

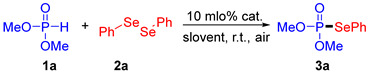			
Entry	Cat.	Solvent	Isolated Yield (%)
1	MnCl_2_	EtOH	35
2	MnBr_2_	EtOH	29
3	Mn(OTf)_2_	EtOH	23
4	MnO_2_	EtOH	13
5	Mn(OAc)_2_	EtOH	34
6	Mn(acac)_2_	EtOH	30
7	Mn(acac)_3_	EtOH	61
8	Mn(OAc)_3_	EtOH	73
9	Mn(OAc)_3_	MeOH	65
10	Mn(OAc)_3_	DMSO	37
11	Mn(OAc)_3_	CHCl_3_	20
12	Mn(OAc)_3_	CH_3_CN	52
13	Mn(OAc)_3_	CH_3_NO_2_	35
14	Mn(OAc)_3_	EtOAc	46
15	Mn(OAc)_3_	acetone	41
16	Mn(OAc)_3_	THF	89
17 *^b^*	Mn(OAc)_3_	THF	65
18 *^c^*	Mn(OAc)_3_	THF	40
19 *^d^*	Mn(OAc)_3_	THF	87
20	-	THF	26

*^a^* Reaction conditions: **1a** (0.20 mmol, 1.00 equiv), **2a** (0.12 mmol, 0.60 equiv), cat. (0.020 mmol, 0.10 equiv), solvent (2 mL), r.t., 6 h. *^b^* 5 mol% Mn(OAc)_3_. *^c^* 2 mol% Mn(OAc)_3_. *^d^* 5 mol% Mn(OAc)_3_, 12 h.

## Data Availability

All the data and material described in this work are available in this article or in the Supplementary Materials.

## Supplementary Materials

The following supporting information can be downloaded at: https://www.mdpi.com/article/10.3390/molecules31122103/s1, Figures S1–S111, including NMR spectra of the obtained compounds.
