# Supplementary material for: Mn(III)-Catalyzed Synthesis of Selenophosphates and Tellurophosphates
Source: Molecules. 2026 Jun 15;31(12):2103. doi: 10.3390/molecules31122103 (PMC13305054; doi:10.3390/molecules31122103)
Supplement: Supplementary file 1 [file molecules-31-02103-s001.zip › molecules-4331939-supplementary.pdf]

Supporting Information

Mn(III)-Catalyzed Synthesis of Selenophosphates and Tellurophosphates

Jialu Wang, Jun Liu, Yuhui Xie, Changjiang Wu, Xinyu Wang and Gong-Qing Liu *

Organoselenium Synthesis and Function Laboratory, School of Pharmacy, Nantong University, Nantong 226019, People's Republic of China.

E-mail: gqliu@ntu.edu.cn

Content

1. HRMS experiments...S2

2. Copies of NMR spectra...S3

1. HRMS experiments

A solution of dimethyl phosphonate **1a** (0.20 mmol), Ph_2Se_2 **2a** (0.12 mmol), 1,1-diphenylethylene (0.60 mmol), $Mn(OAc)_3$ (0.010 mmol) and THF (2 mL) was placed in 20-mL test tube. The resulting mixture was stirred at room temperature under air for 12 h. a 15% yield of **3a** was detected, and two adducts were identified by HRMS analysis.

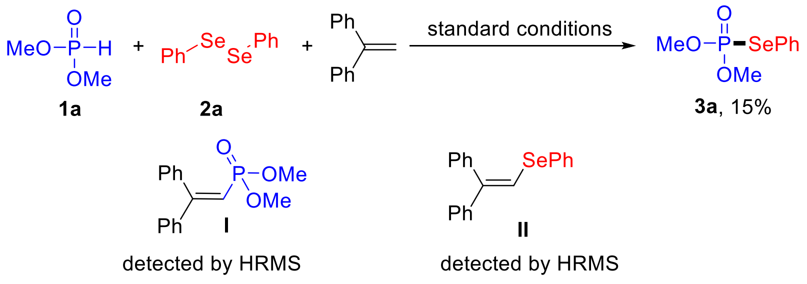

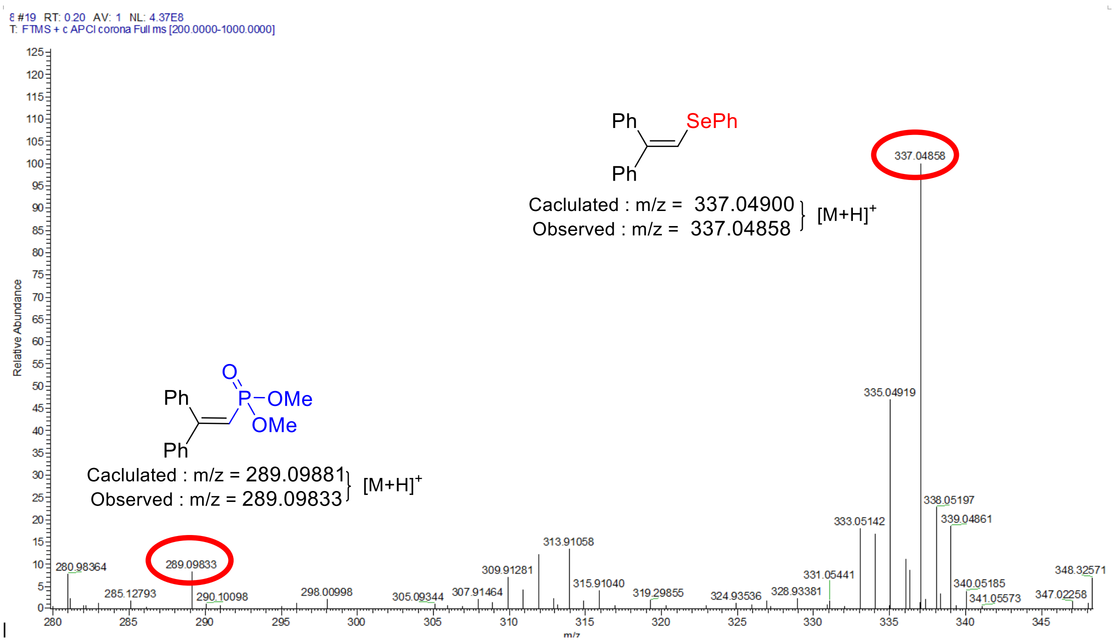

2. Copies of NMR spectra

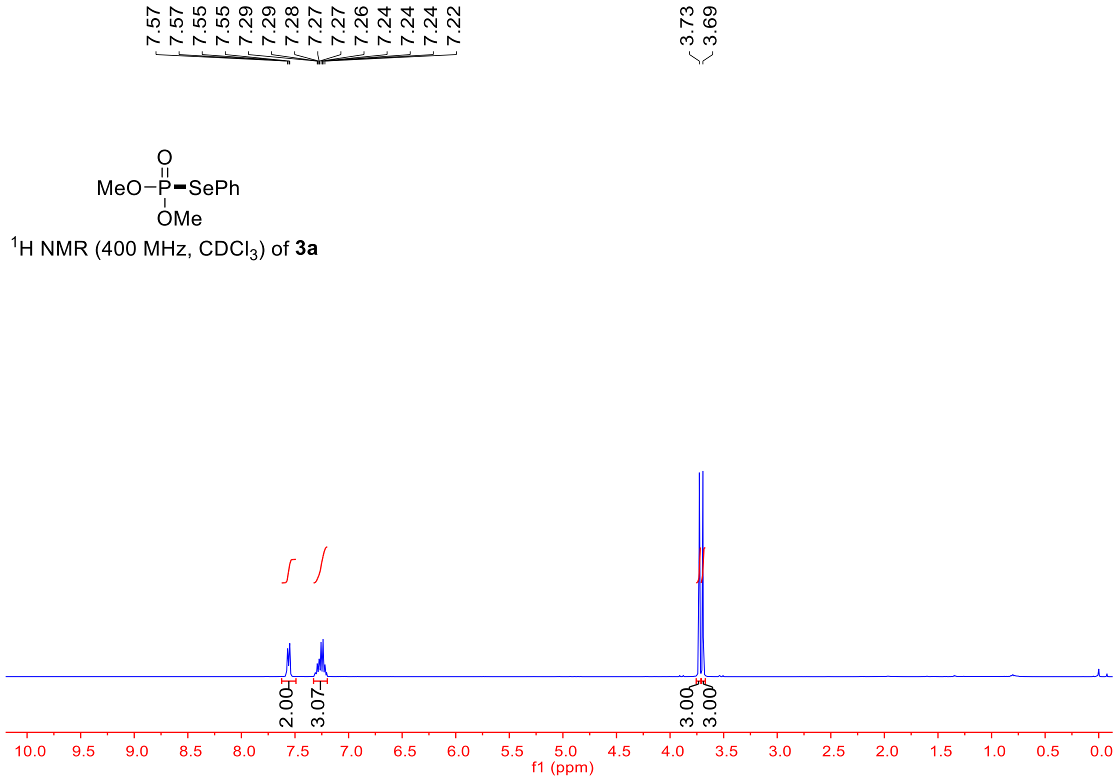

Figure S1: ^{1}H NMR spectrum for compound **3a**

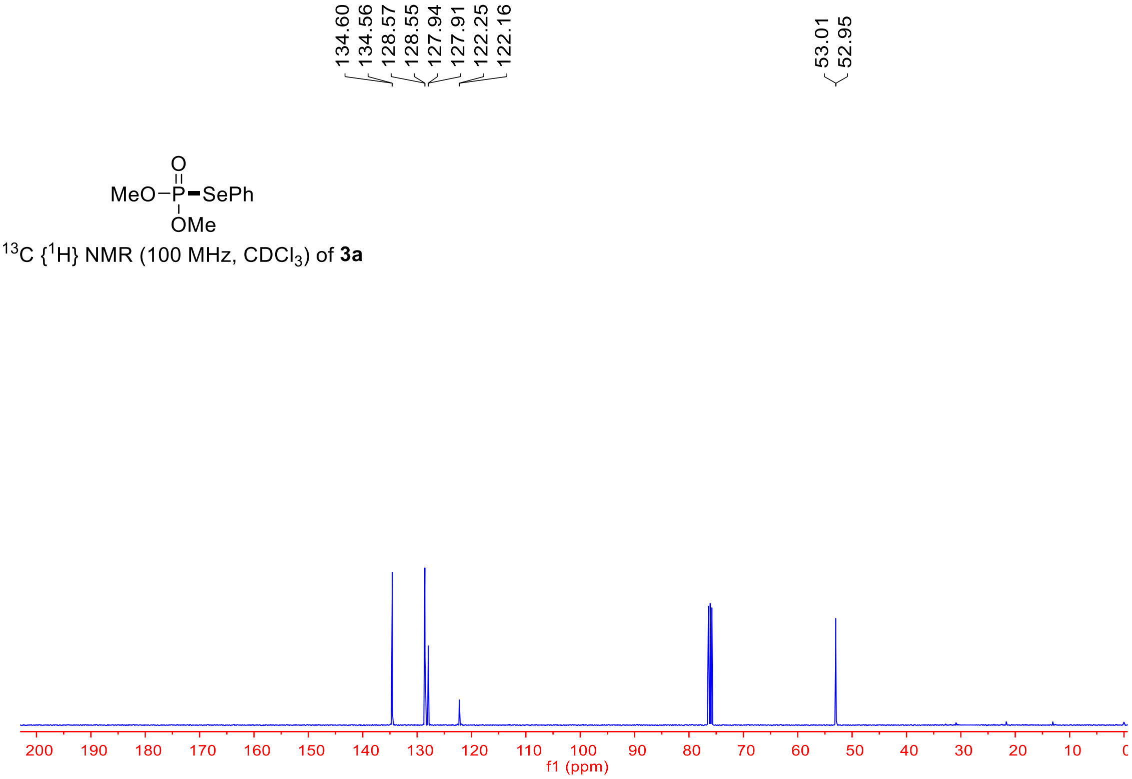

Figure S2: ^{13}C NMR spectrum for compound **3a**

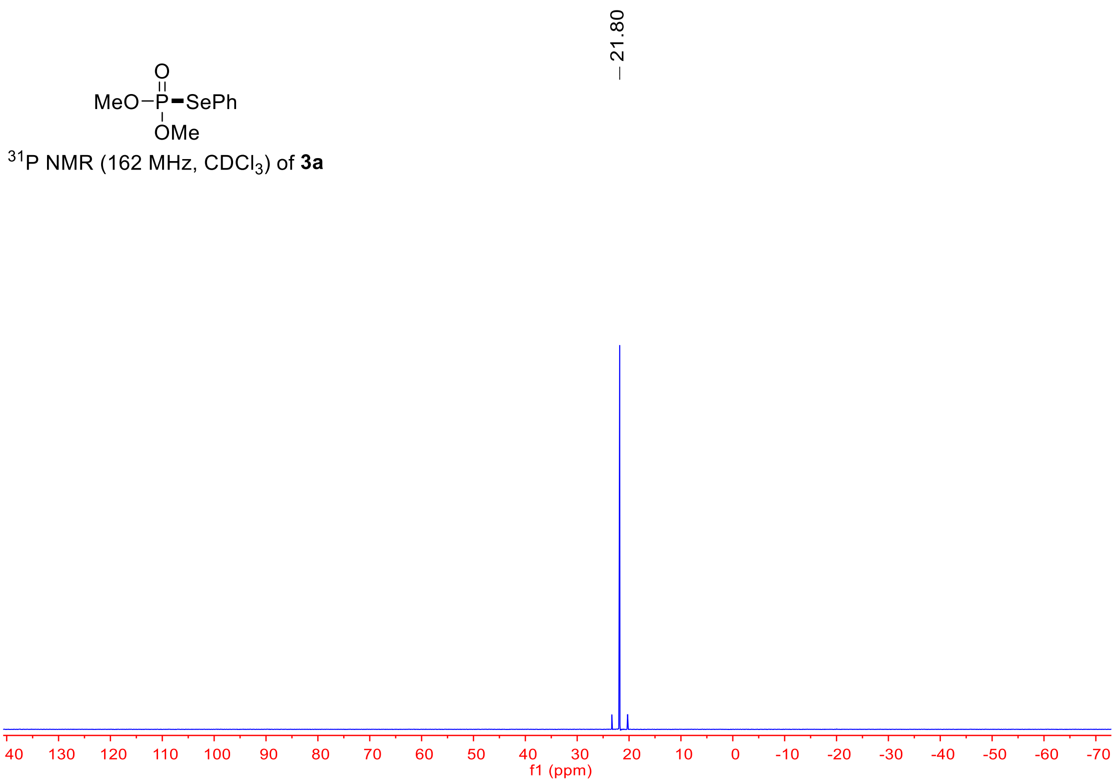

Figure S3: ^{31}P NMR spectrum for compound **3a**

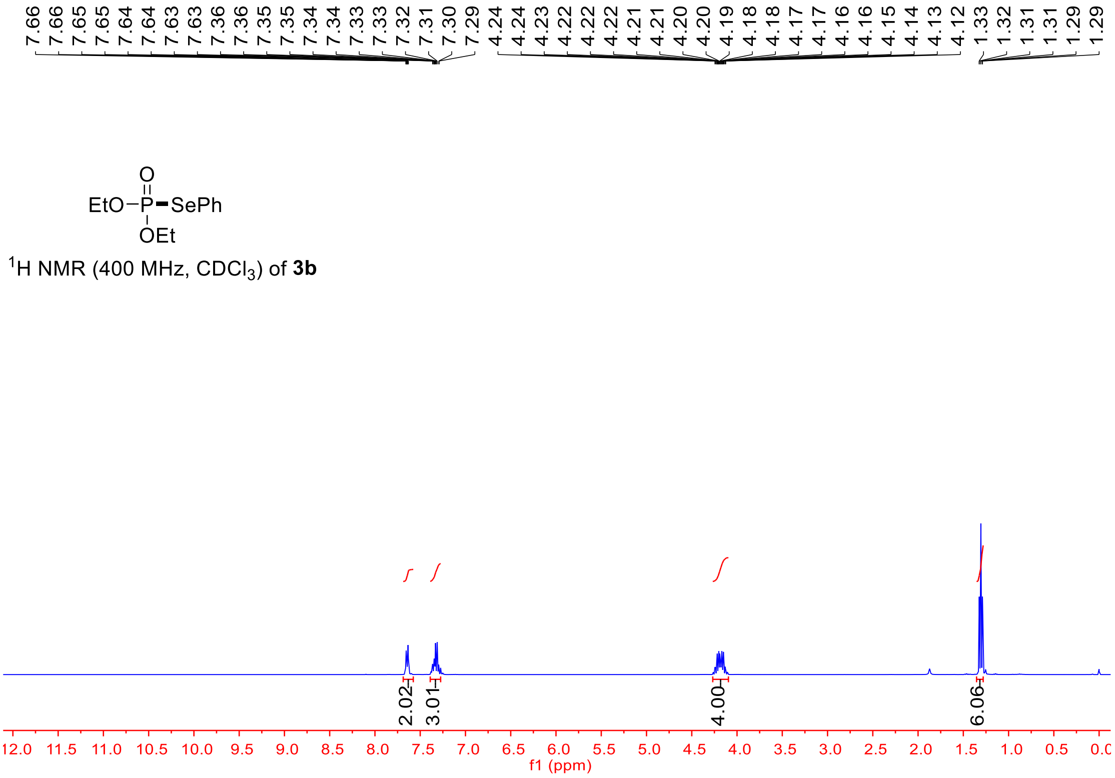

Figure S4: ^{1}H NMR spectrum for compound **3b**

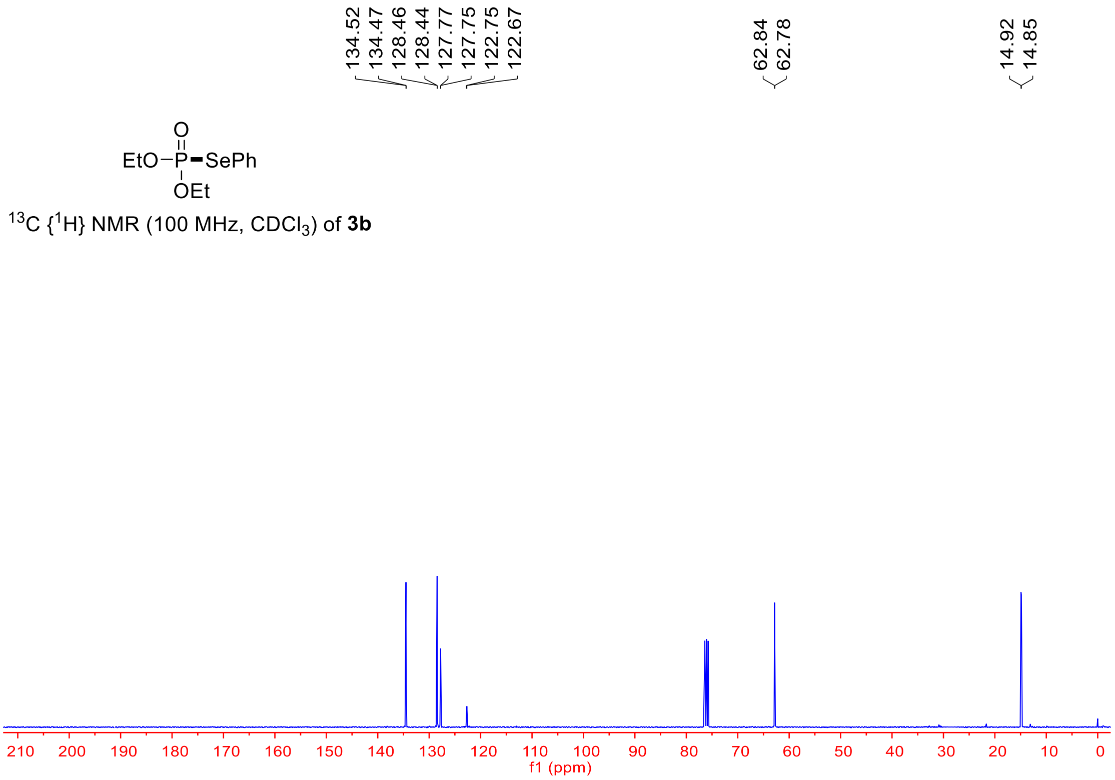

Figure S5: ^{13}C NMR spectrum for compound **3b**

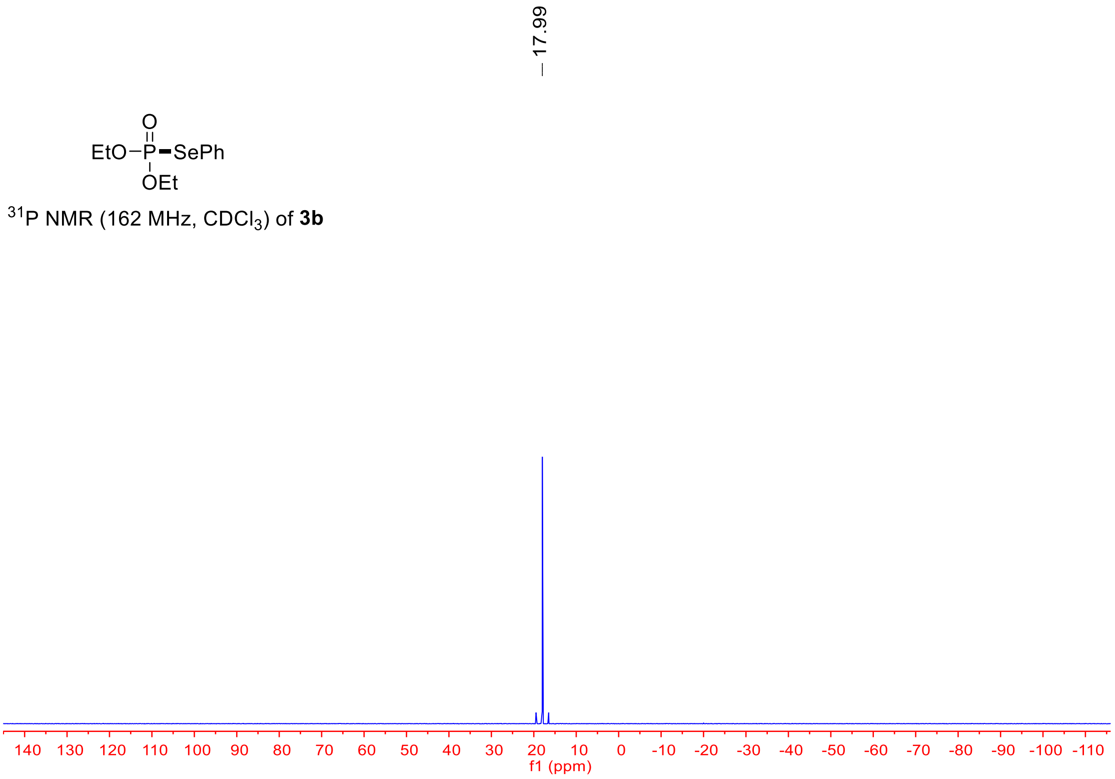

Figure S6: ^{31}P NMR spectrum for compound **3b**

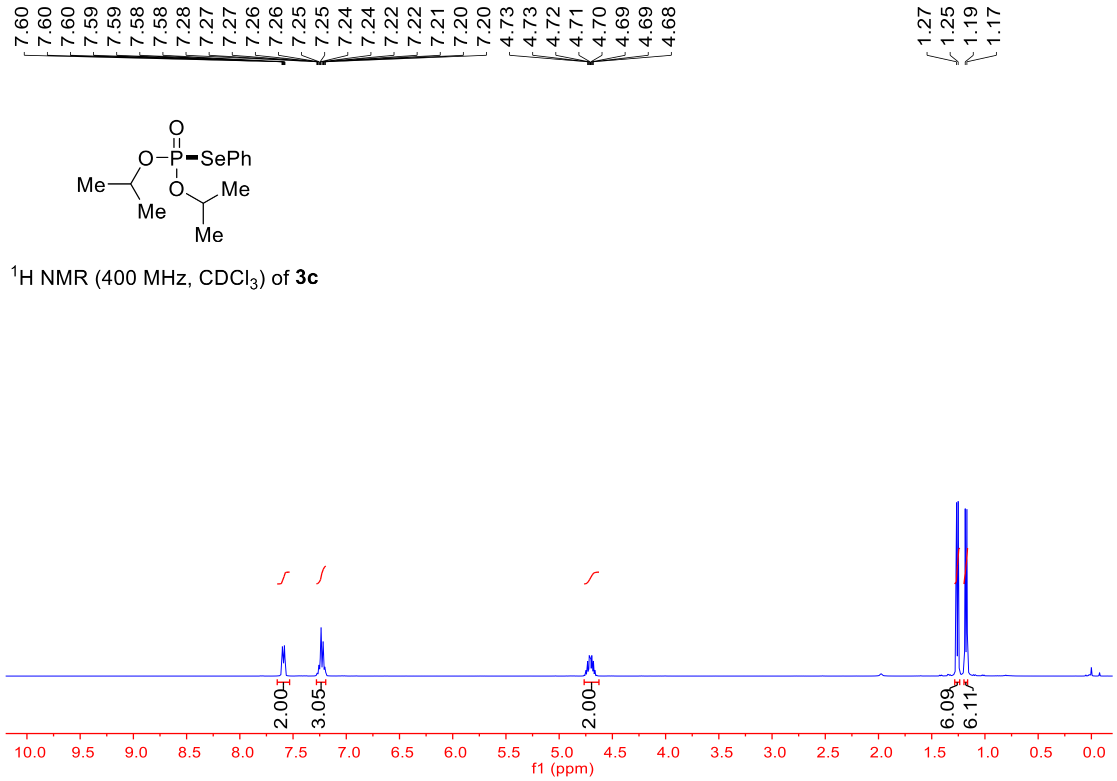

Figure S7: ^{1}H NMR spectrum for compound **3c**

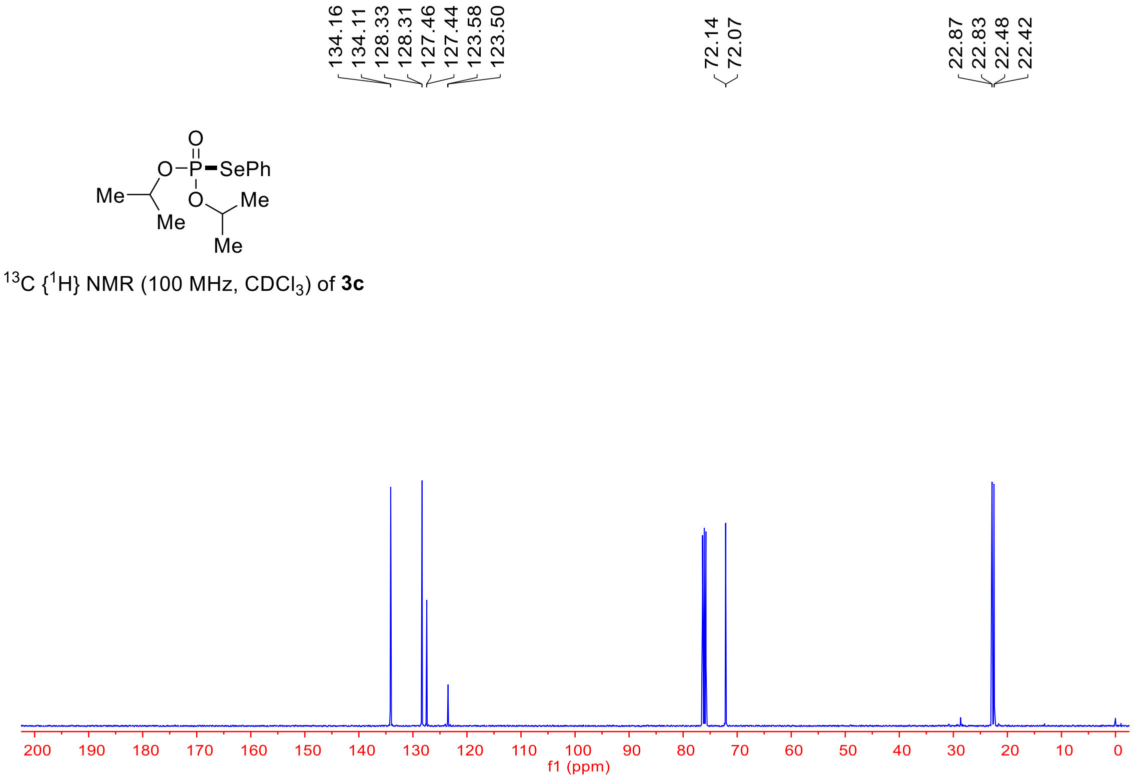

Figure S8: ^{13}C NMR spectrum for compound **3c**

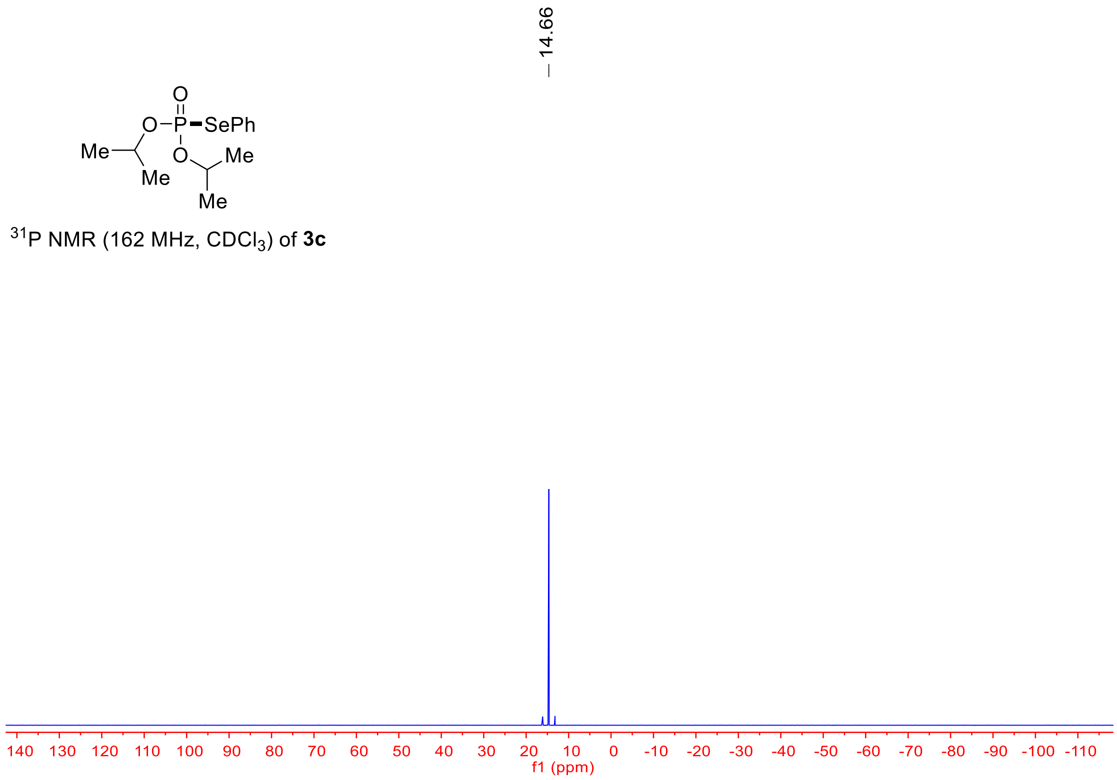

Figure S9: ^{31}P NMR spectrum for compound **3c**

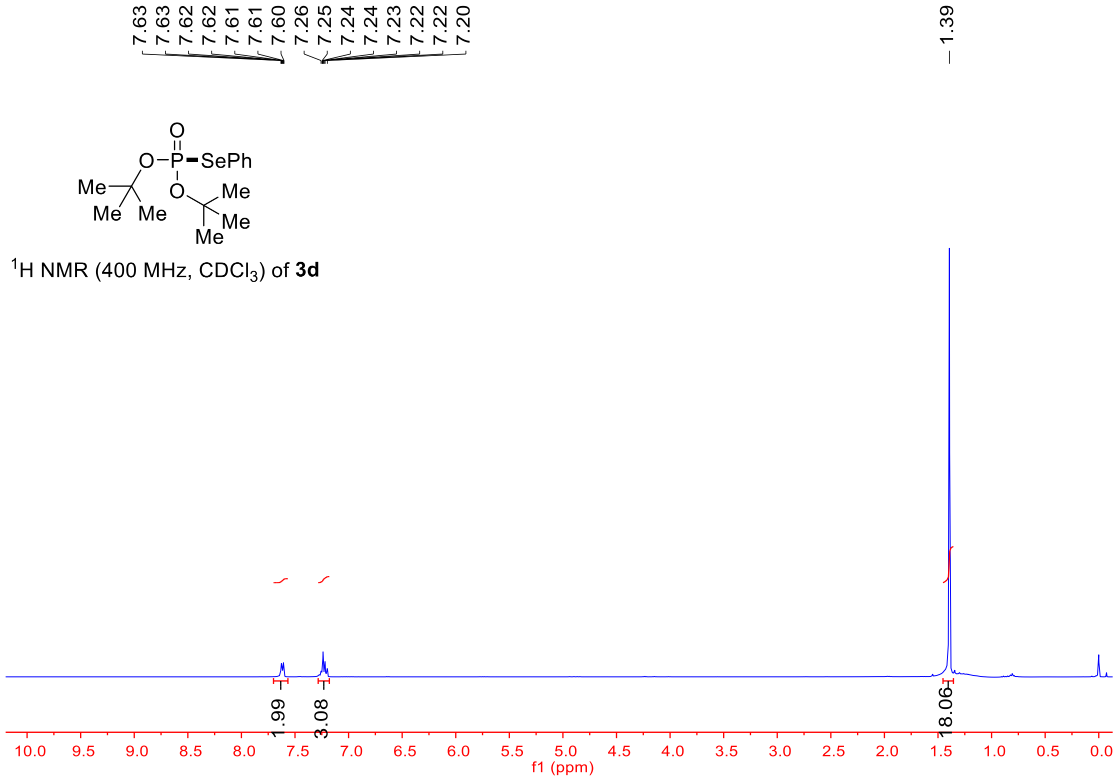

Figure S10: ^{1}H NMR spectrum for compound **3d**

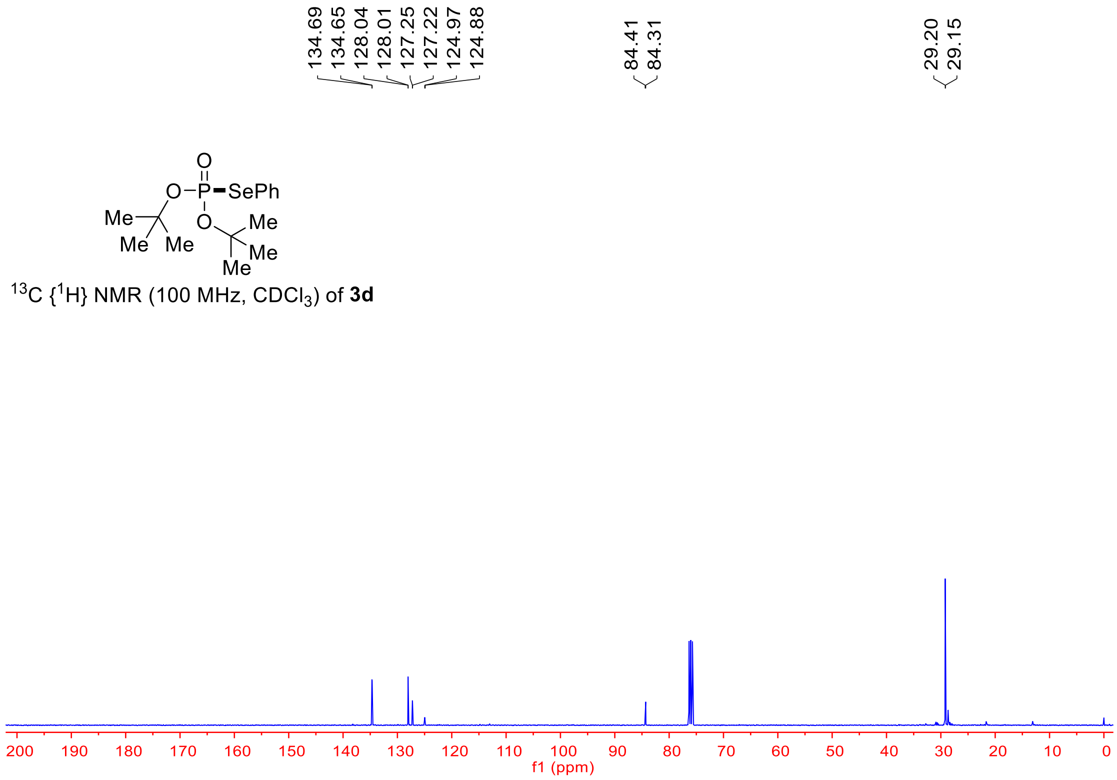

Figure S11: ^{13}C NMR spectrum for compound **3d**

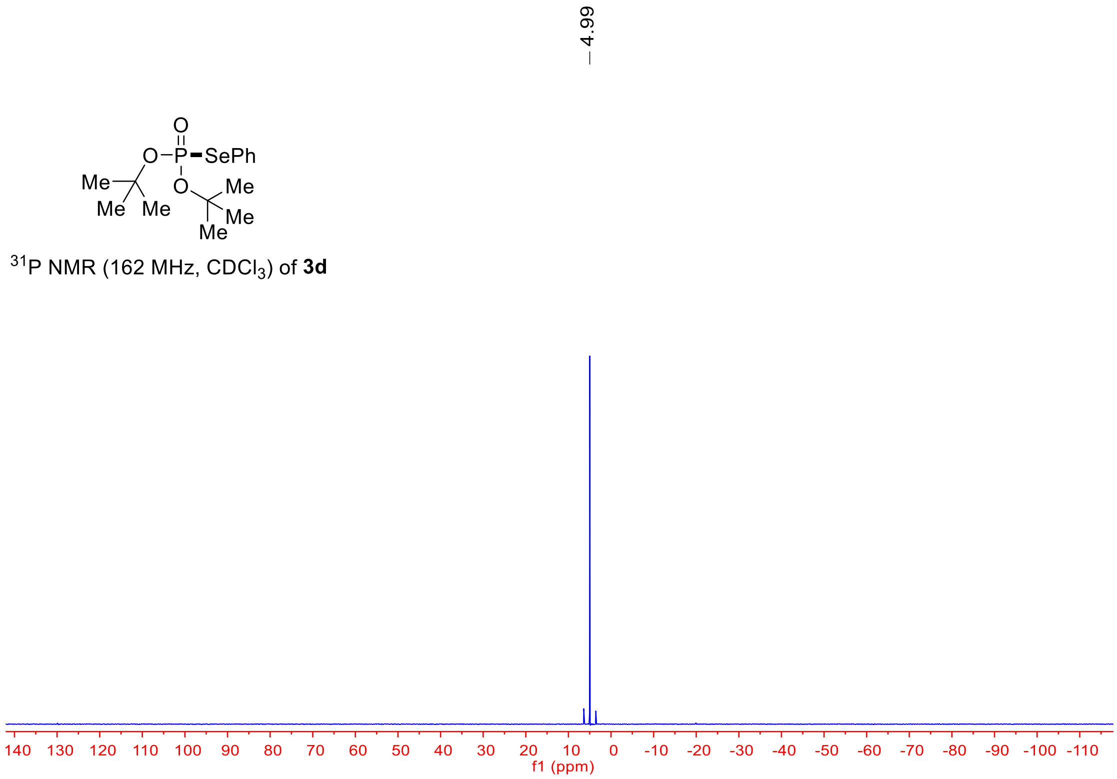

Figure S12: ^{31}P NMR spectrum for compound **3d**

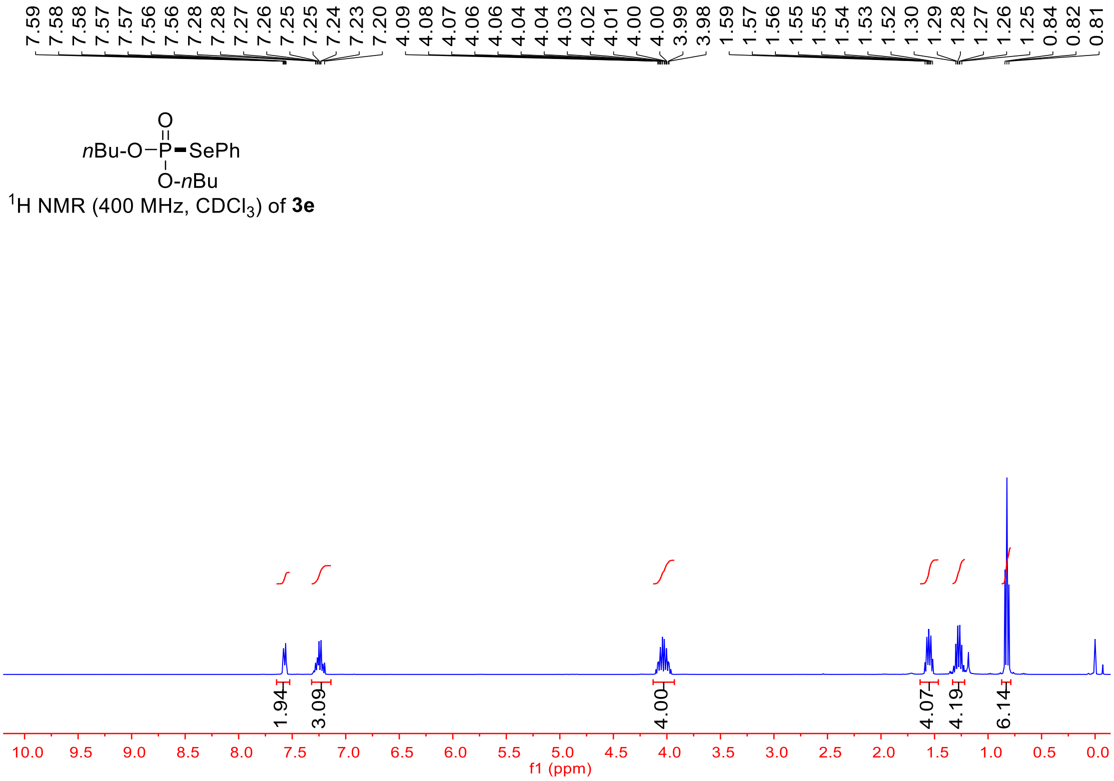

Figure S13: ^{1}H NMR spectrum for compound **3e**

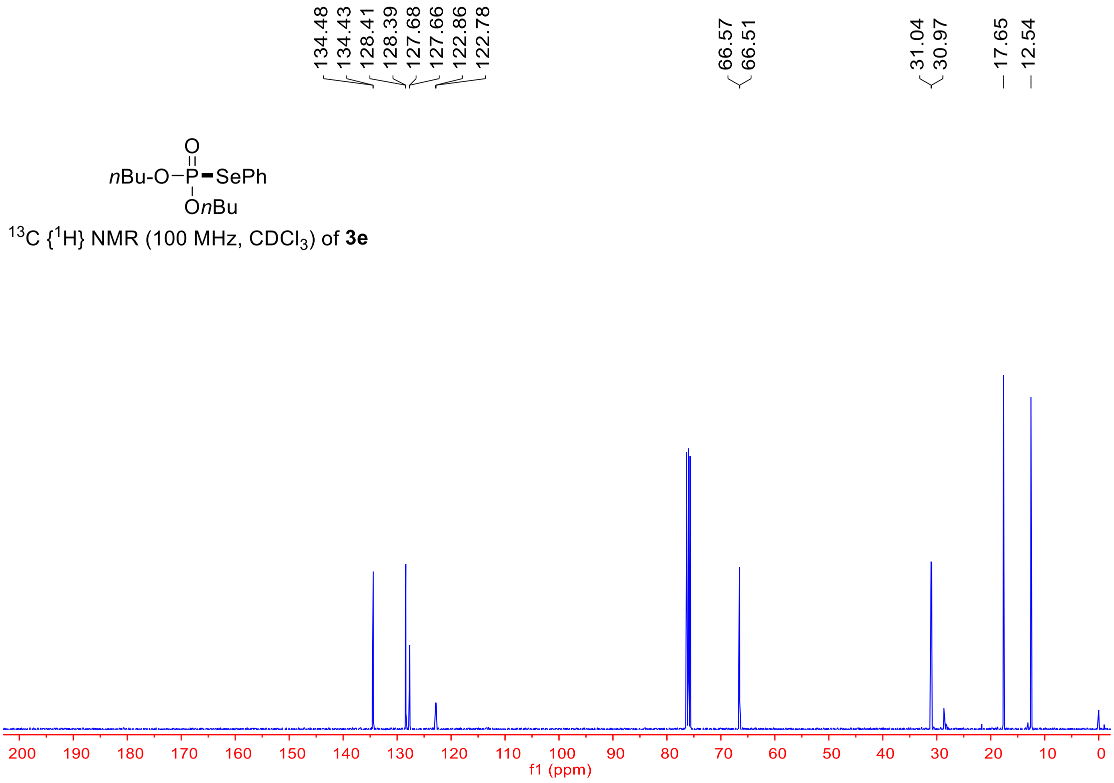

Figure S14: ^{13}C NMR spectrum for compound **3e**

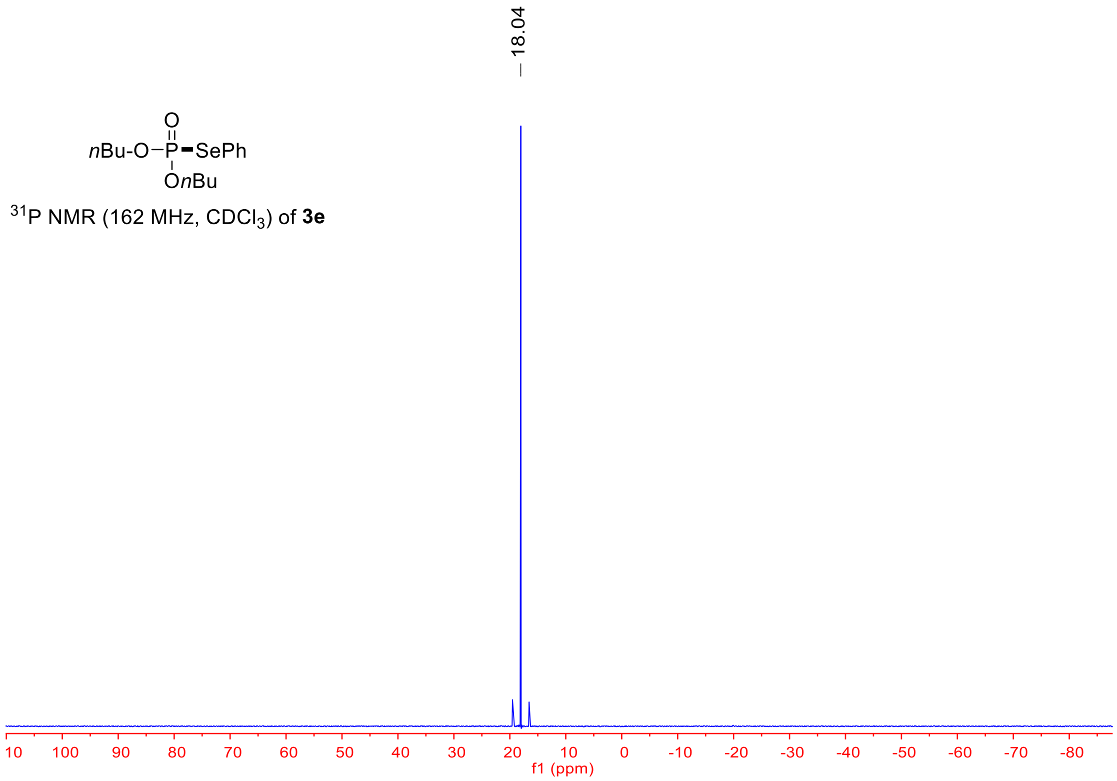

Figure S15: ^{31}P NMR spectrum for compound **3e**

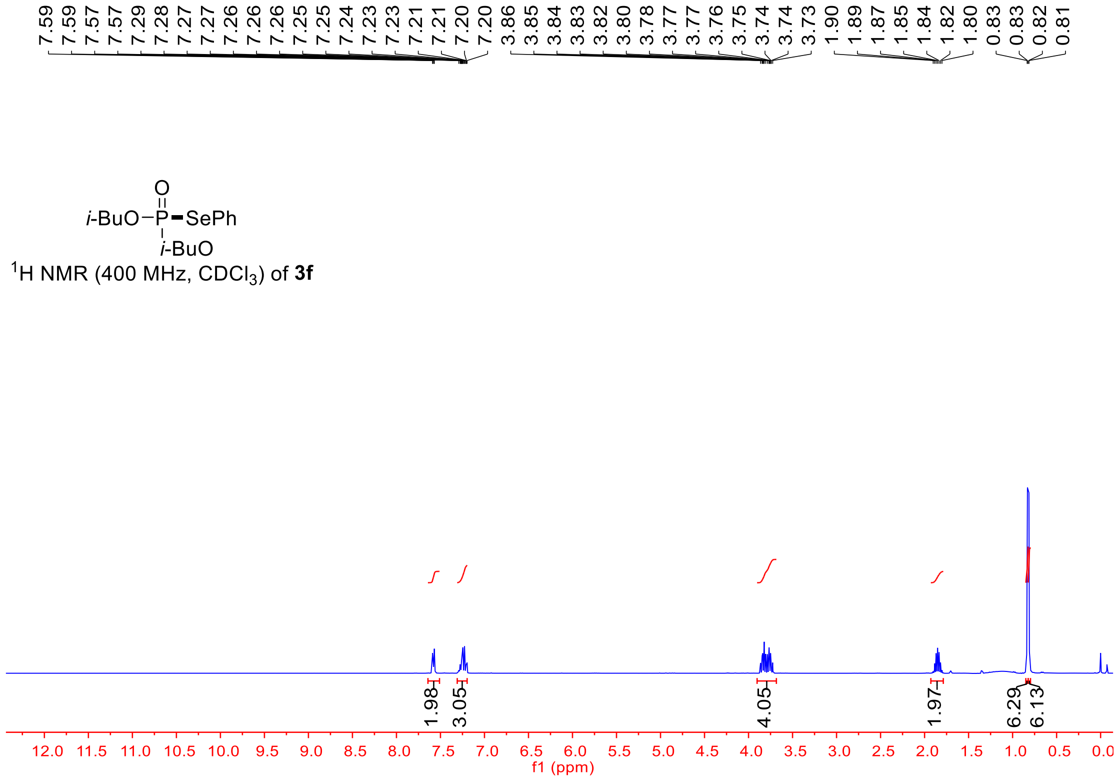

Figure S16: ^{1}H NMR spectrum for compound **3f**

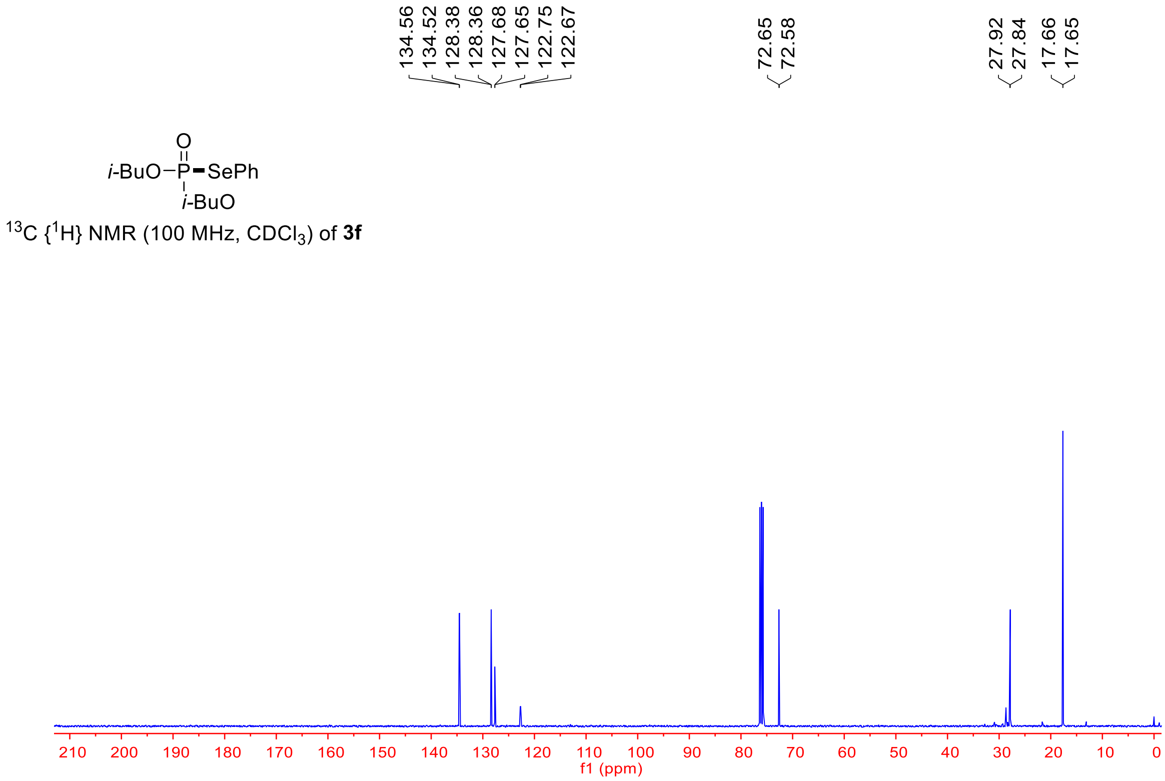

Figure S17: ^{13}C NMR spectrum for compound **3f**

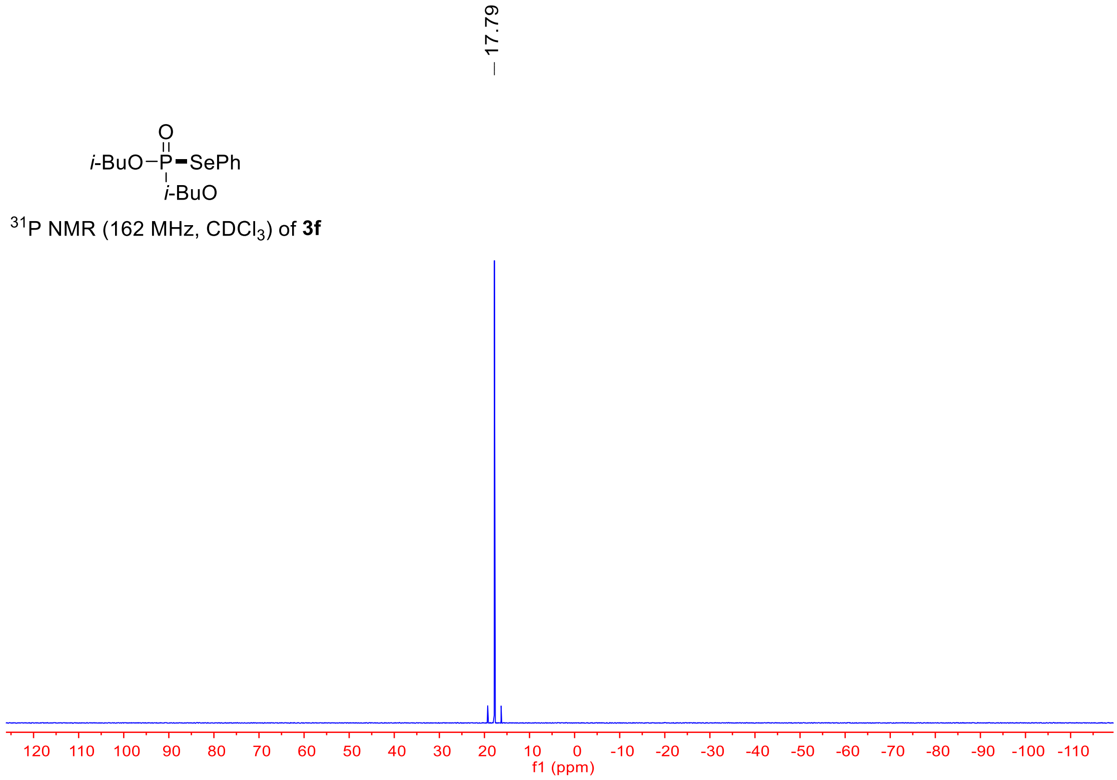

Figure S18: ^{31}P NMR spectrum for compound **3f**

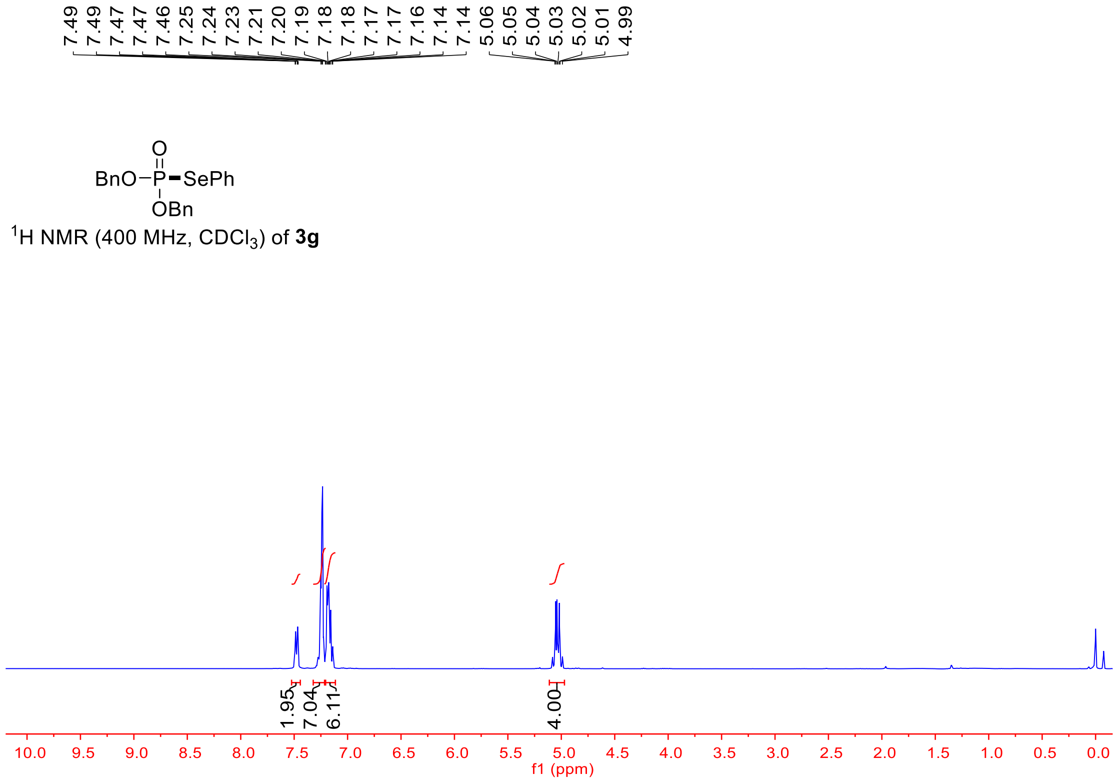

Figure S19: ^{1}H NMR spectrum for compound **3g**

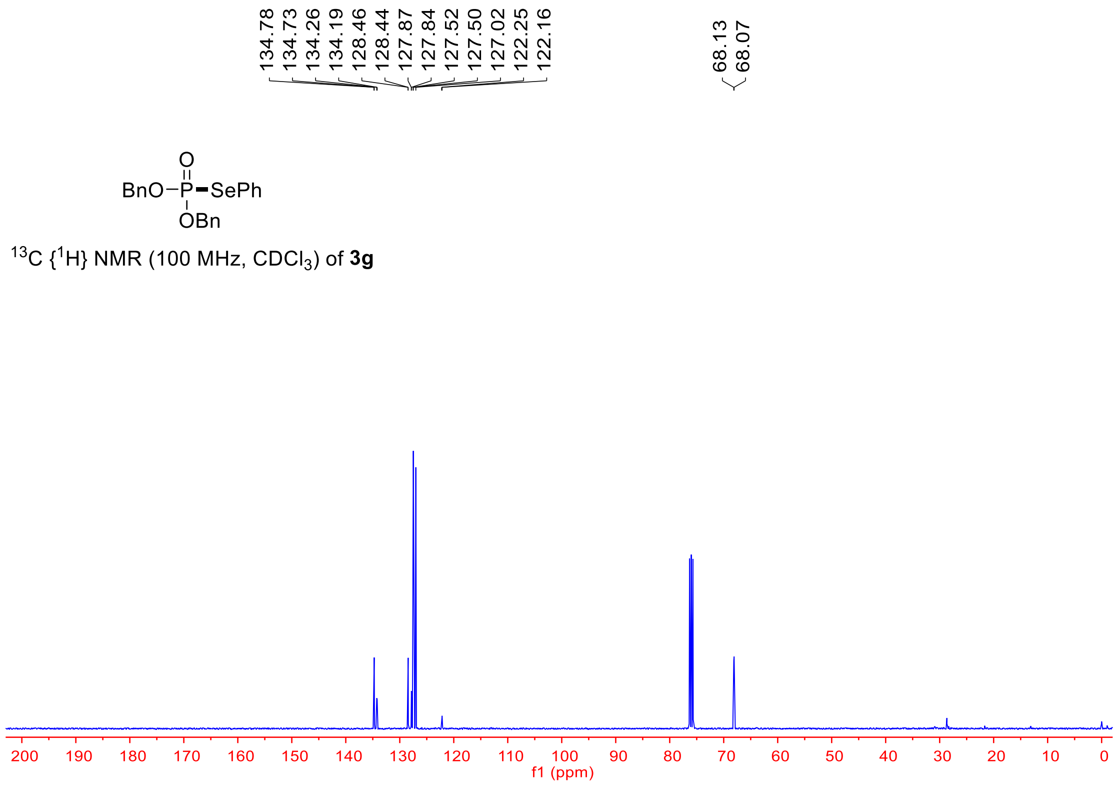

Figure S20: ^{13}C NMR spectrum for compound **3g**

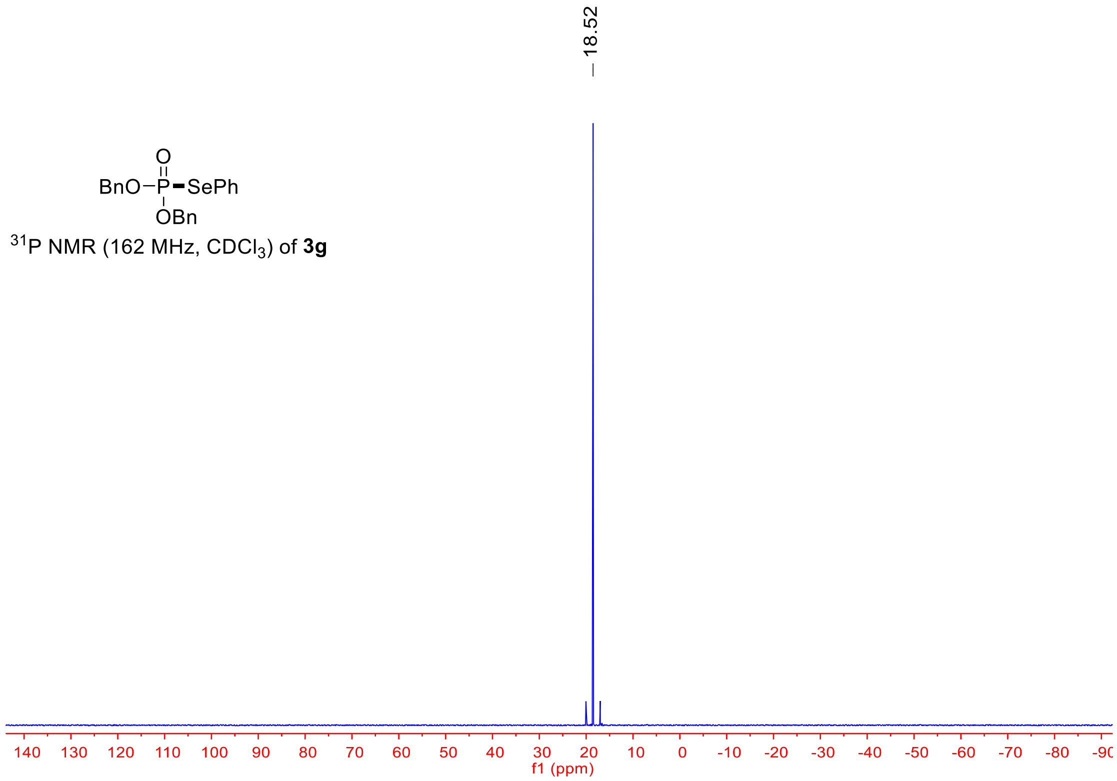

Figure S21: ^{31}P NMR spectrum for compound **3g**

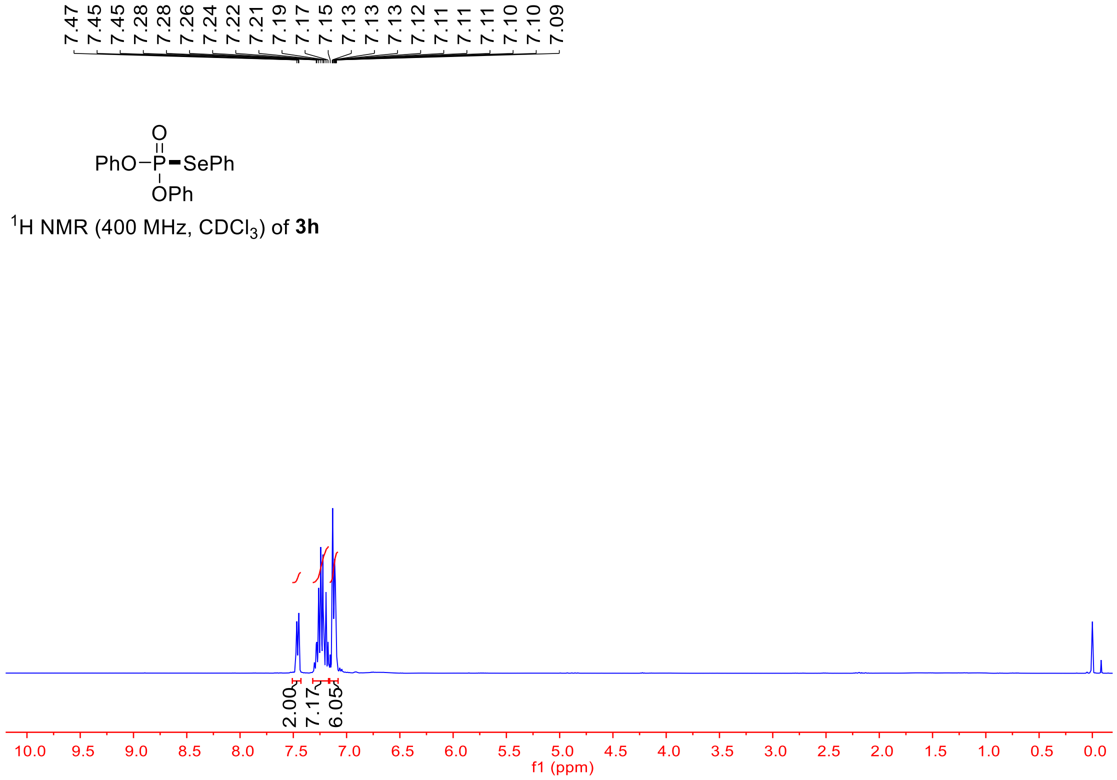

Figure S22: ^{1}H NMR spectrum for compound **3h**

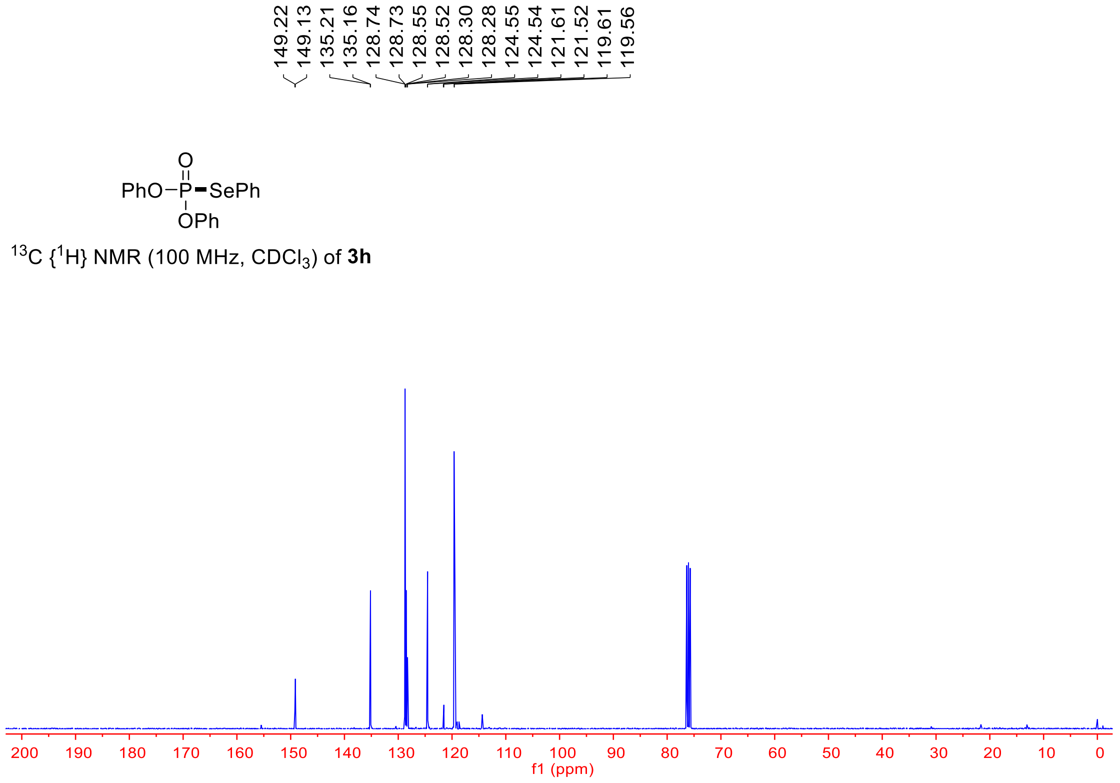

Figure S23: ^{13}C NMR spectrum for compound **3h**

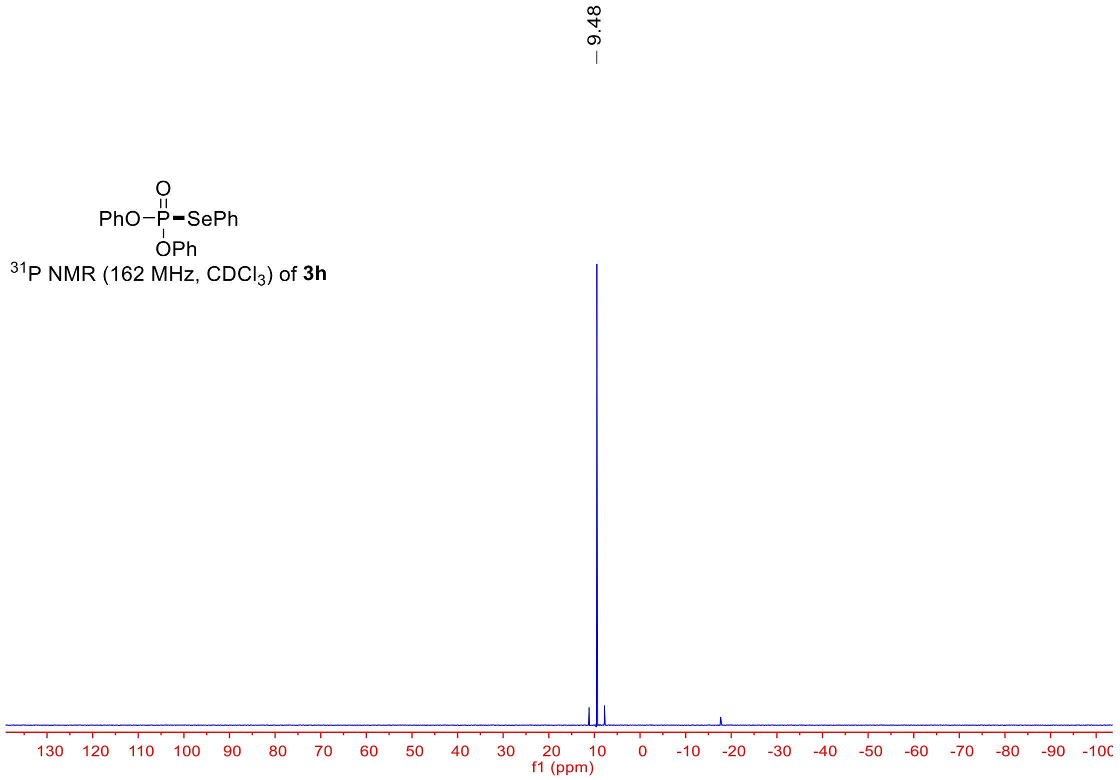

Figure S24: ^{31}P NMR spectrum for compound **3h**

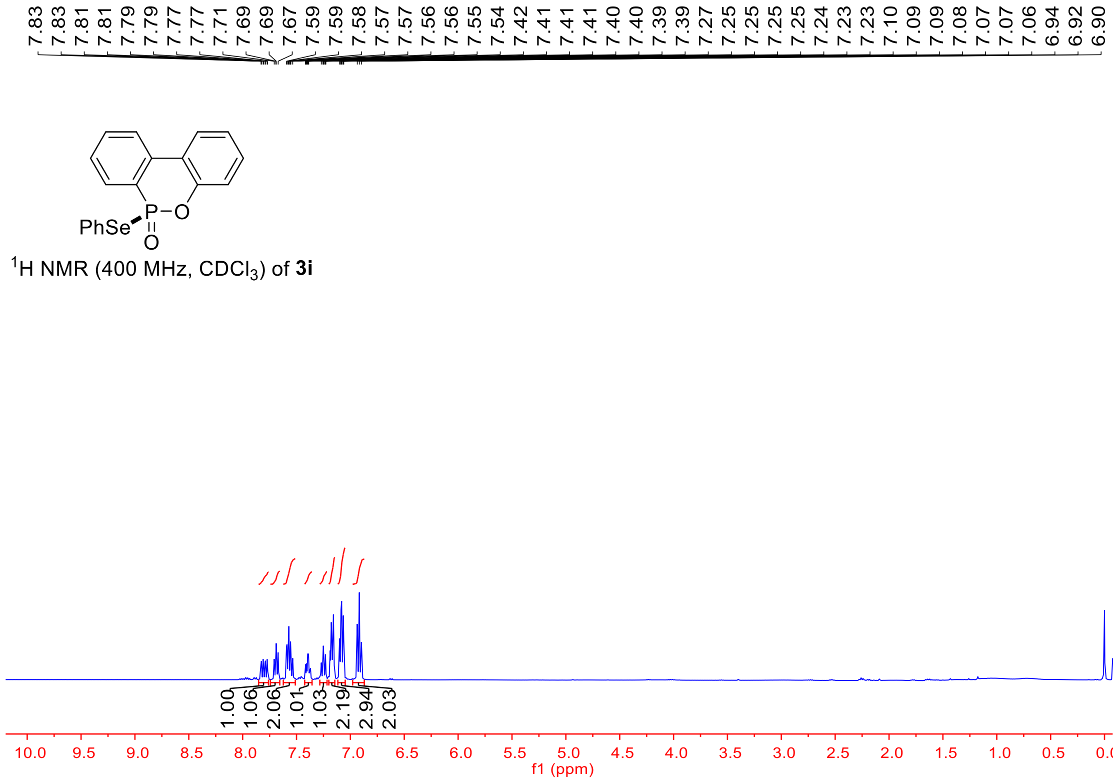

Figure S25: ^{1}H NMR spectrum for compound **3i**

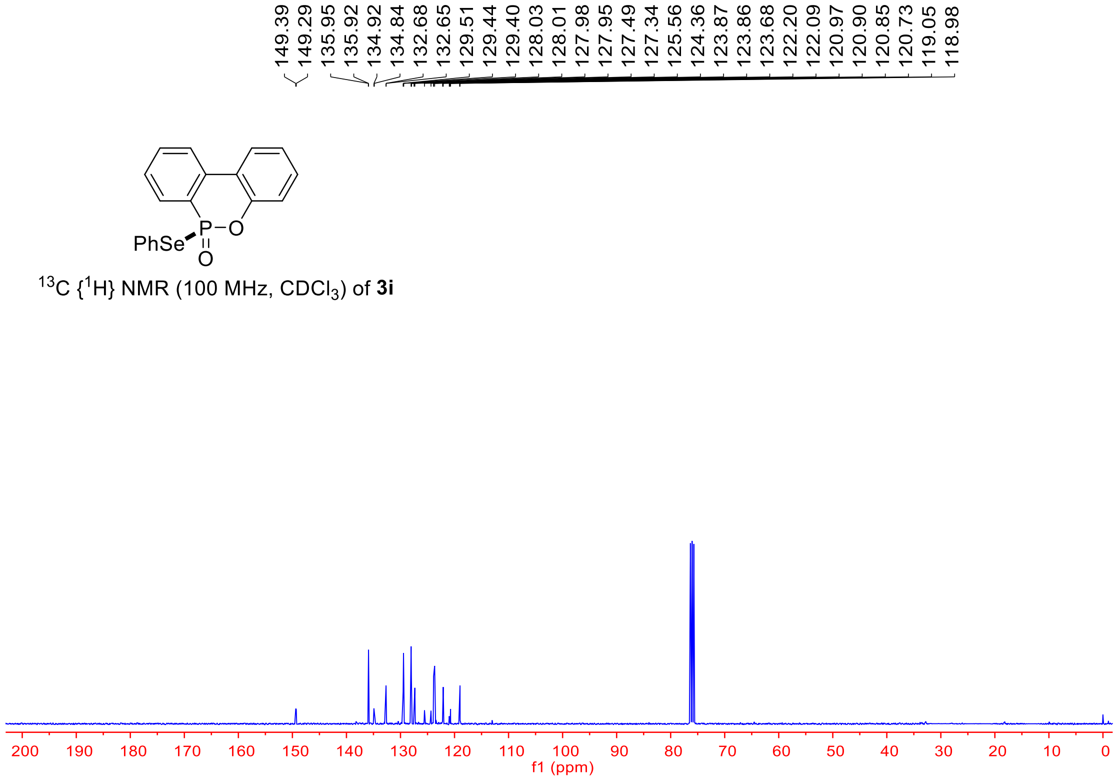

Figure S26: ^{13}C NMR spectrum for compound **3i**

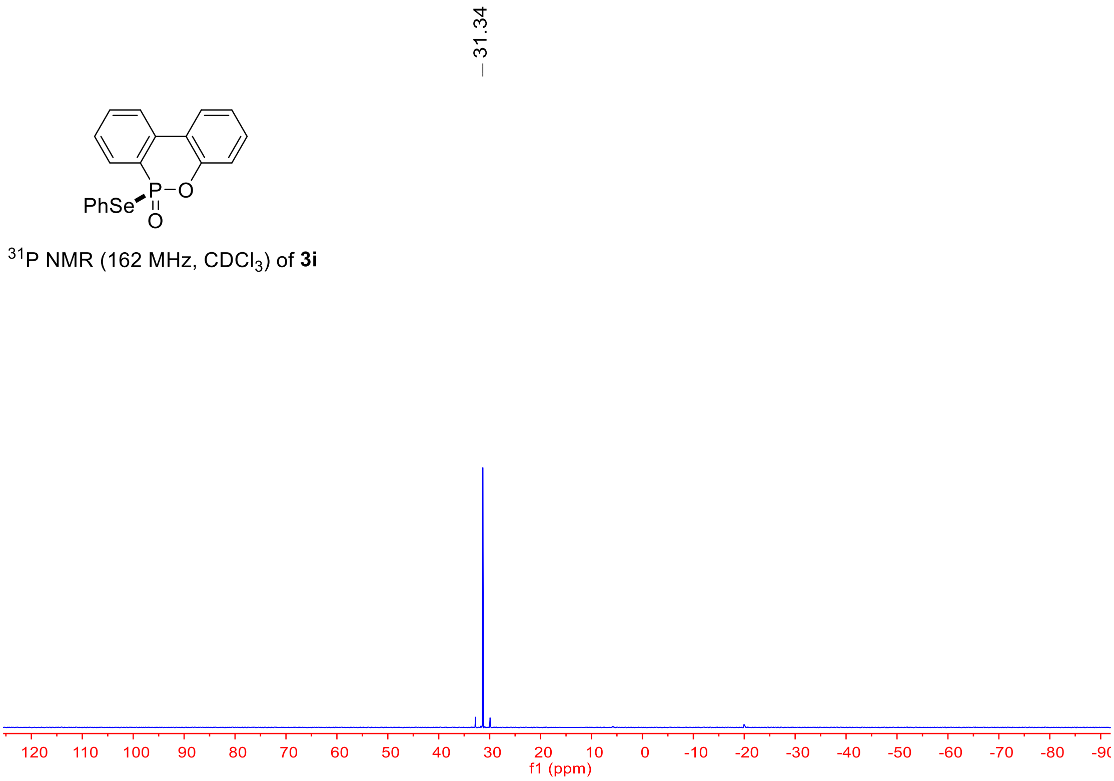

Figure S27: ^{31}P NMR spectrum for compound **3i**

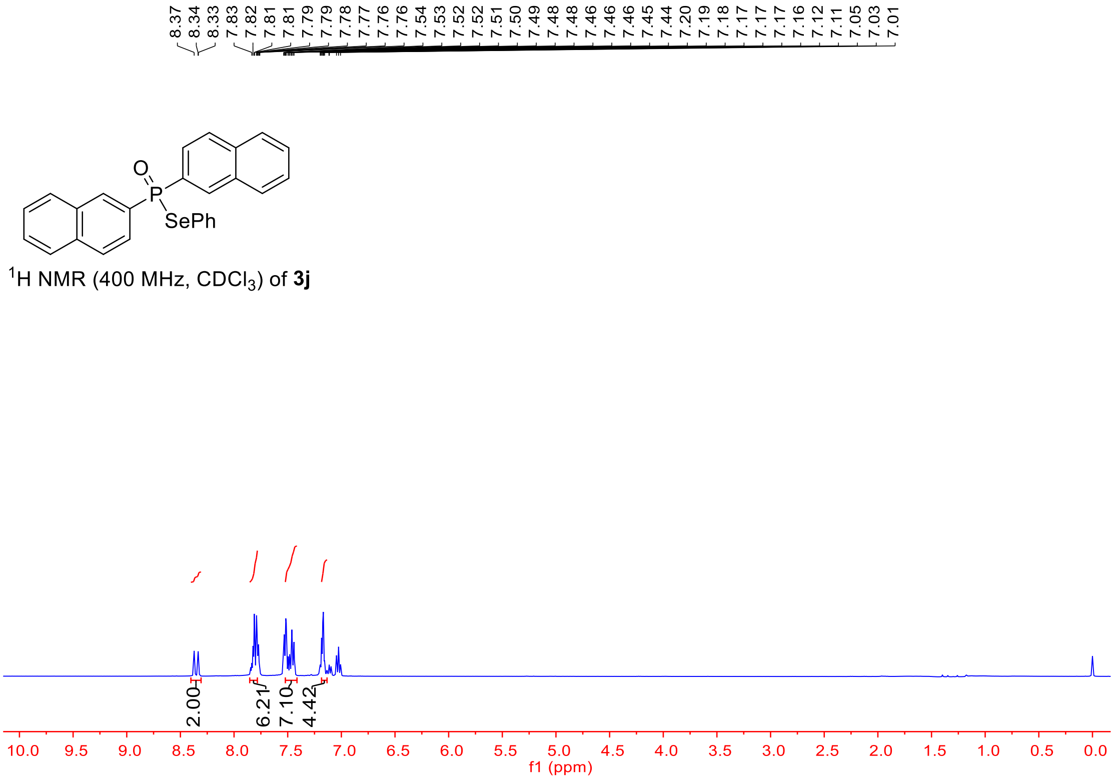

Figure S28: ^{1}H NMR spectrum for compound **3j**

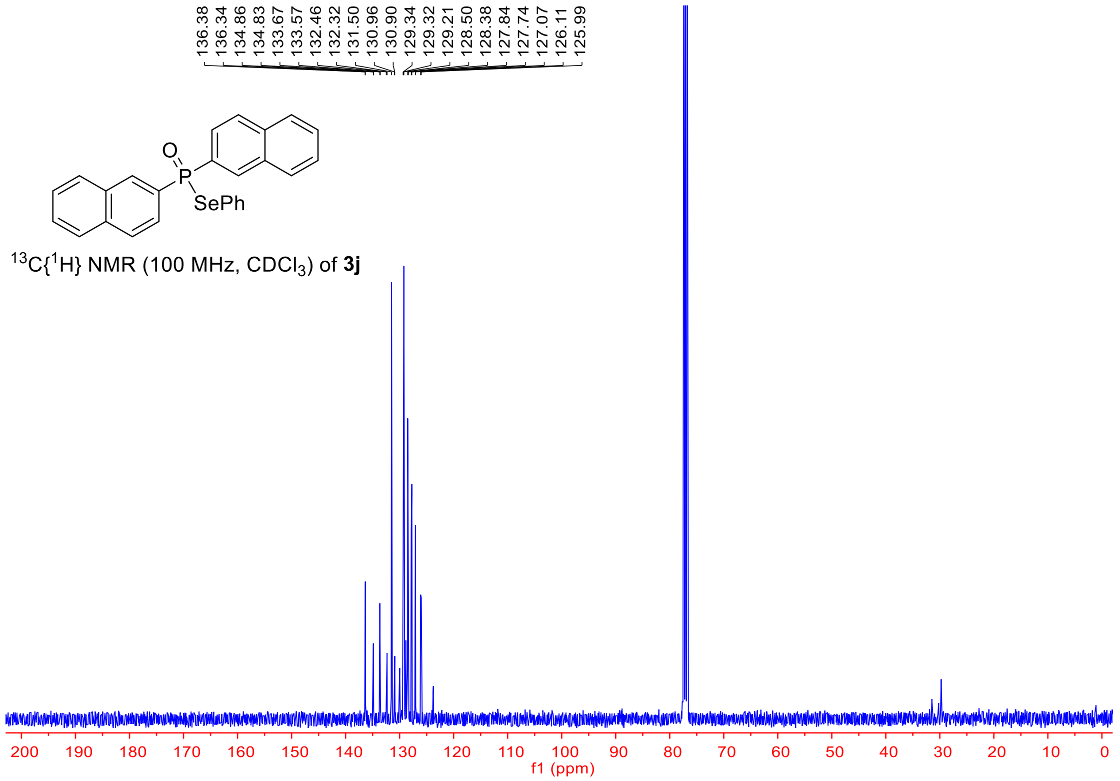

Figure S29: ^{13}C NMR spectrum for compound **3j**

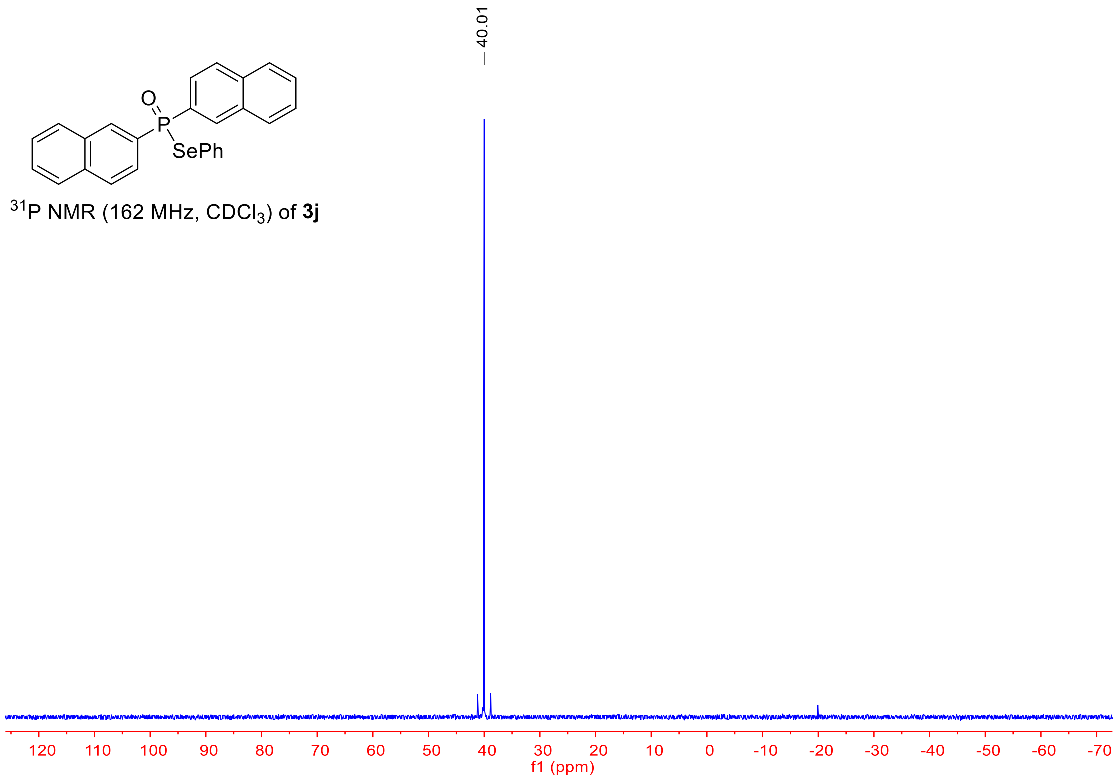

Figure S30: ^{31}P NMR spectrum for compound **3j**

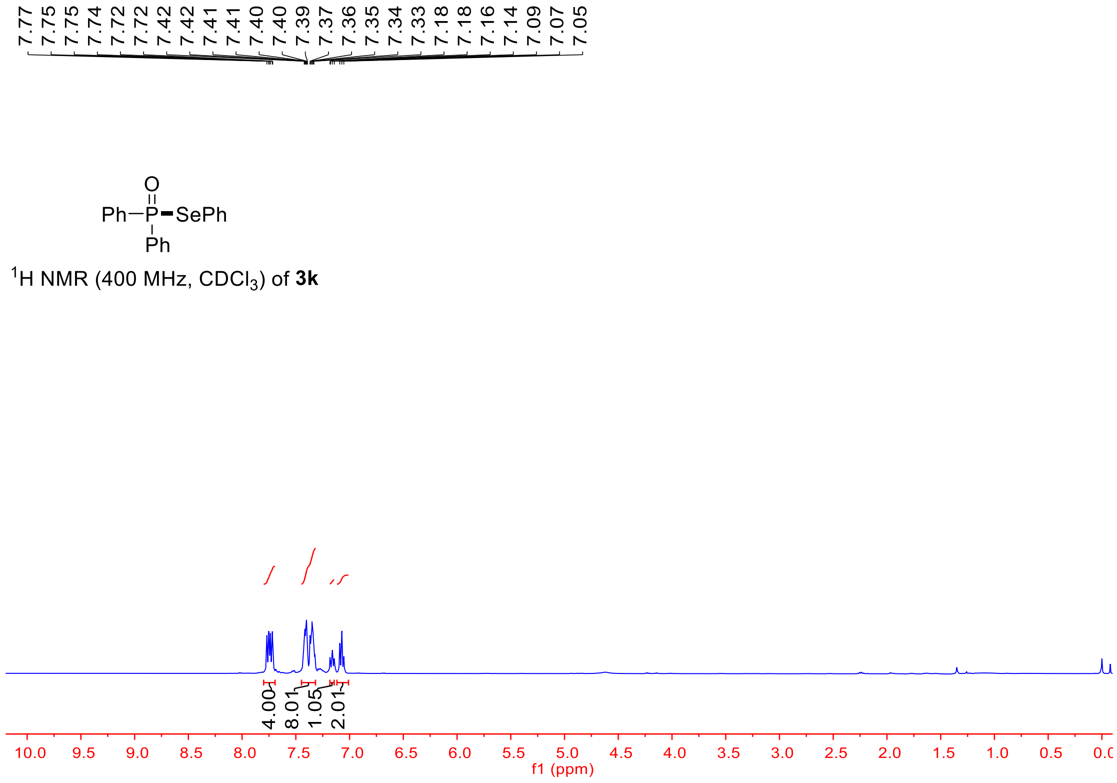

Figure S31: ^{1}H NMR spectrum for compound **3k**

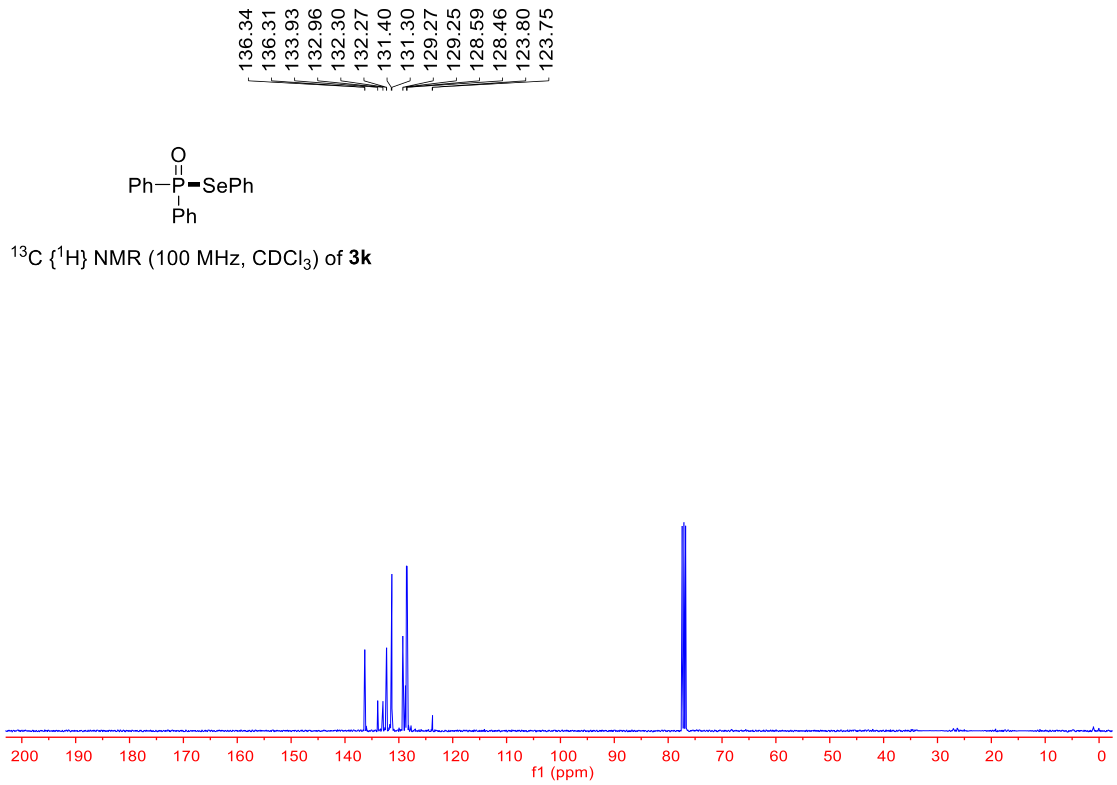

Figure S32: ^{13}C NMR spectrum for compound **3k**

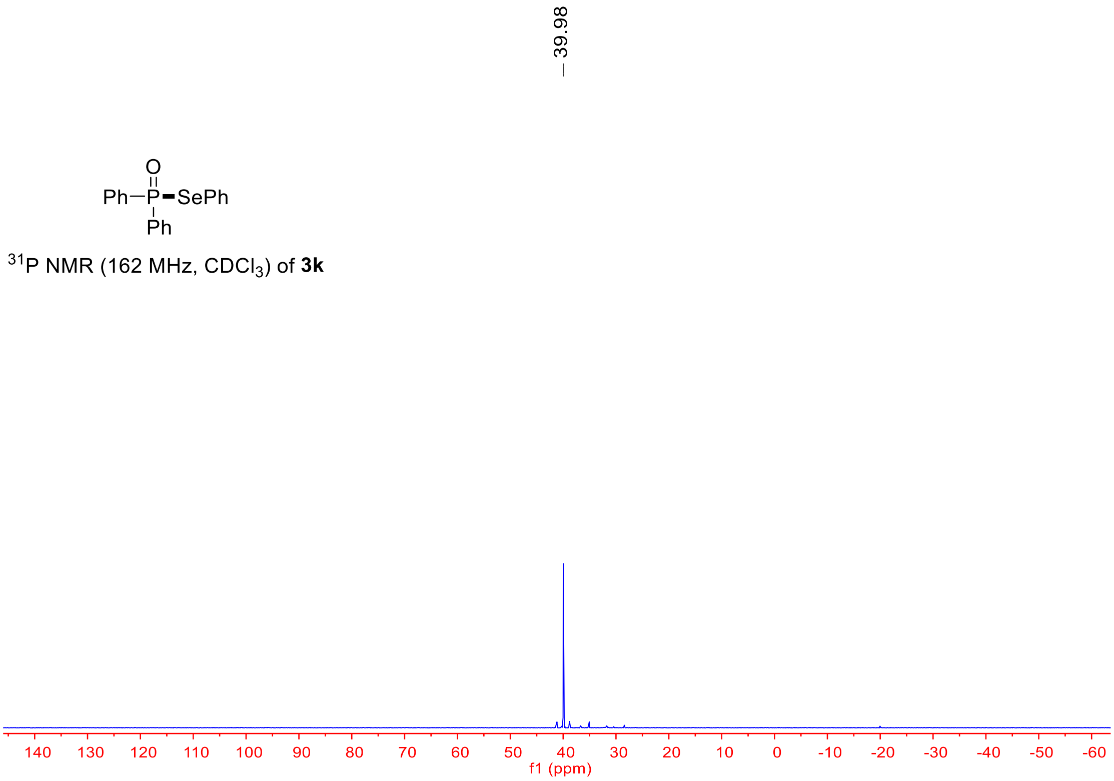

Figure S33: ^{31}P NMR spectrum for compound **3k**

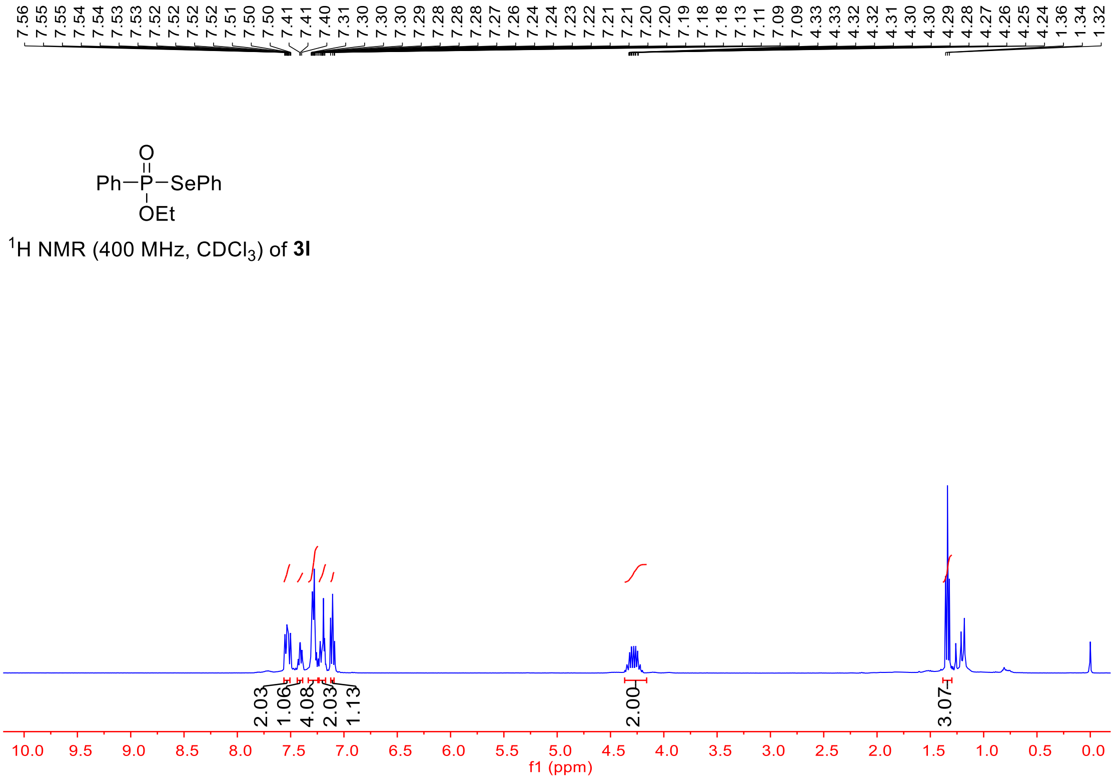

Figure S34: ^{1}H NMR spectrum for compound **3l**

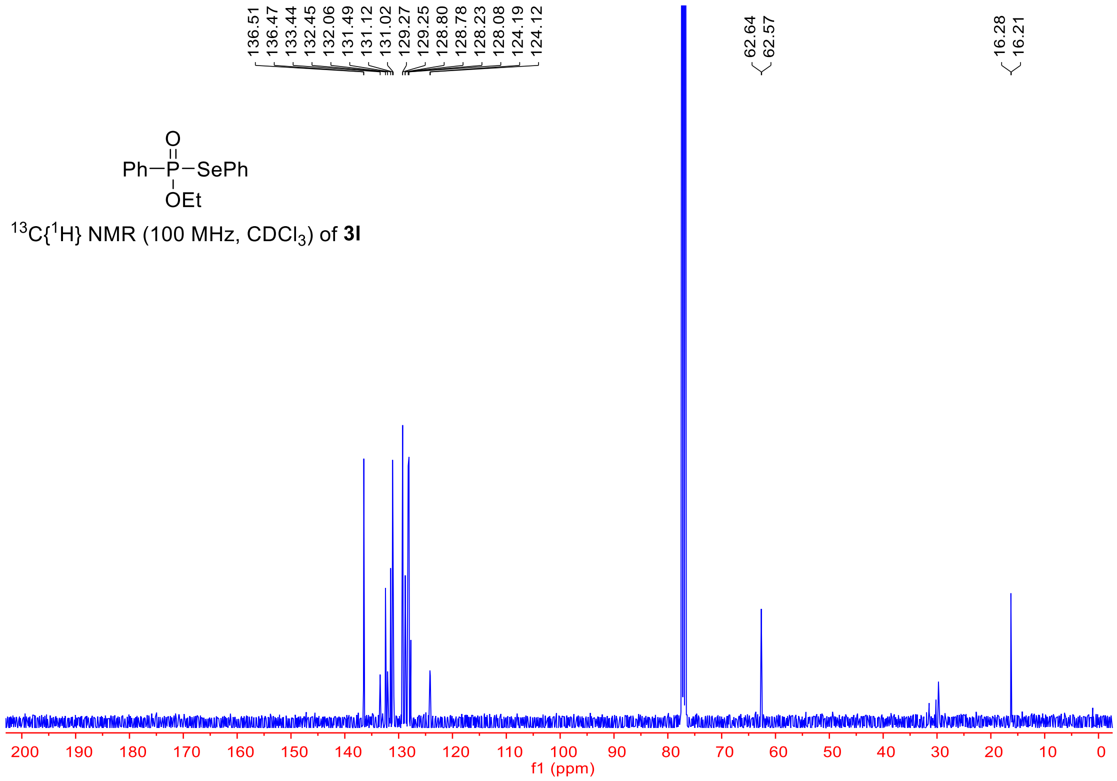

Figure S35: ^{13}C NMR spectrum for compound **3l**

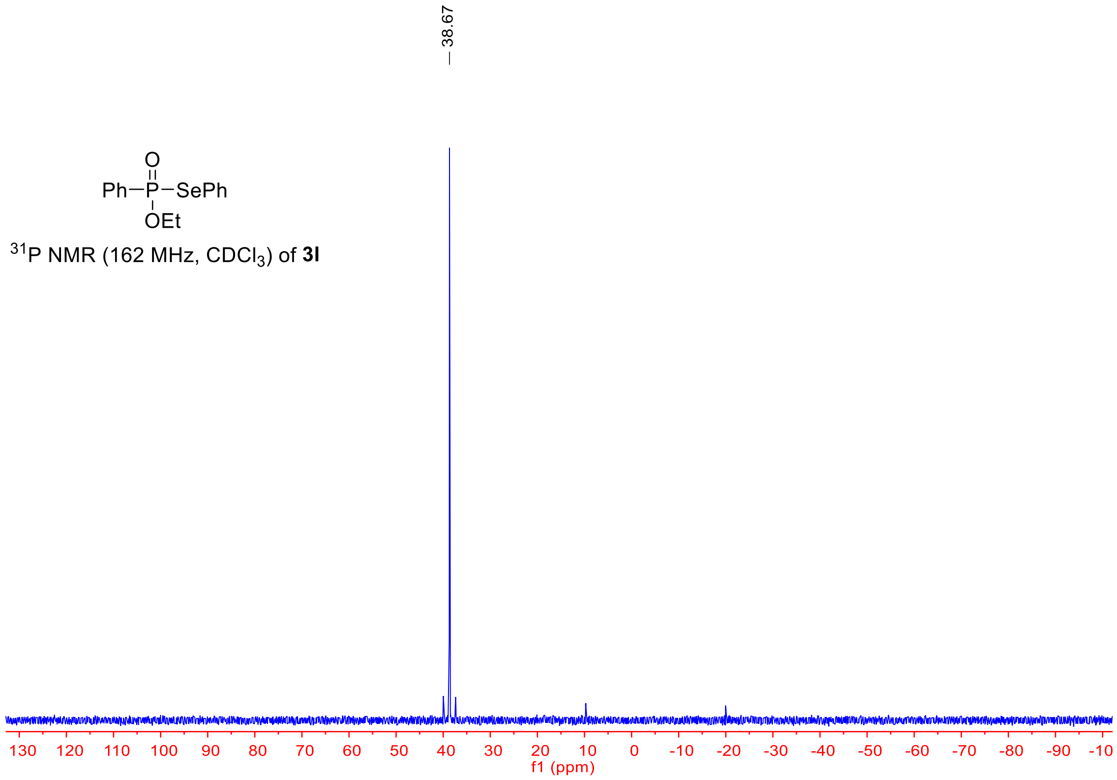

Figure S36: ^{31}P NMR spectrum for compound **3l**

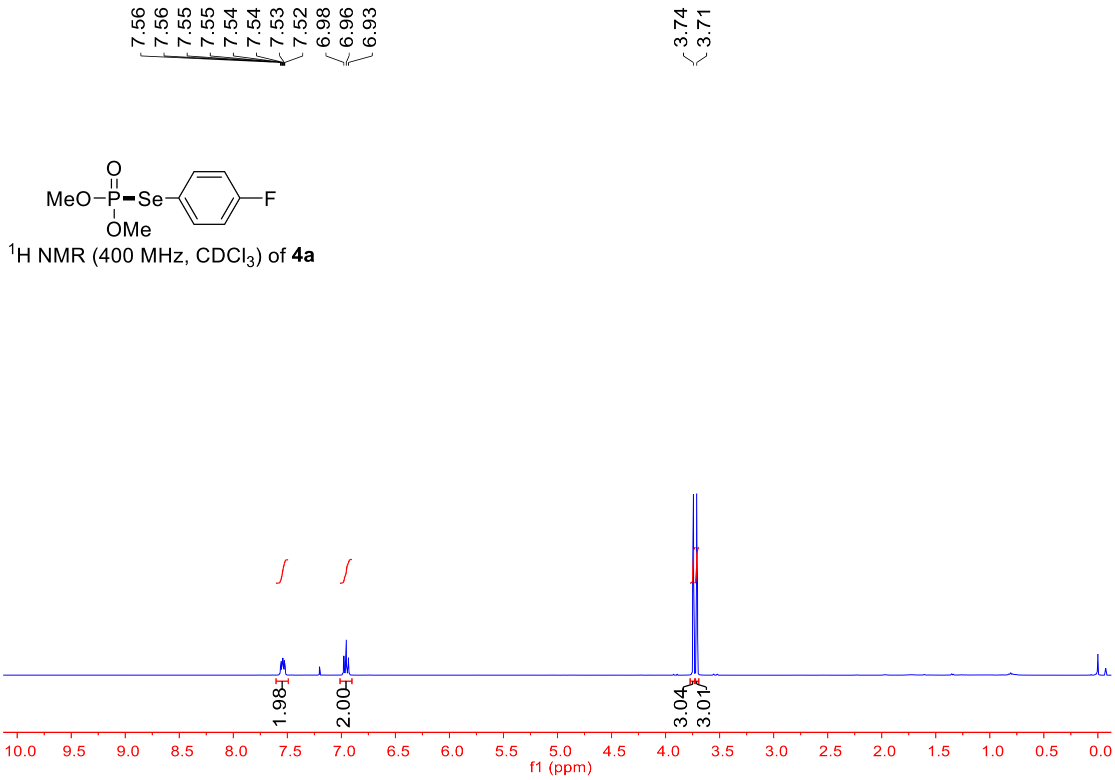

Figure S37: ^{1}H NMR spectrum for compound **4a**

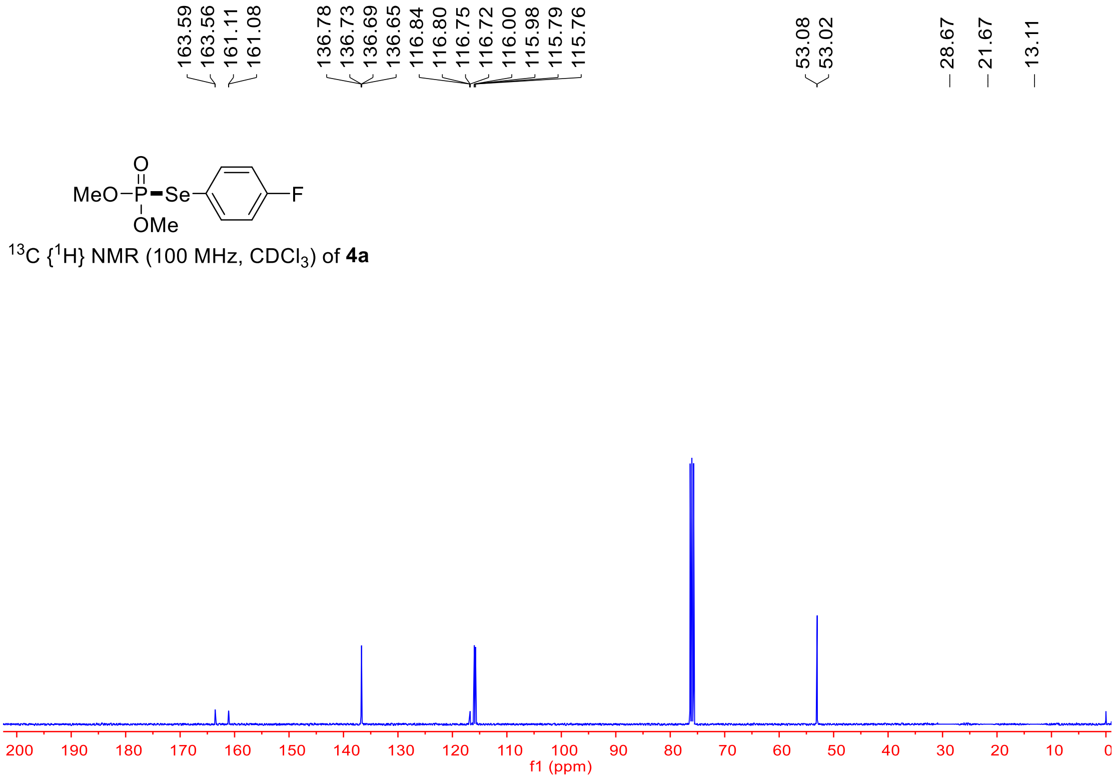

Figure S38: ^{13}C NMR spectrum for compound **4a**

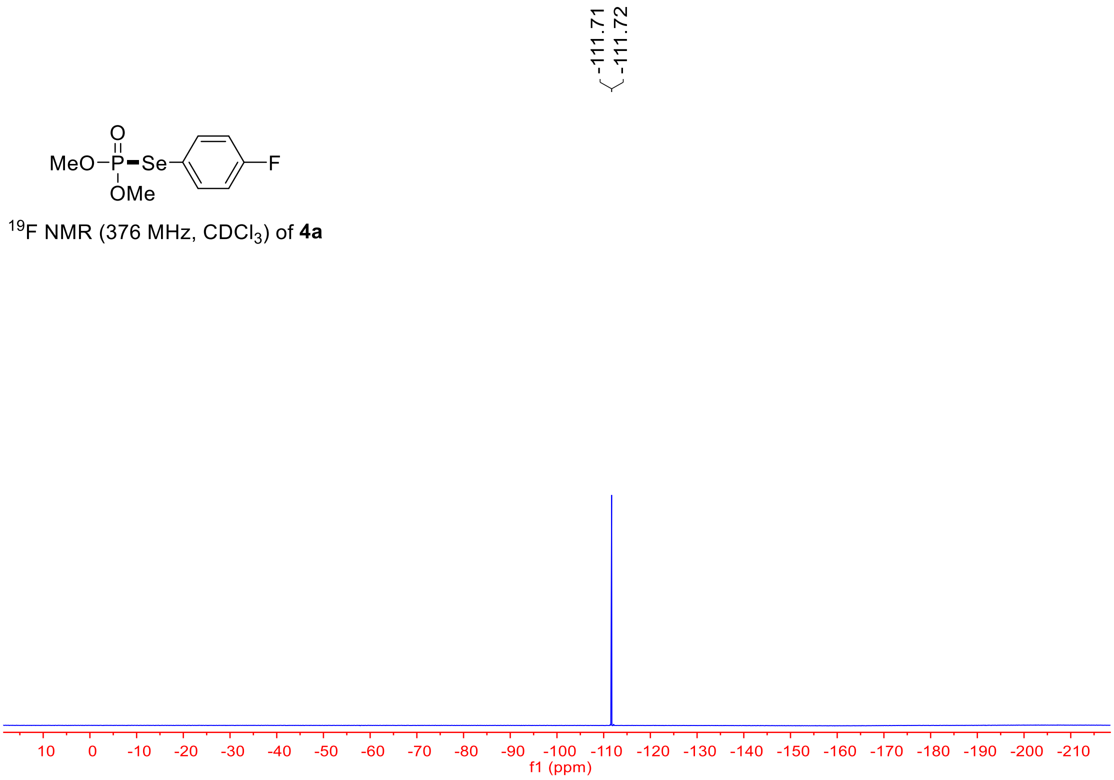

Figure S39: ^{19}F NMR spectrum for compound **4a**

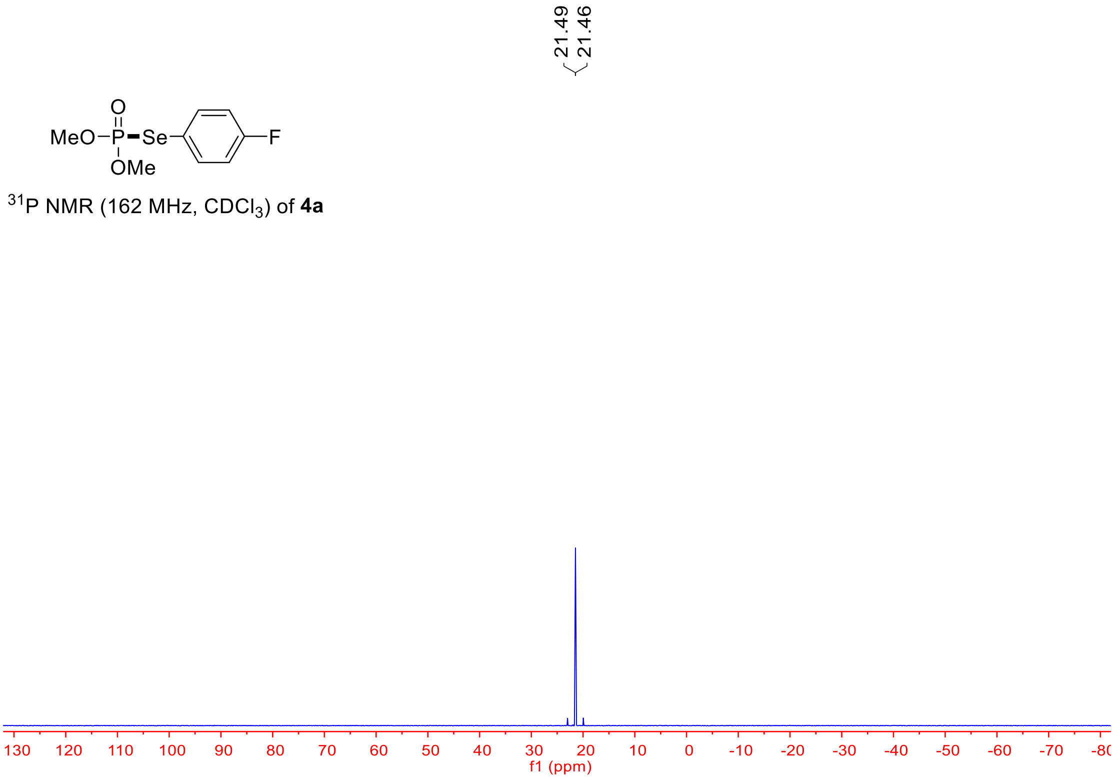

Figure S40: ^{31}P NMR spectrum for compound **4a**

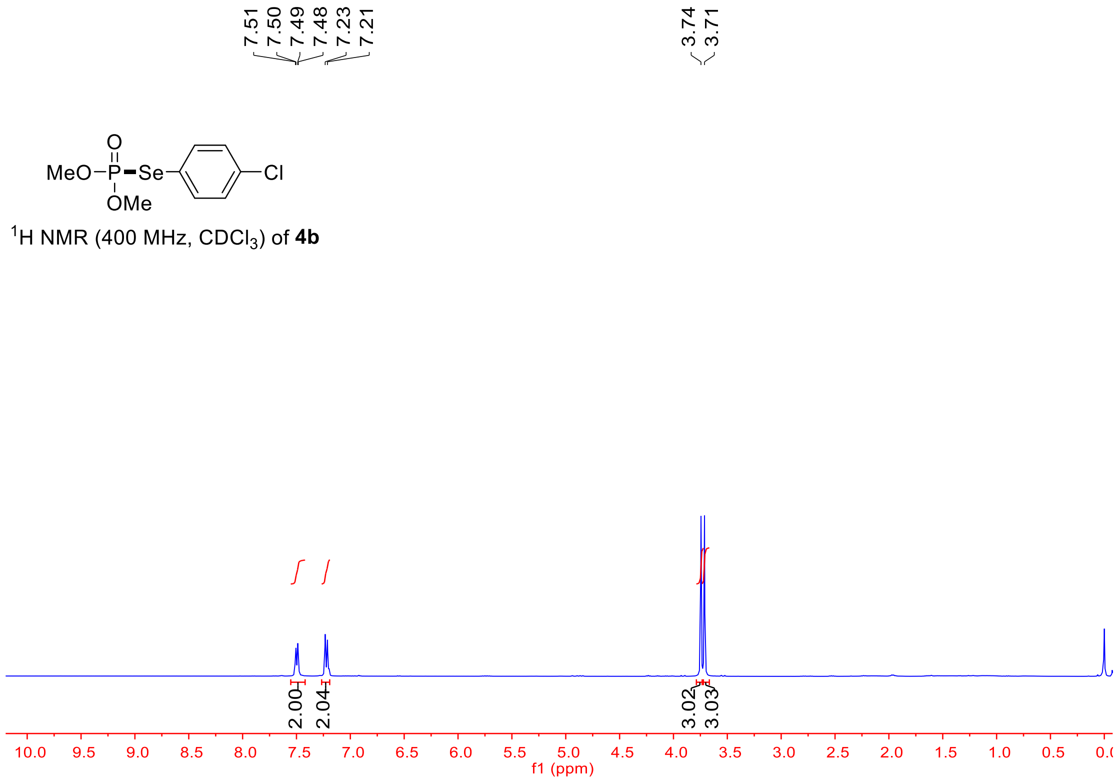

Figure S41: ^{1}H NMR spectrum for compound **4b**

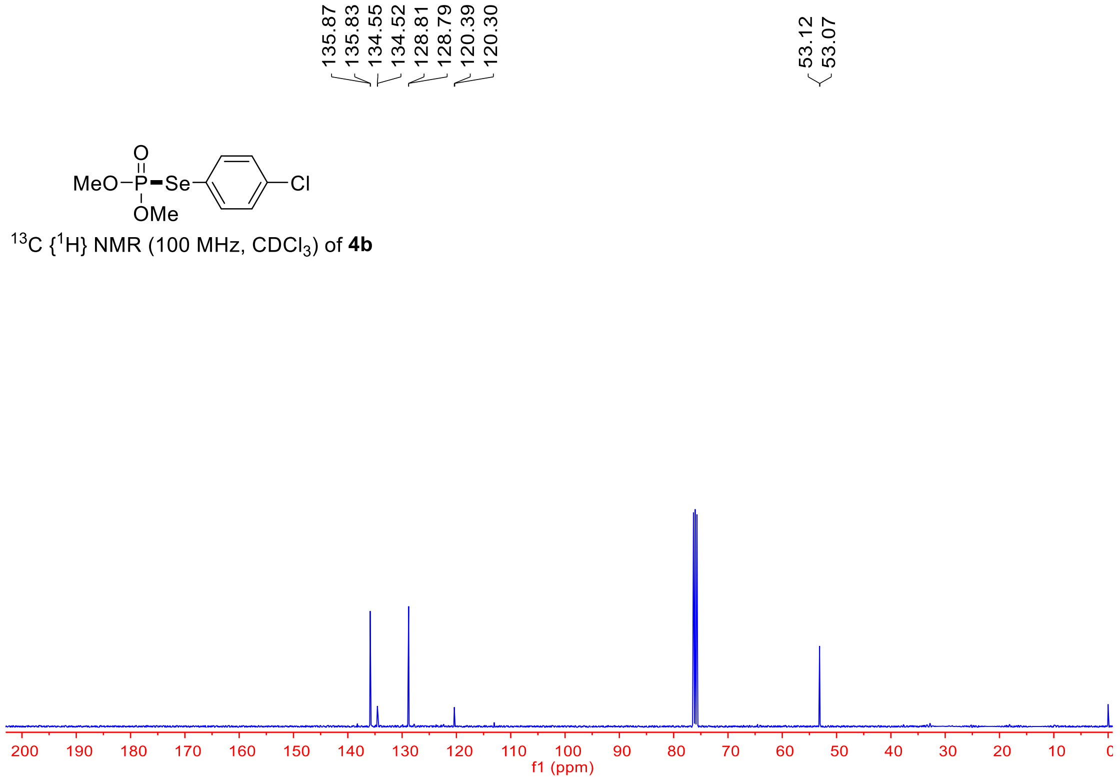

Figure S42: ^{13}C NMR spectrum for compound **4b**

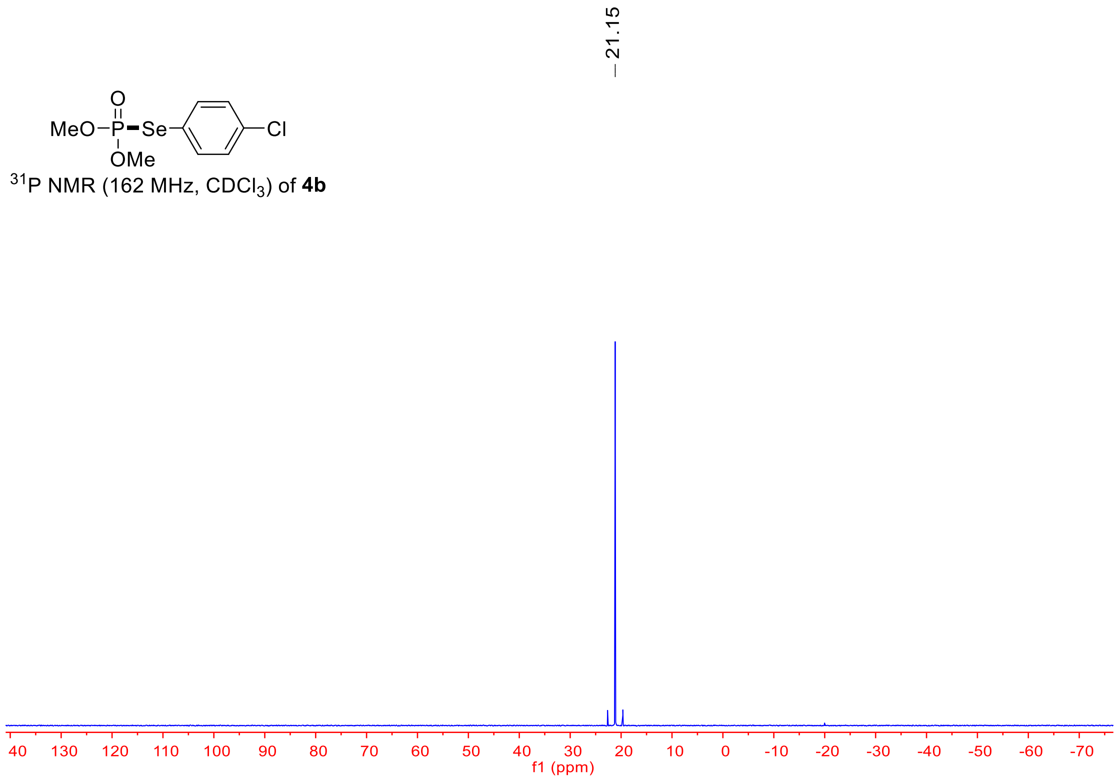

Figure S43: ^{31}P NMR spectrum for compound **4b**

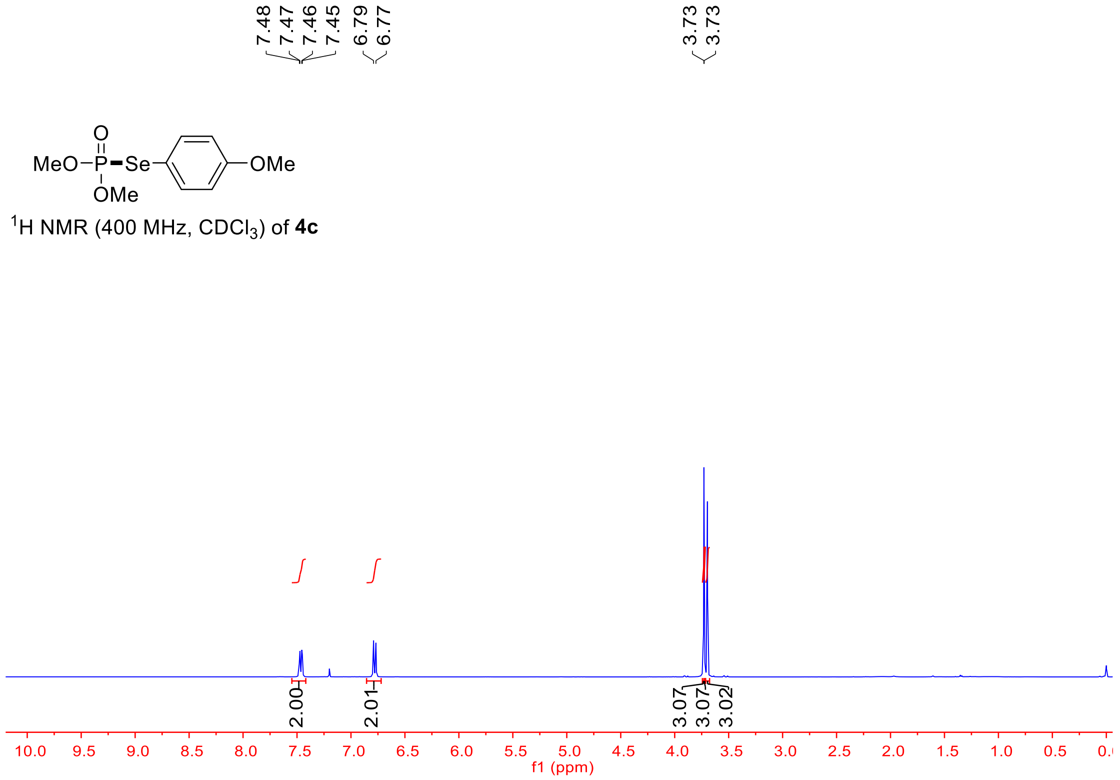

Figure S44: ^{1}H NMR spectrum for compound **4c**

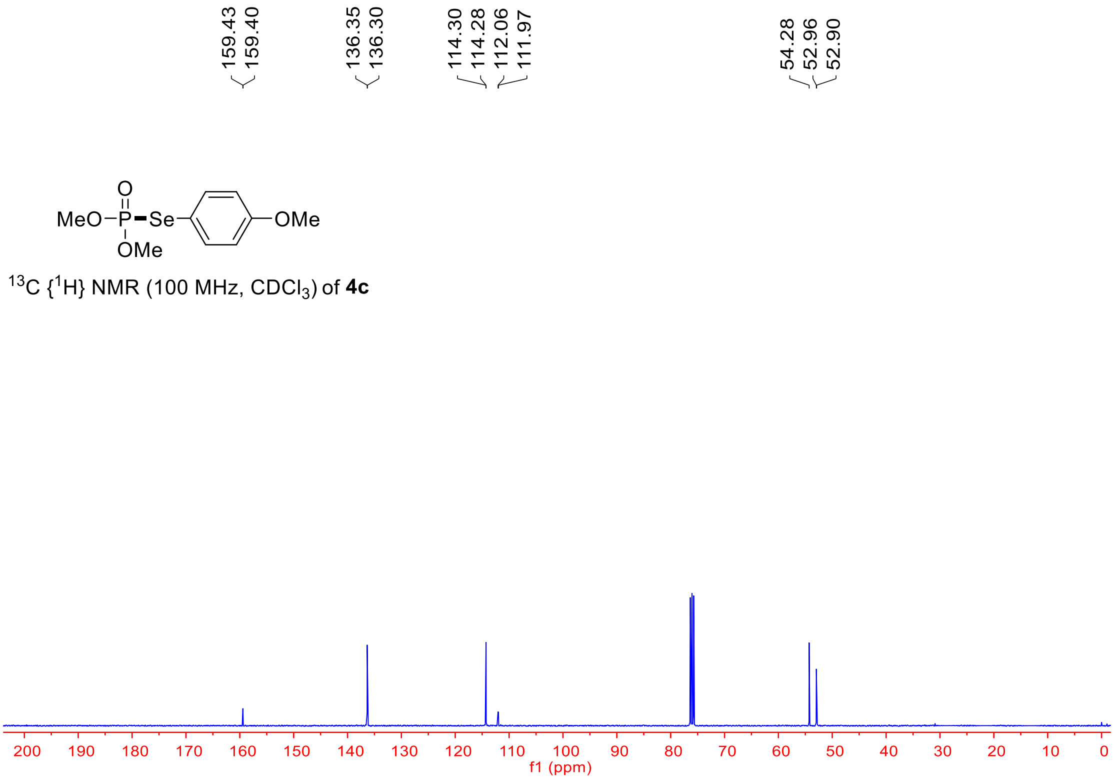

Figure S45: ^{13}C NMR spectrum for compound **4c**

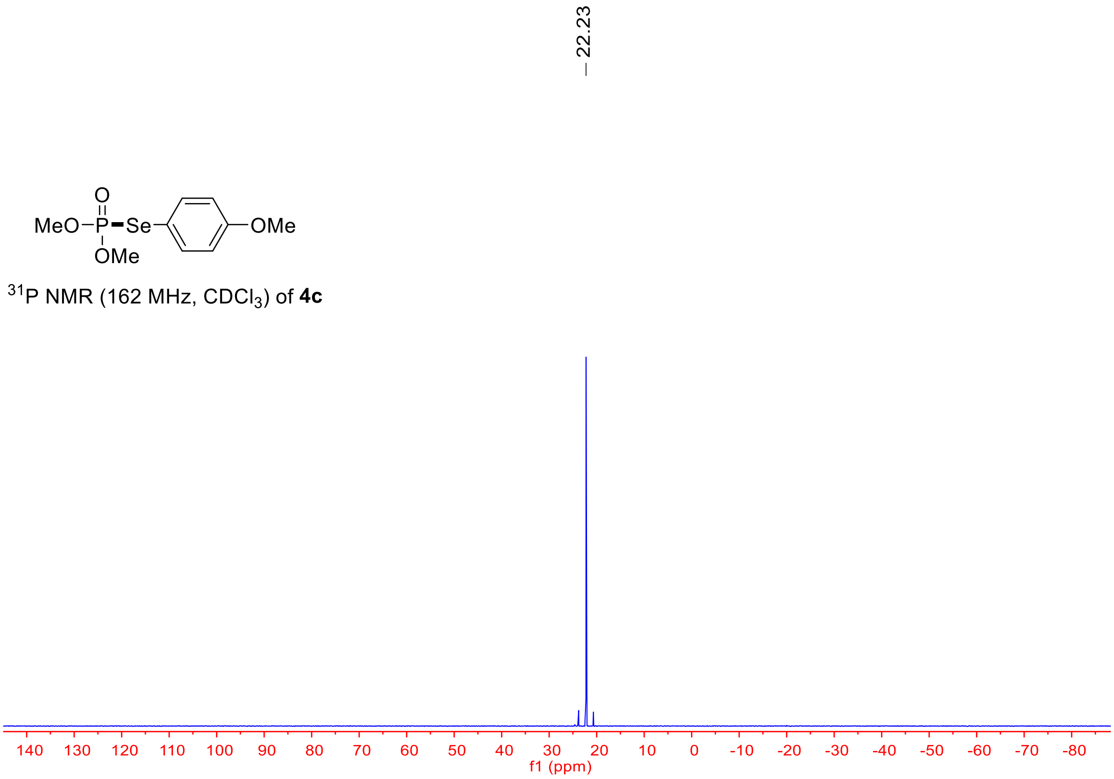

Figure S46: ^{31}P NMR spectrum for compound **4c**

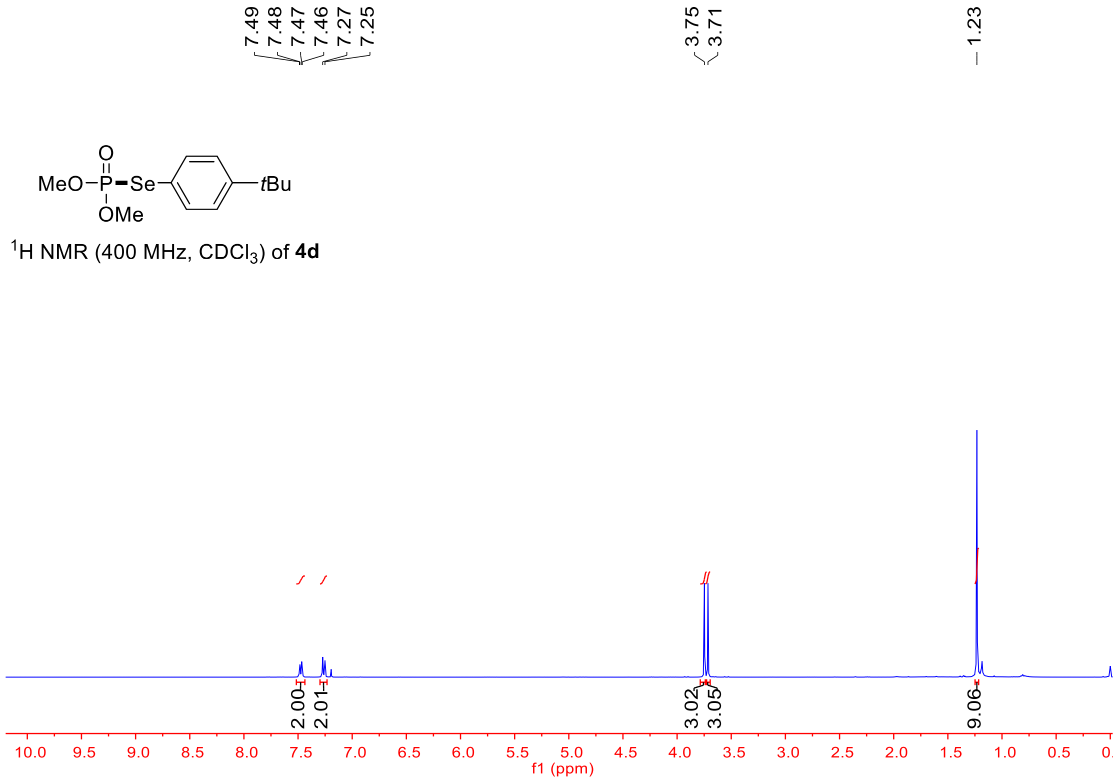

Figure S47: ^{1}H NMR spectrum for compound **4d**

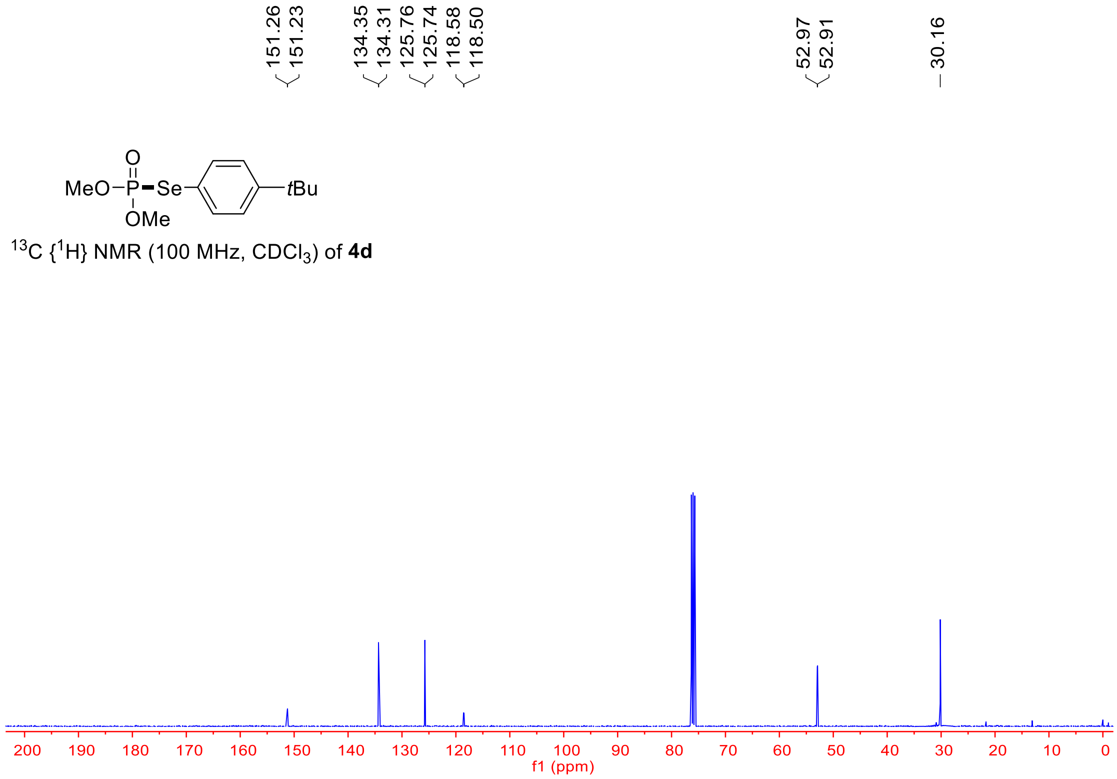

Figure S48: ^{13}C NMR spectrum for compound **4d**

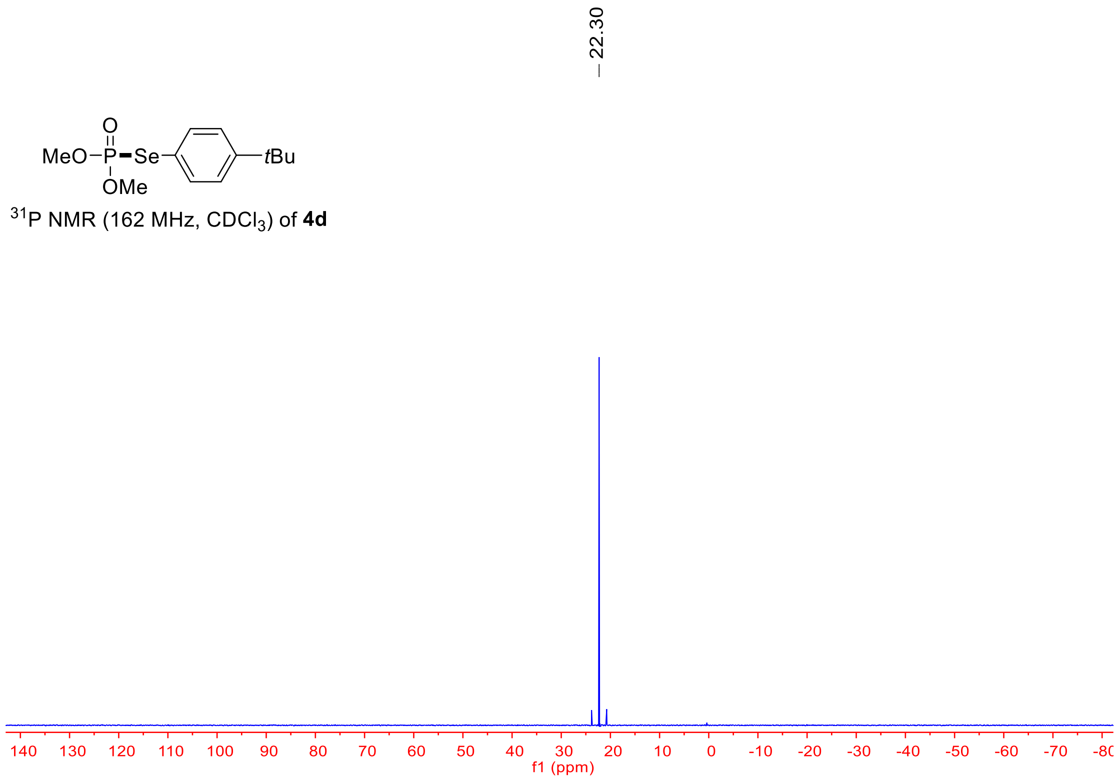

Figure S49: ^{31}P NMR spectrum for compound **4d**

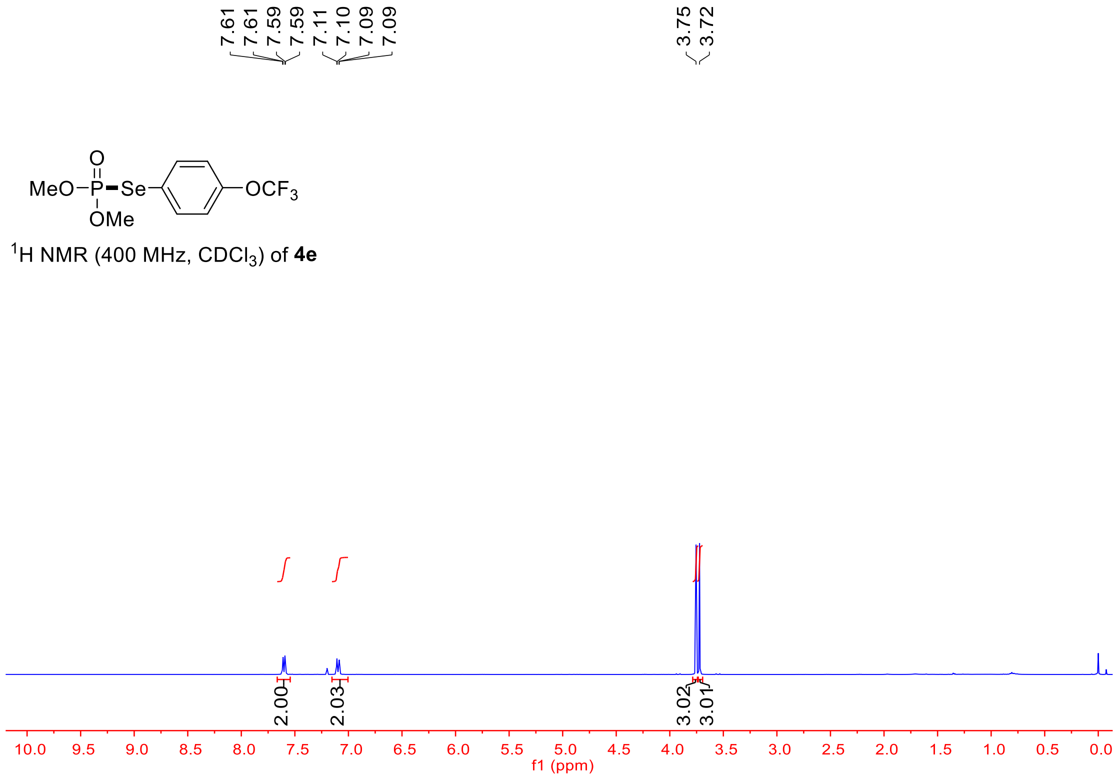

Figure S50: ^{1}H NMR spectrum for compound **4e**

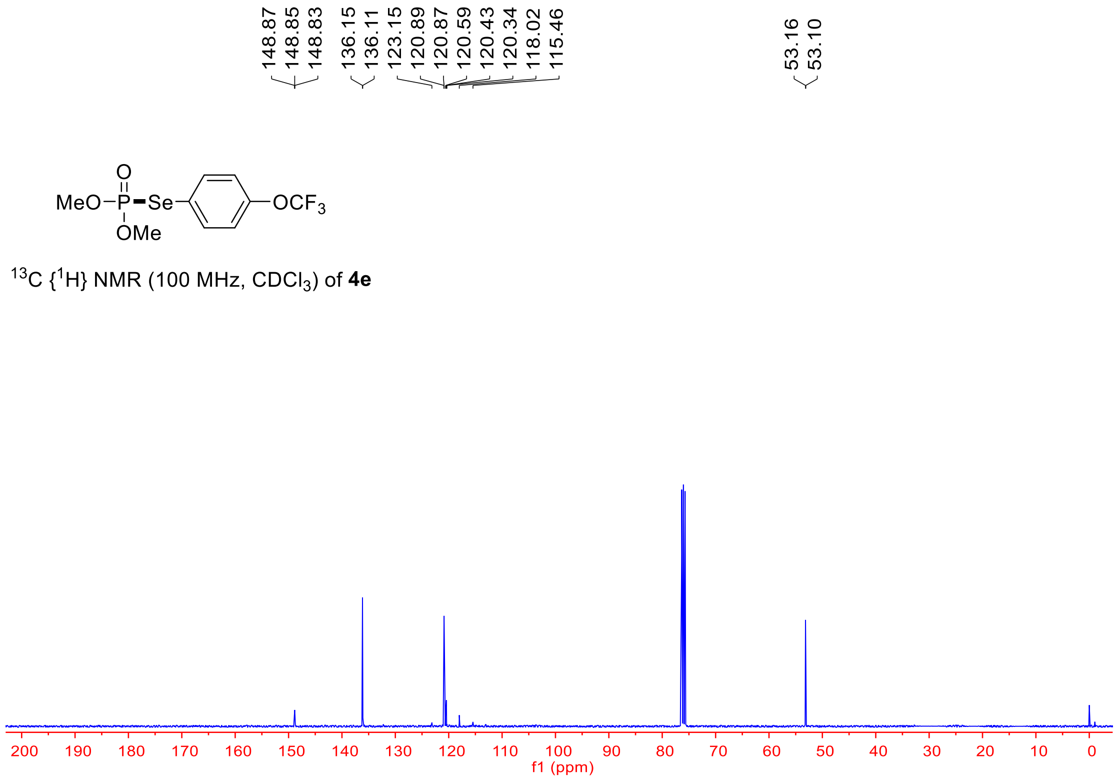

Figure S51: ^{13}C NMR spectrum for compound **4e**

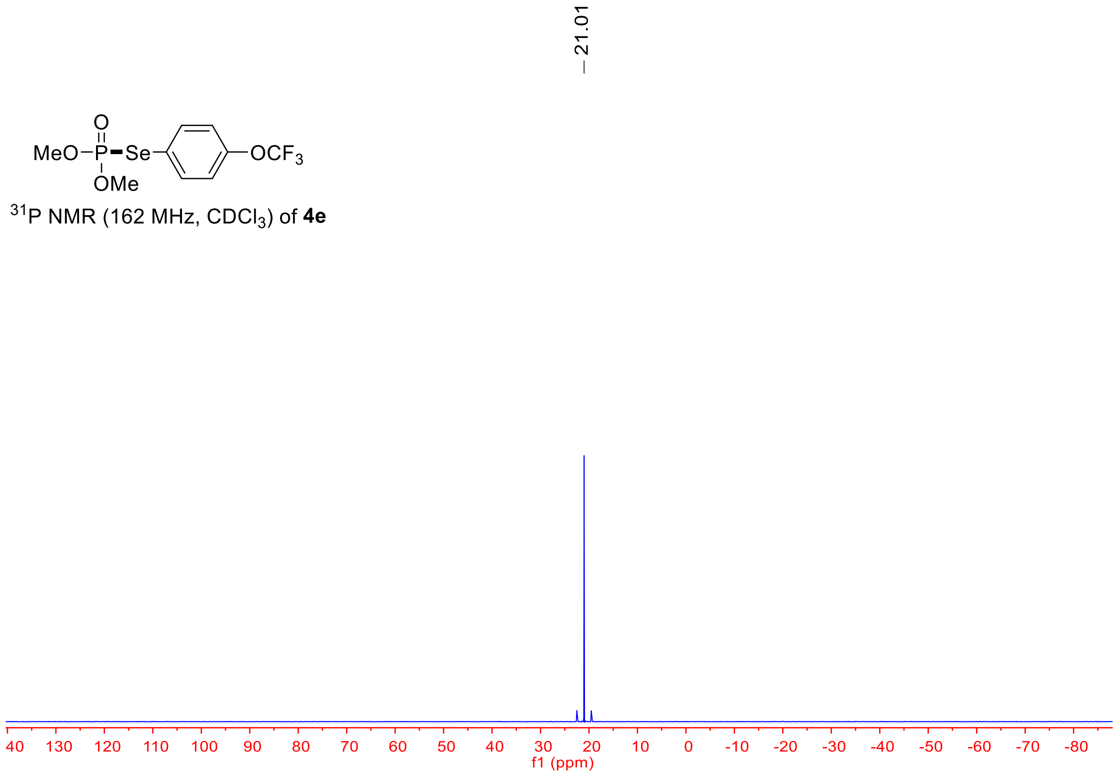

Figure S52: ^{31}P NMR spectrum for compound **4e**

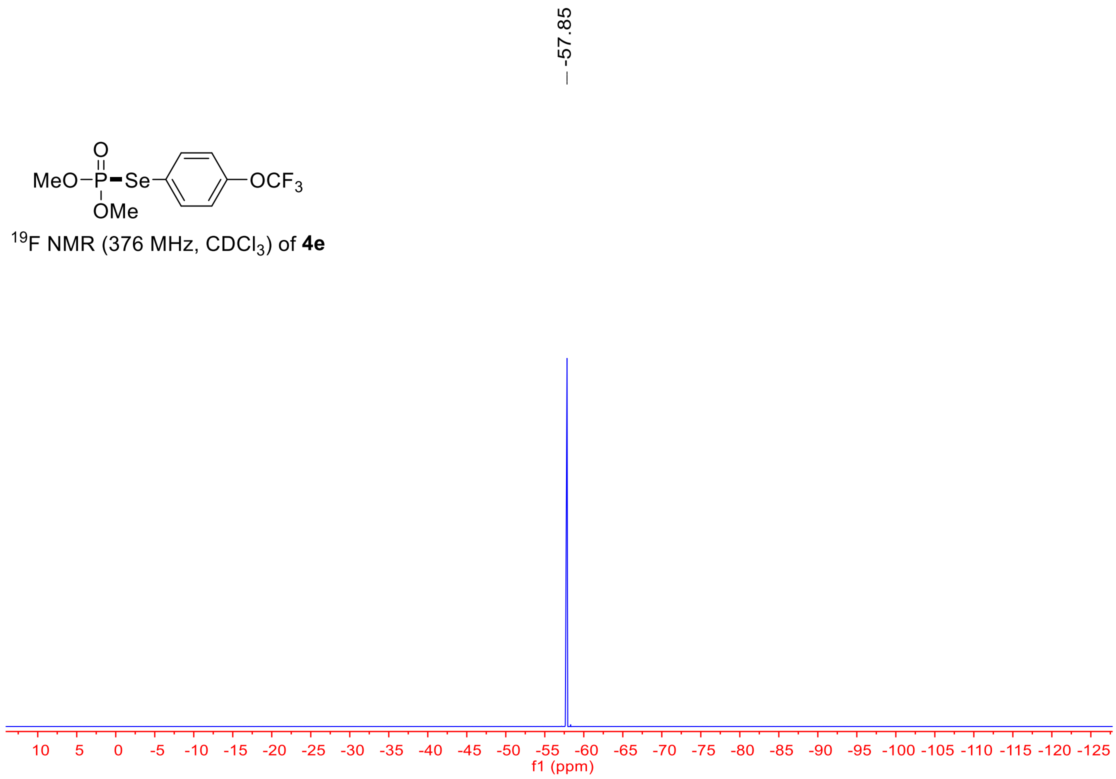

Figure S53: ^{19}F NMR spectrum for compound **4e**

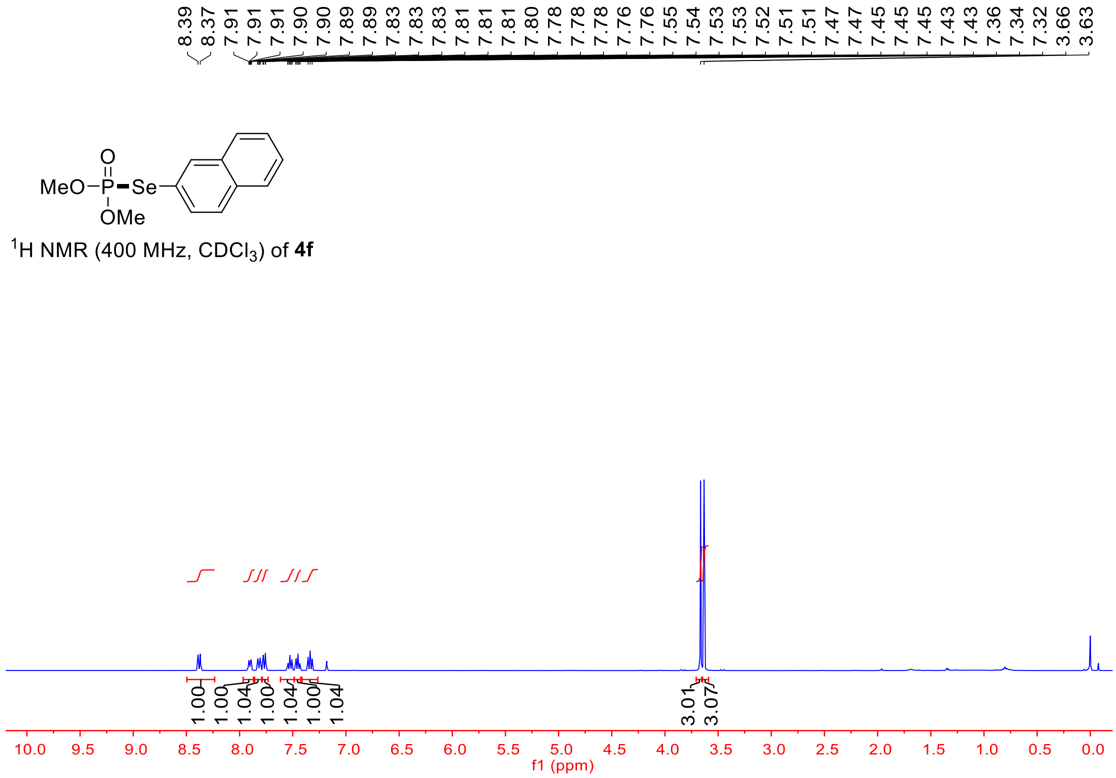

Figure S54: ^{1}H NMR spectrum for compound **4f**

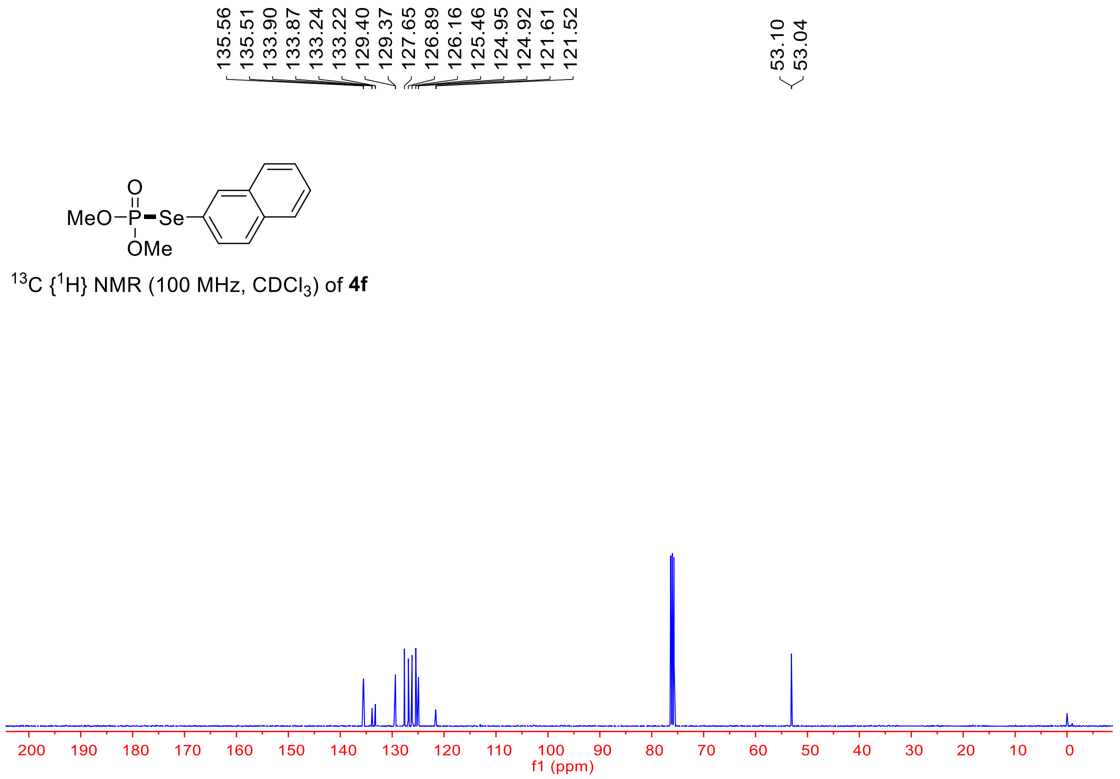

Figure S55: ^{13}C NMR spectrum for compound **4f**

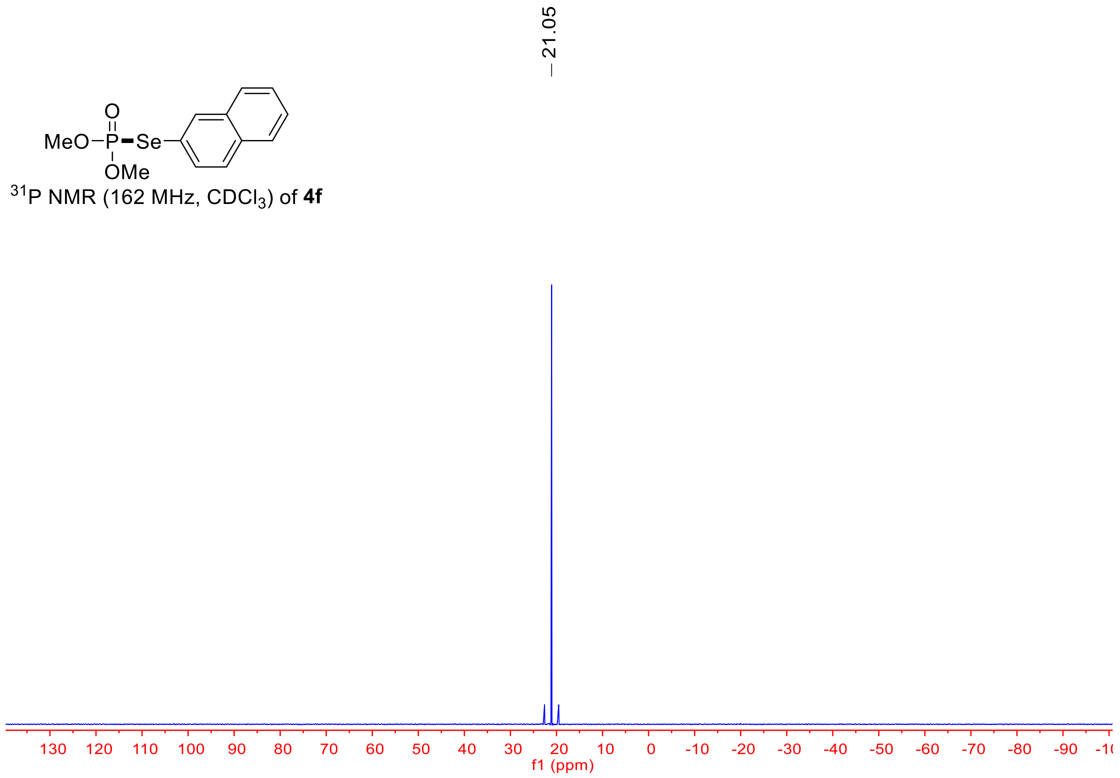

Figure S56: ^{31}P NMR spectrum for compound **4f**

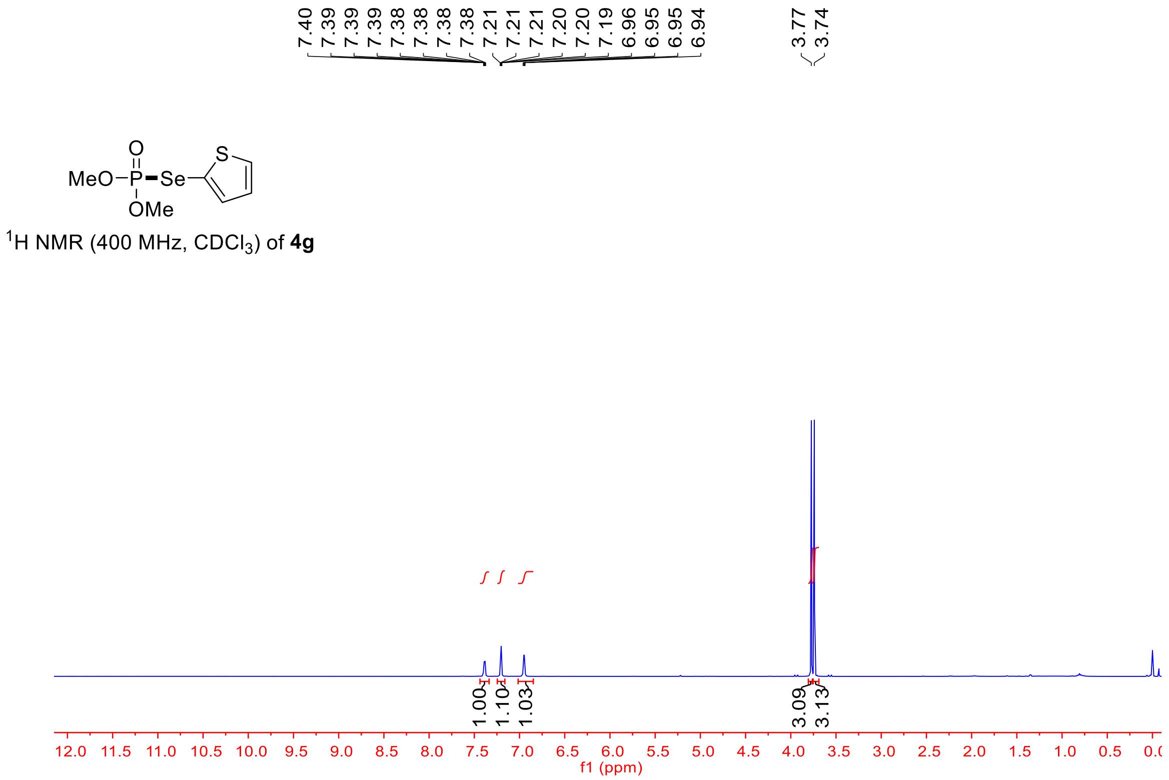

Figure S57: ^{1}H NMR spectrum for compound **4g**

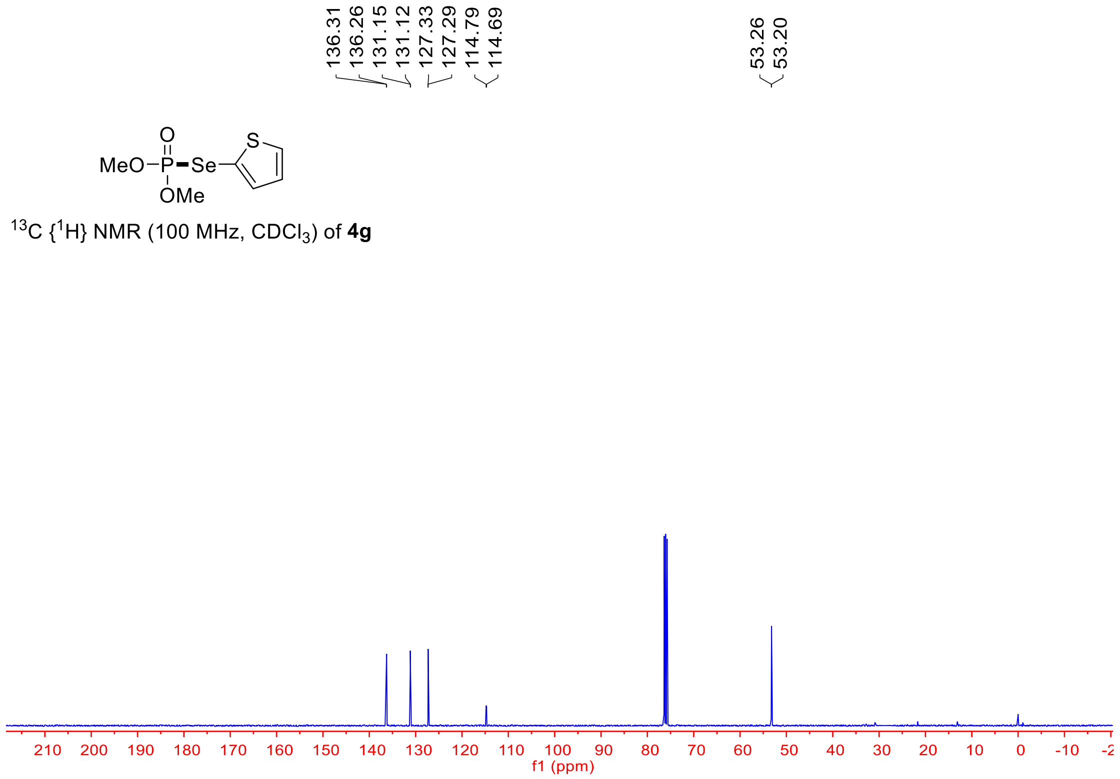

Figure S58: ^{13}C NMR spectrum for compound **4g**

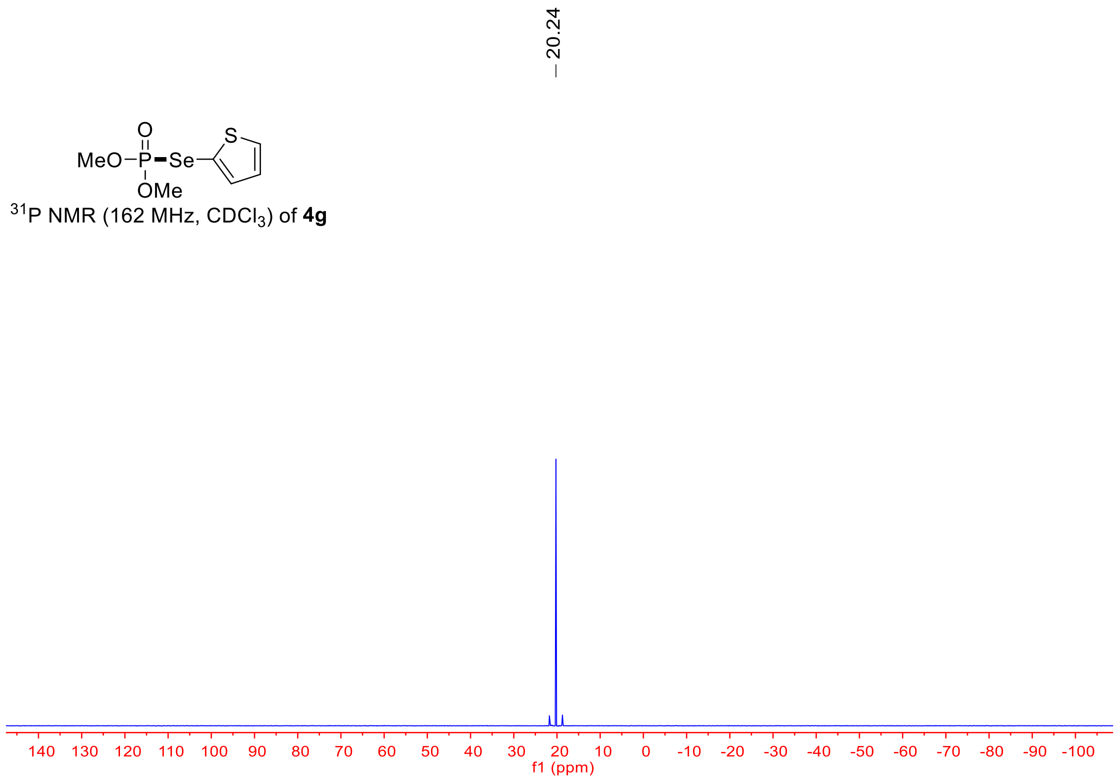

Figure S59: ^{31}P NMR spectrum for compound **4g**

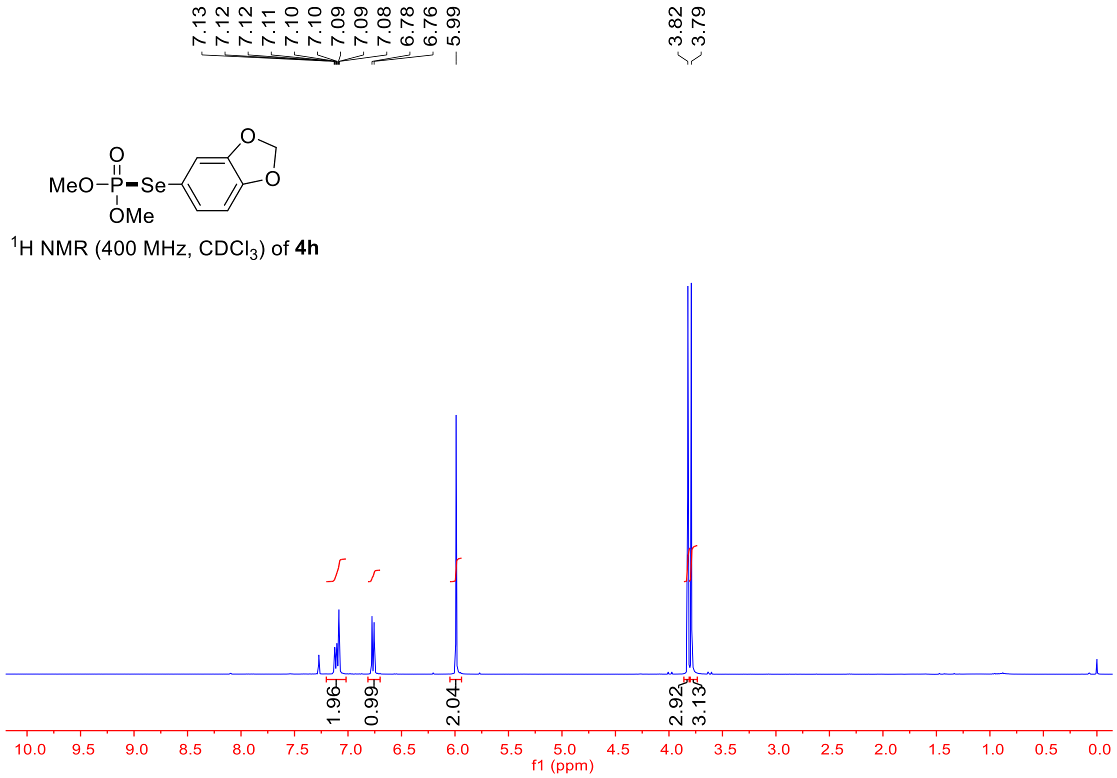

Figure S60: ^{1}H NMR spectrum for compound **4h**

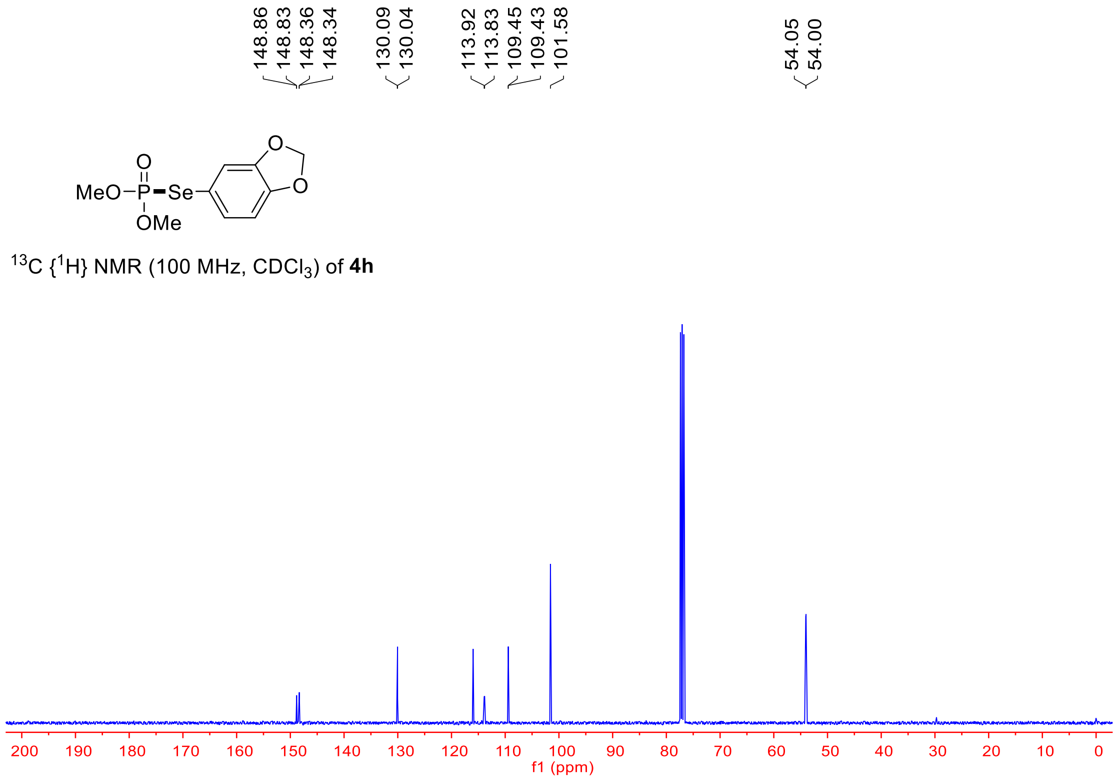

Figure S61: ^{13}C NMR spectrum for compound **4h**

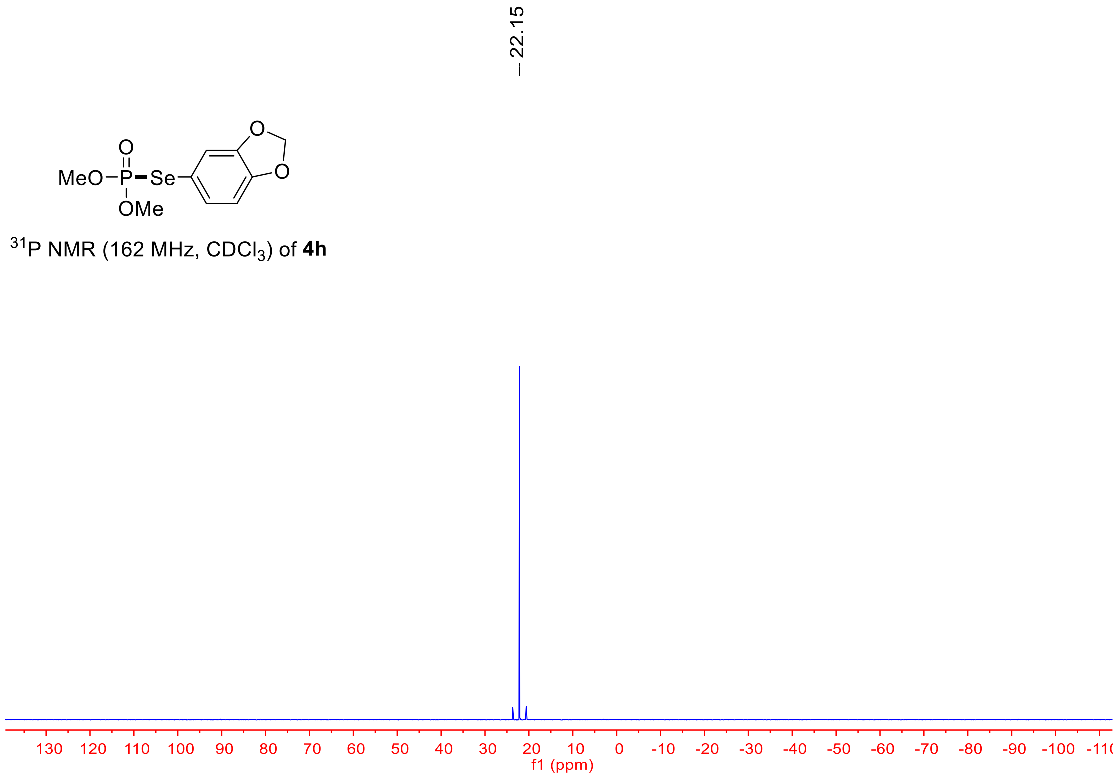

Figure S62: ^{31}P NMR spectrum for compound **4h**

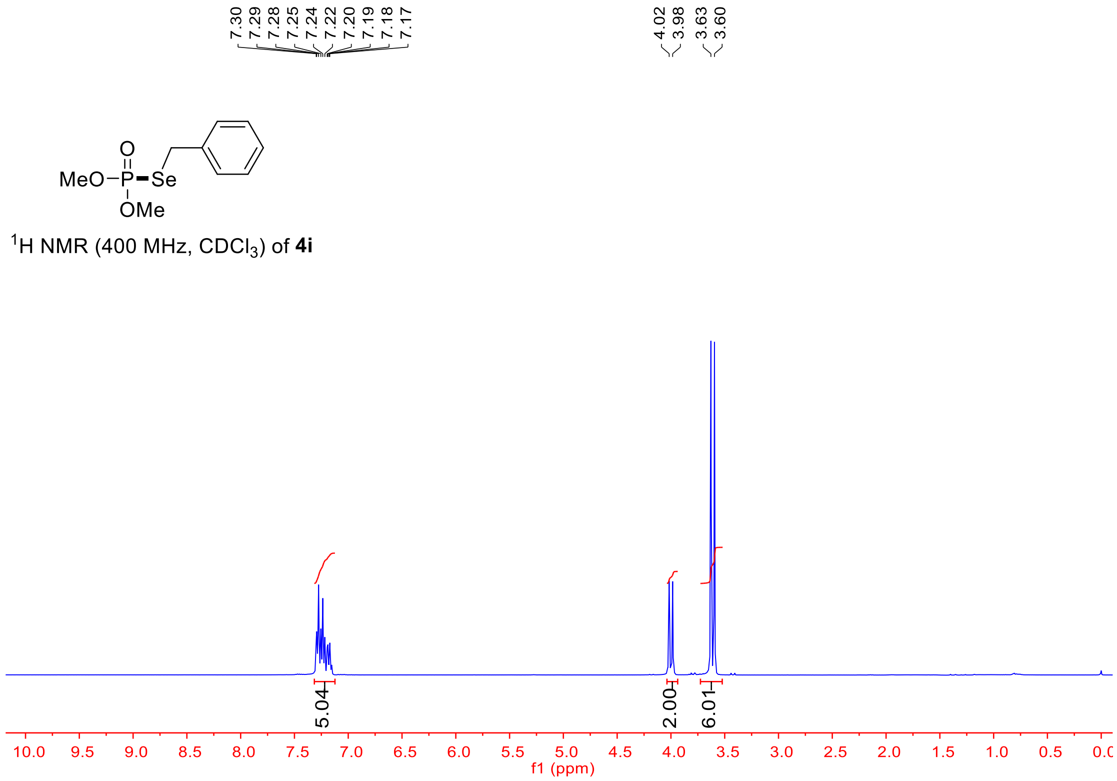

Figure S63: ^{1}H NMR spectrum for compound **4i**

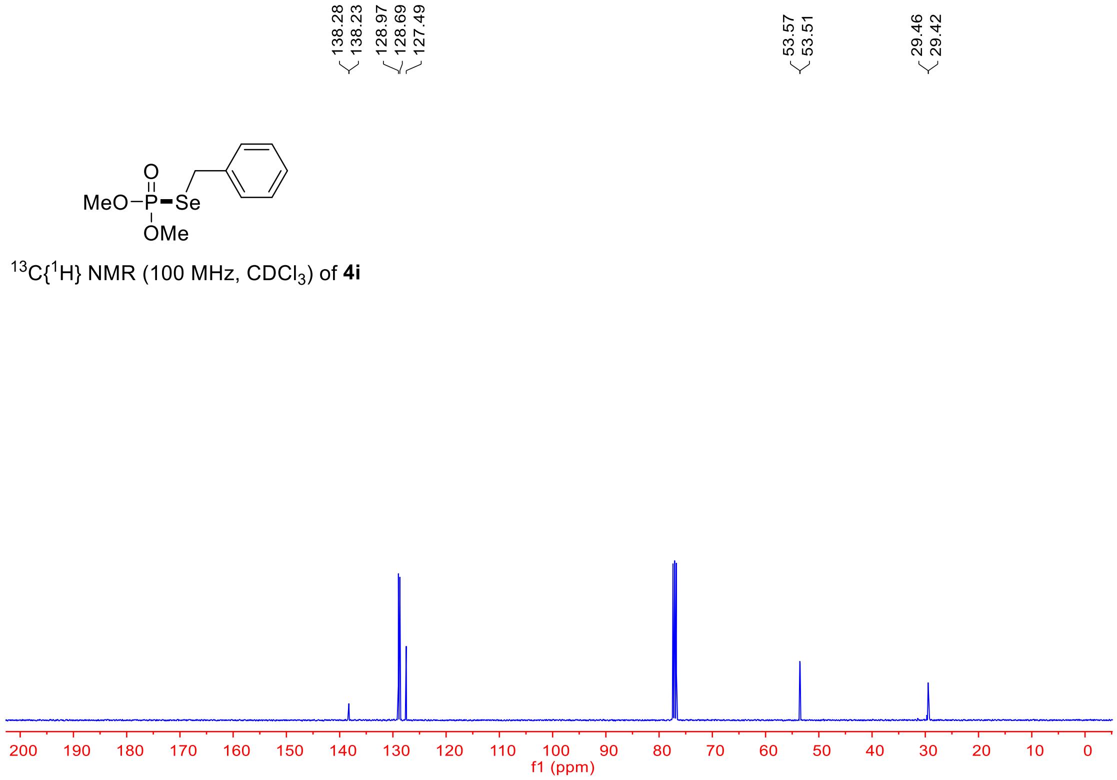

Figure S64: ^{13}C NMR spectrum for compound **4i**

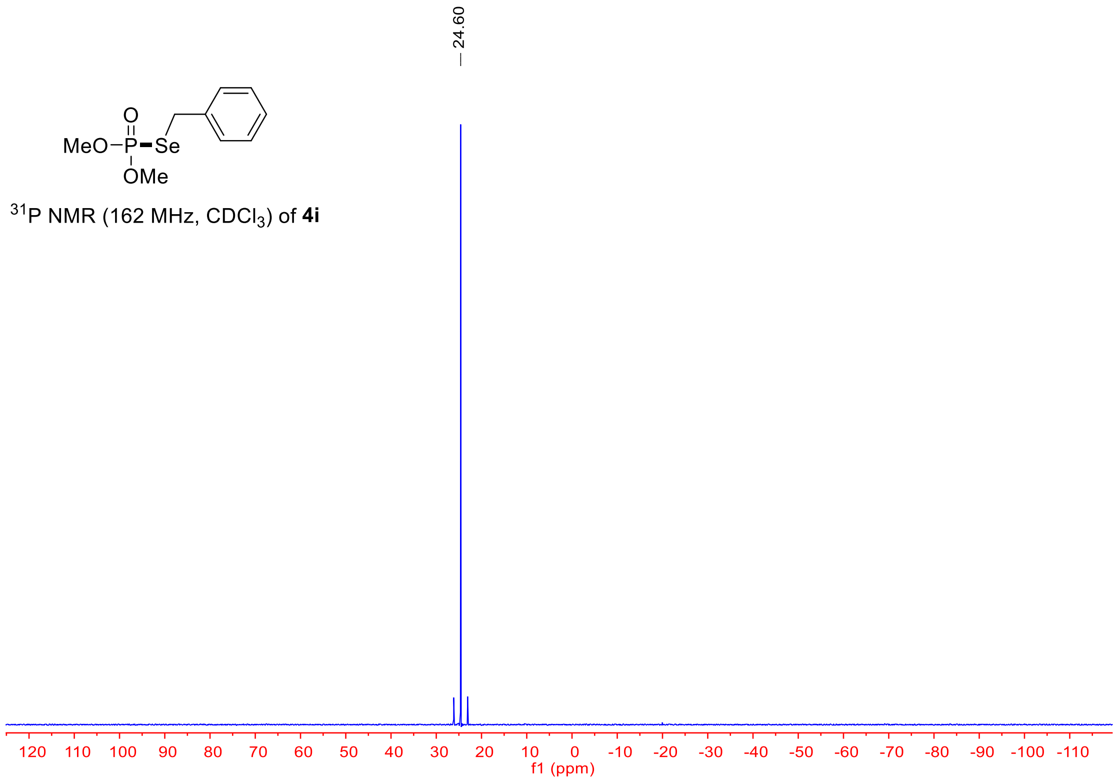

Figure S65: ^{31}P NMR spectrum for compound **4i**

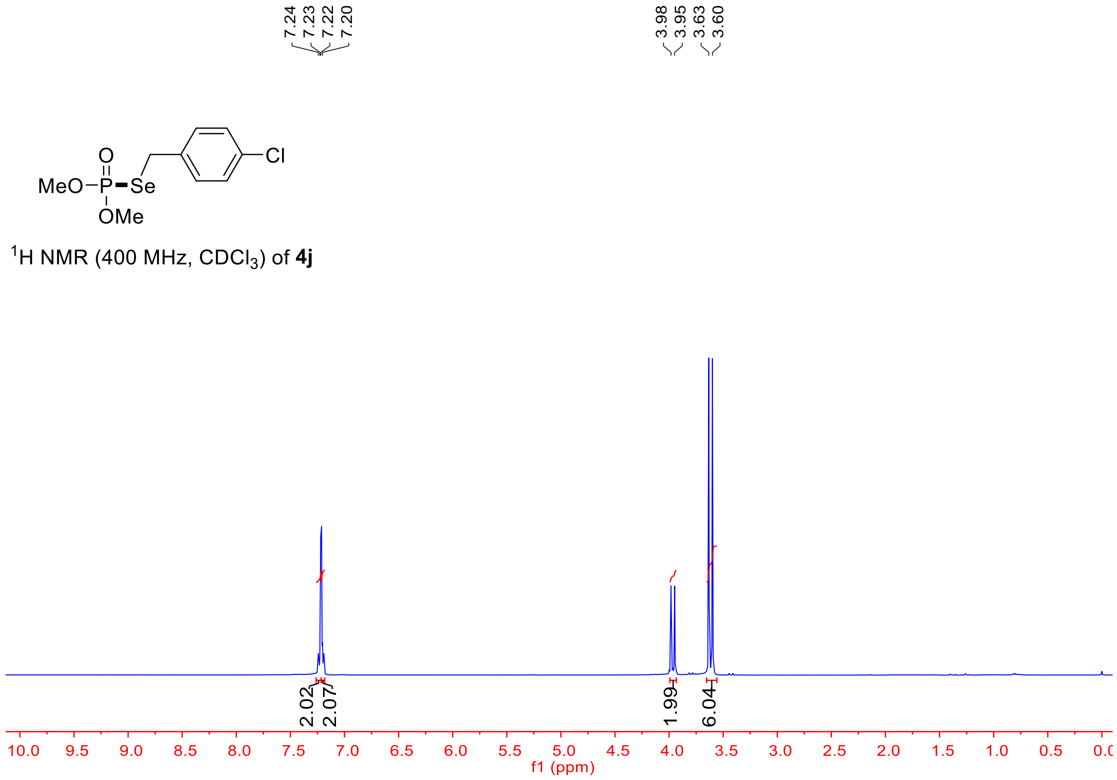

Figure S66: ^{1}H NMR spectrum for compound **4j**

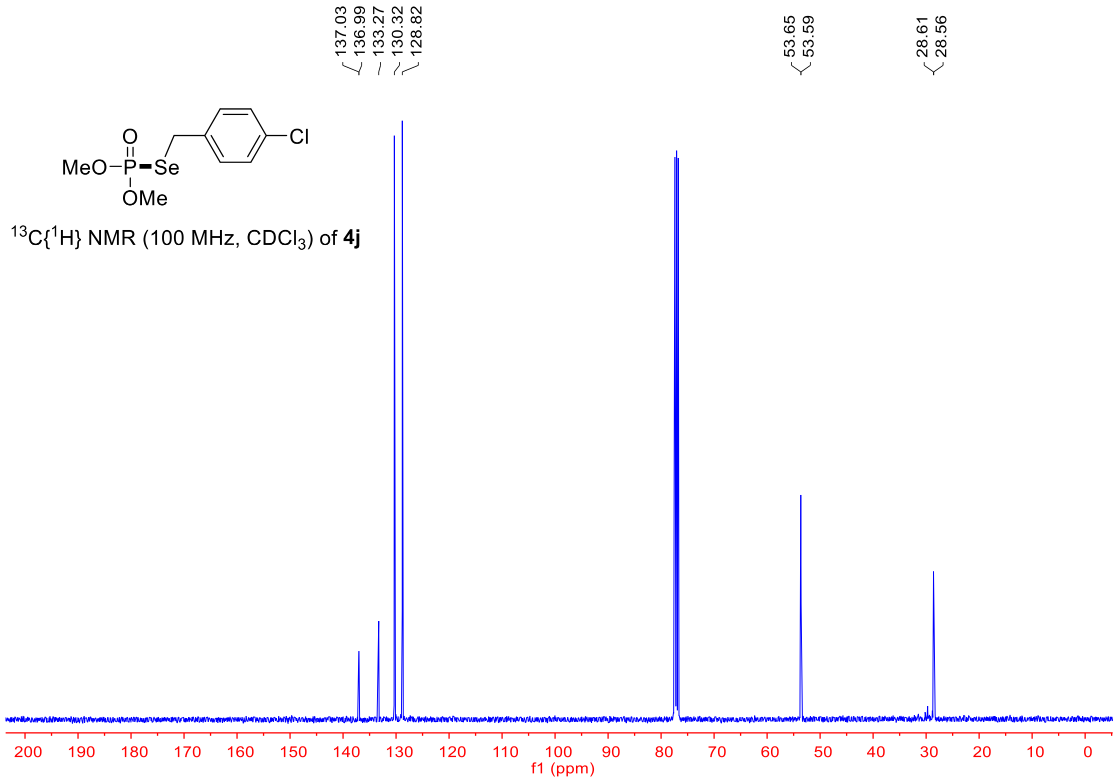

Figure S67: ^{13}C NMR spectrum for compound **4j**

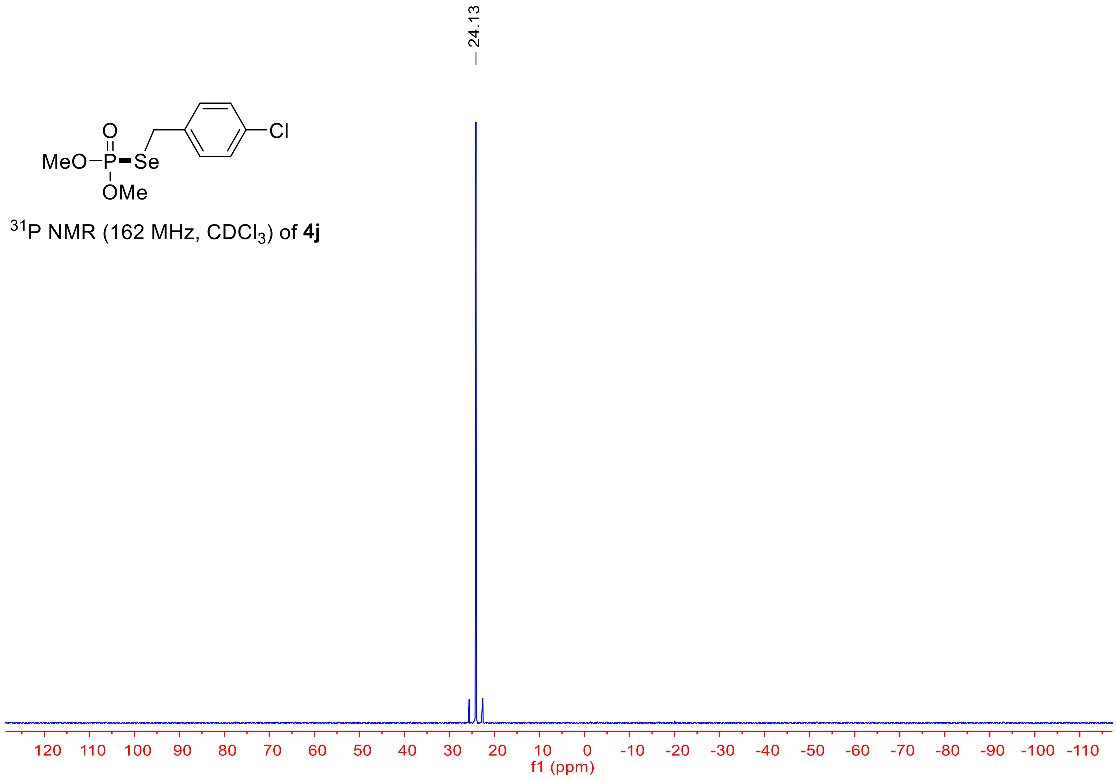

Figure S68: ^{31}P NMR spectrum for compound **4j**

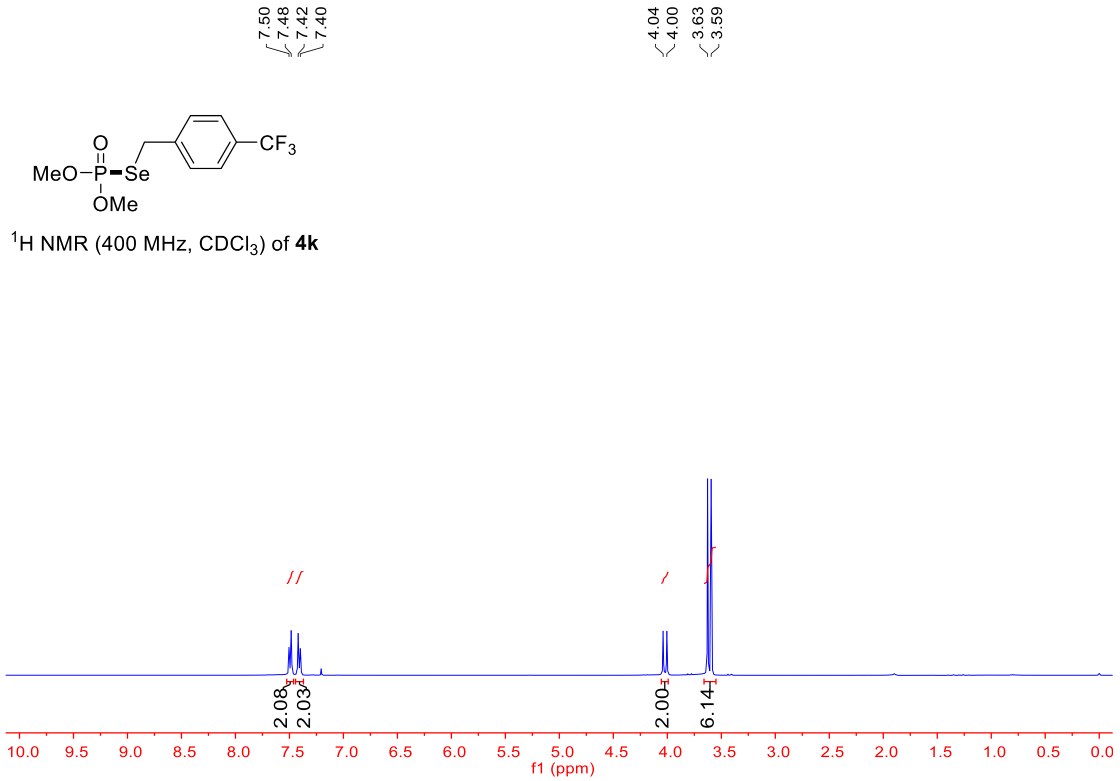

Figure S69: ^{1}H NMR spectrum for compound **4k**

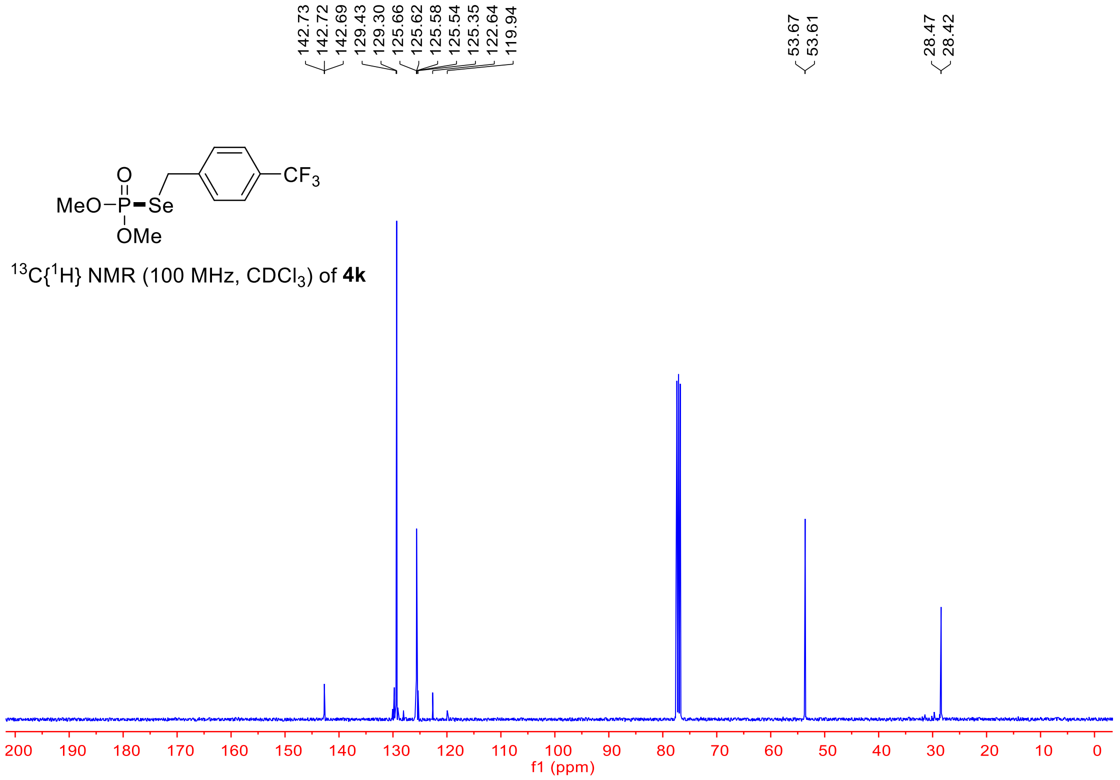

Figure S70: ^{13}C NMR spectrum for compound **4k**

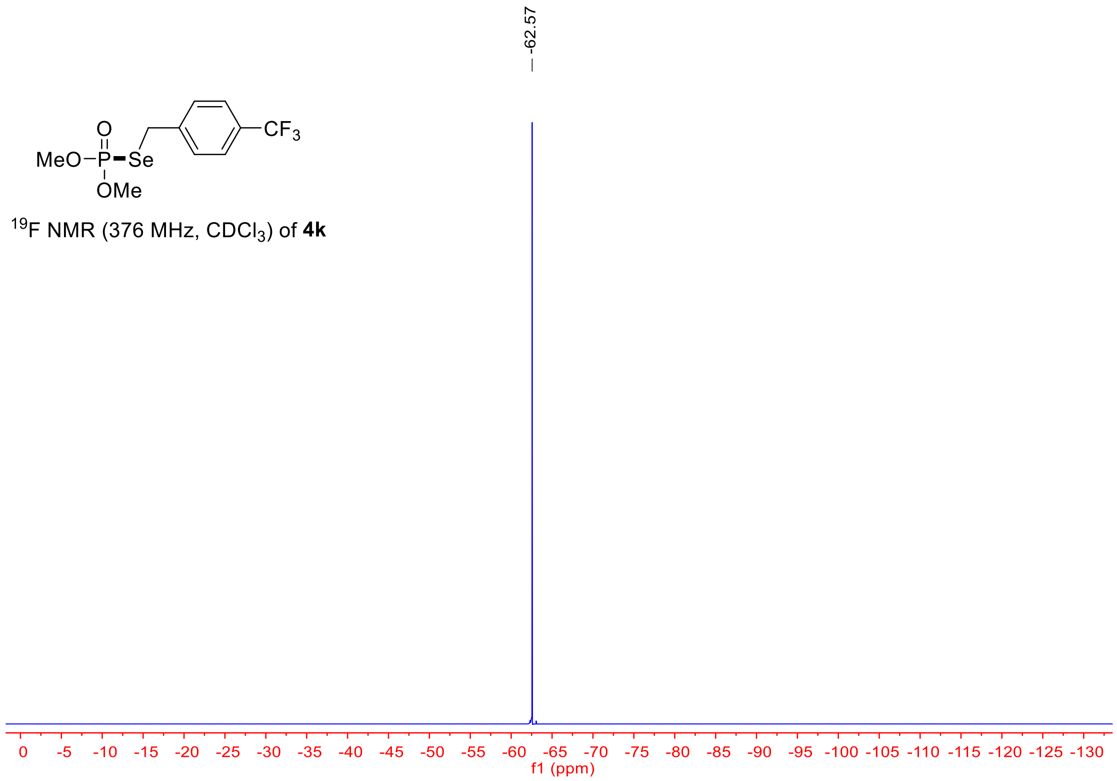

Figure S71: ^{19}F NMR spectrum for compound **4k**

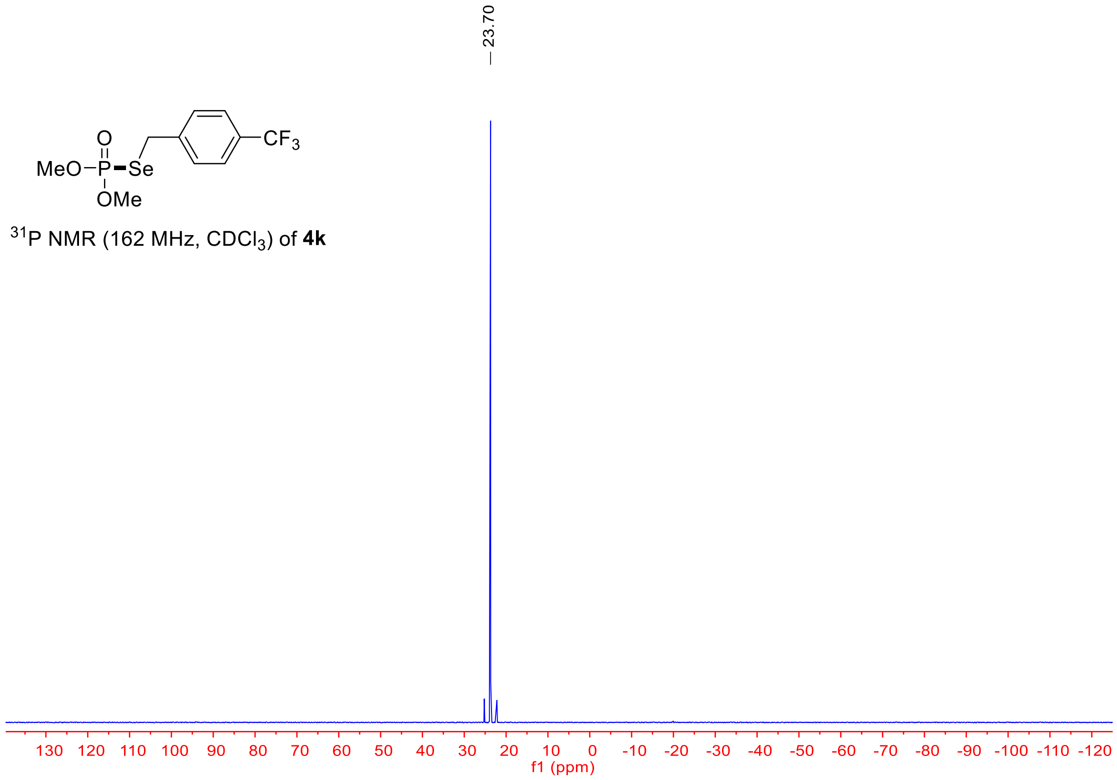

Figure S72: ^{31}P NMR spectrum for compound **4k**

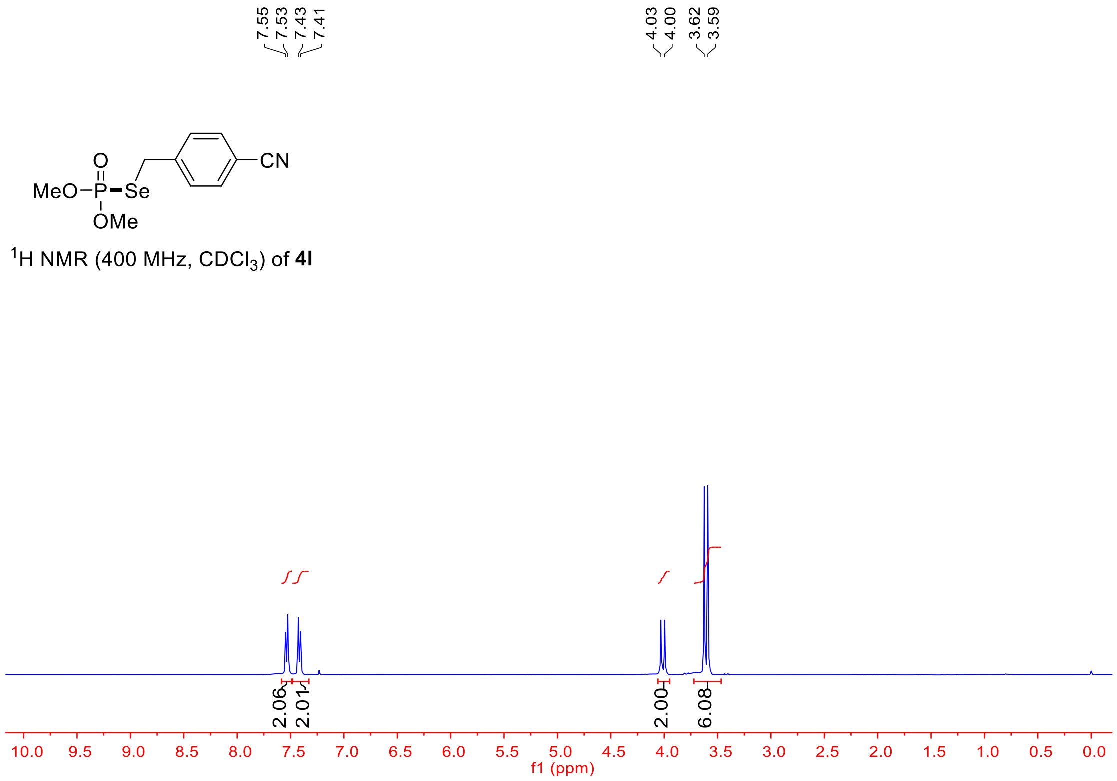

Figure S73: ^{1}H NMR spectrum for compound **4l**

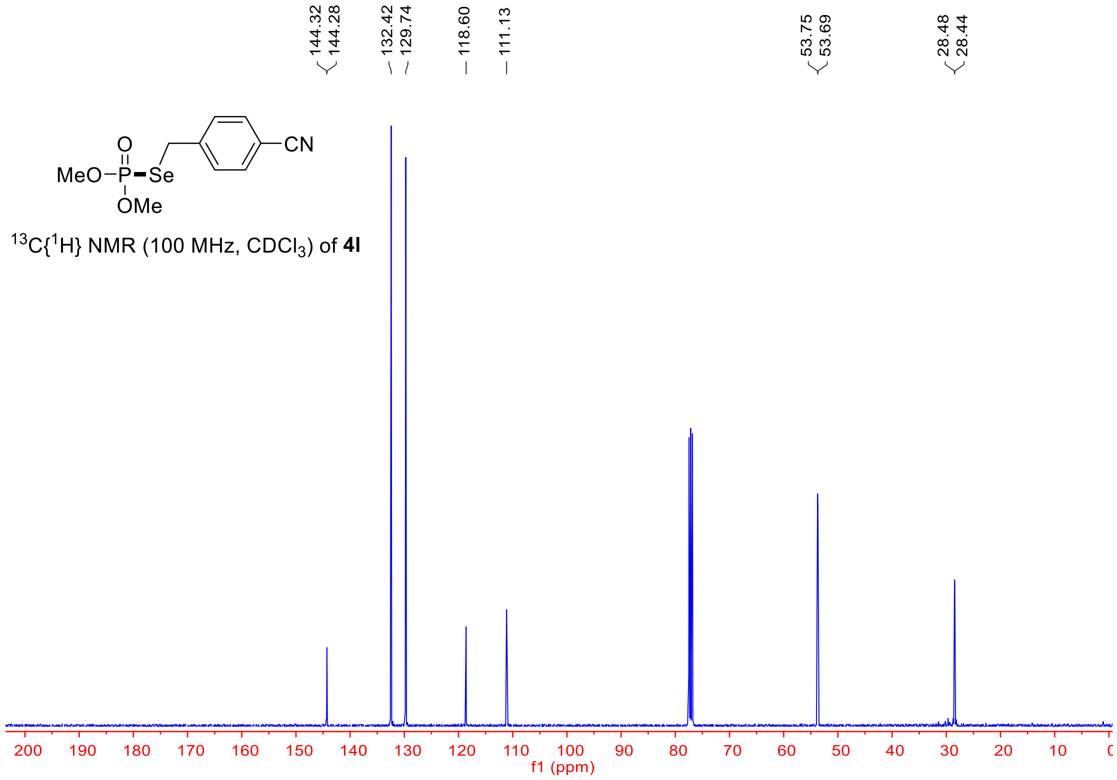

Figure S74: ^{13}C NMR spectrum for compound **4l**

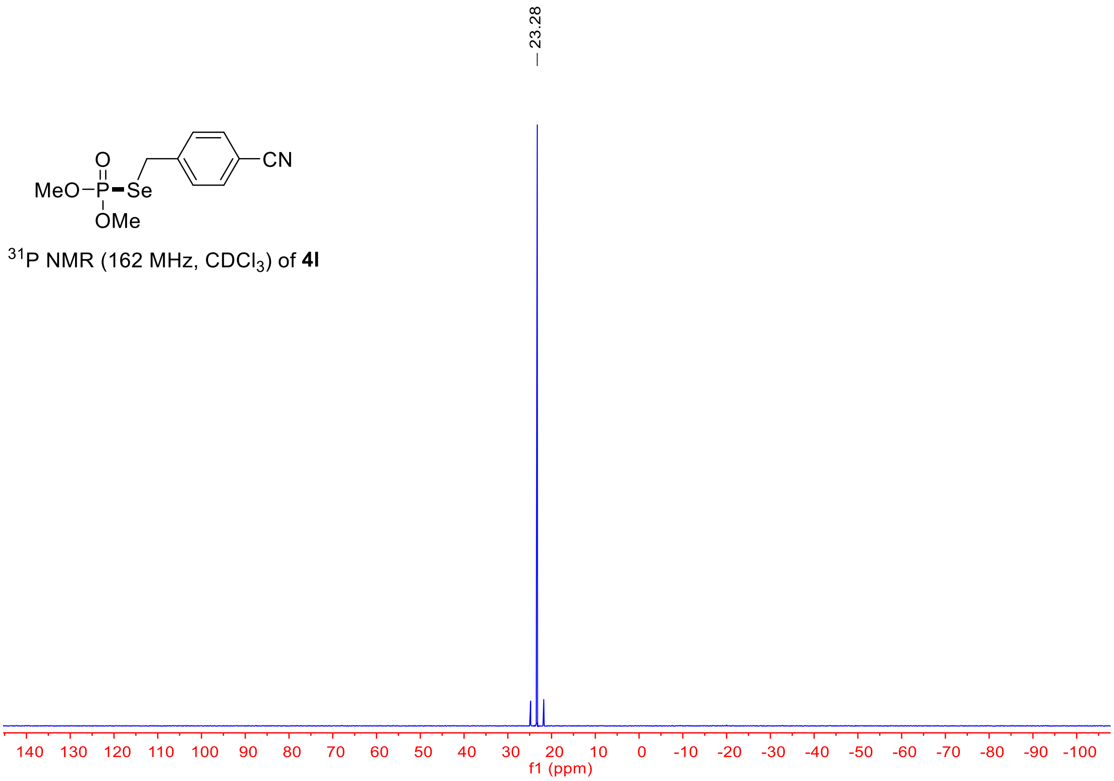

Figure S75: ^{31}P NMR spectrum for compound **4l**

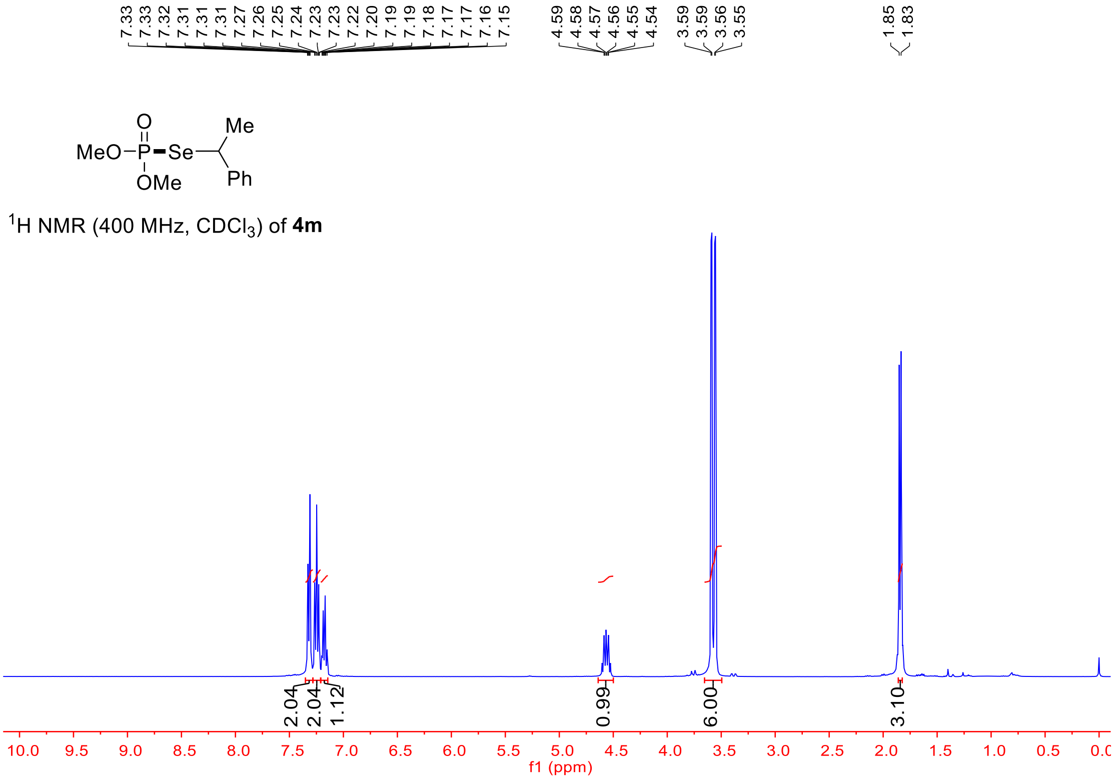

Figure S76: ^{1}H NMR spectrum for compound **4m**

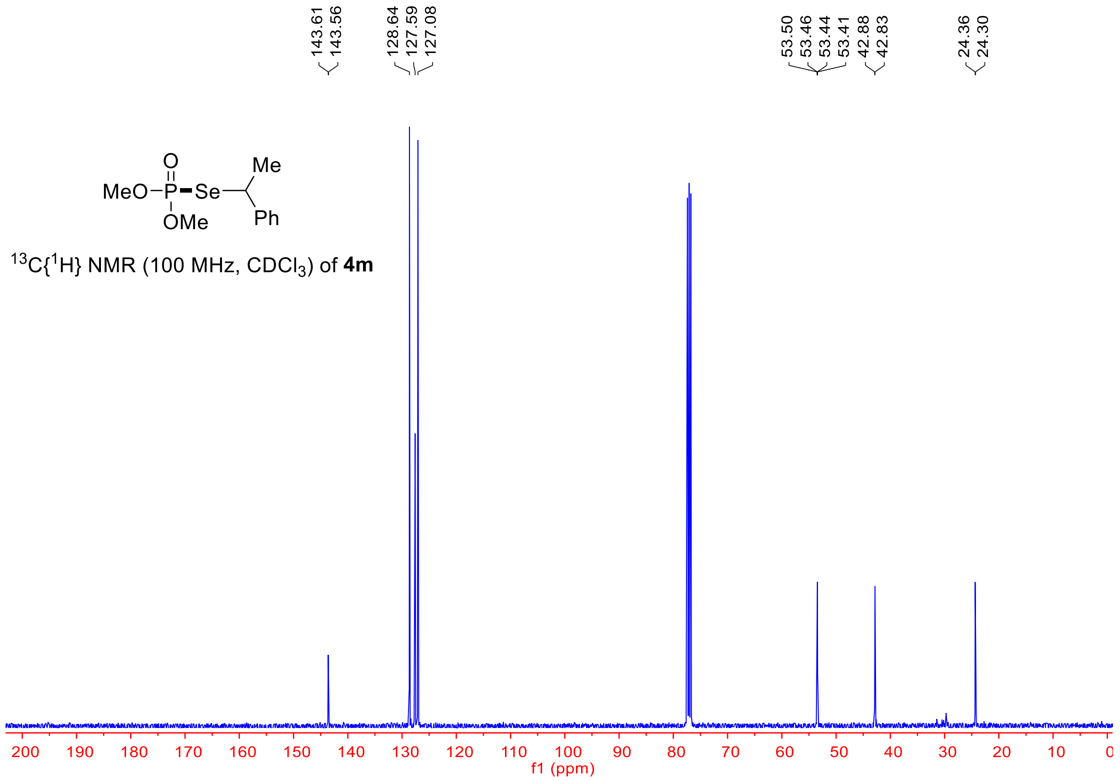

Figure S77: ^{13}C NMR spectrum for compound **4m**

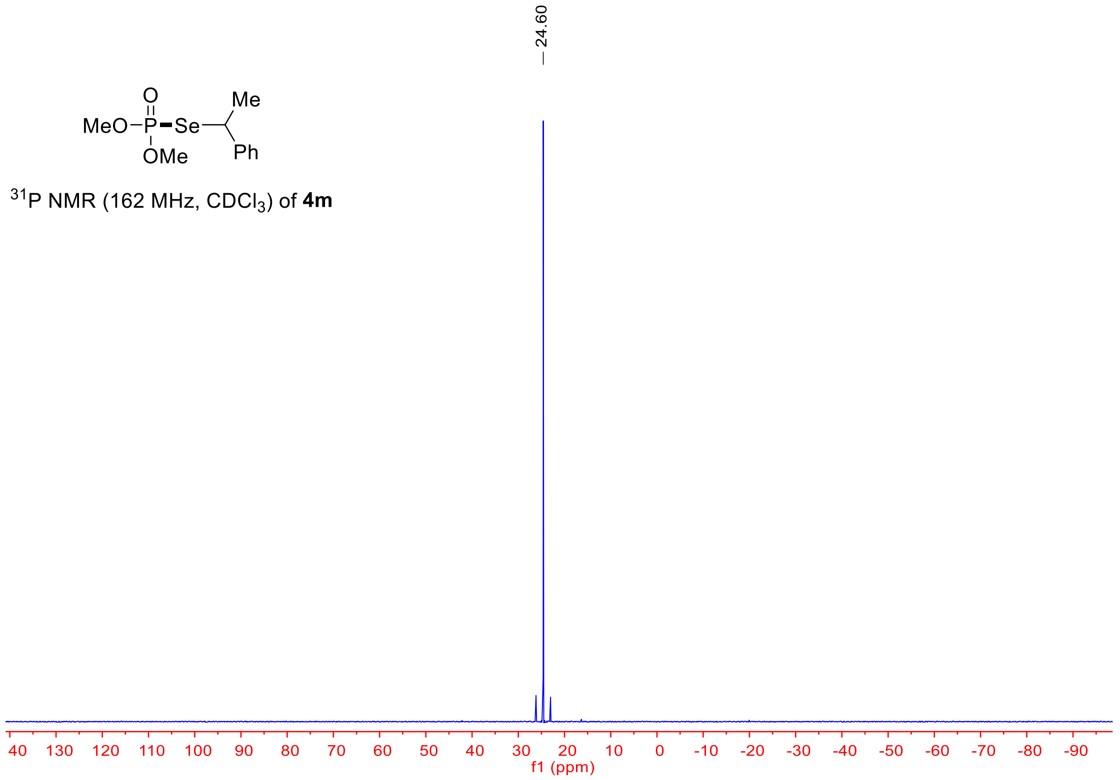

Figure S78: ^{31}P NMR spectrum for compound **4m**

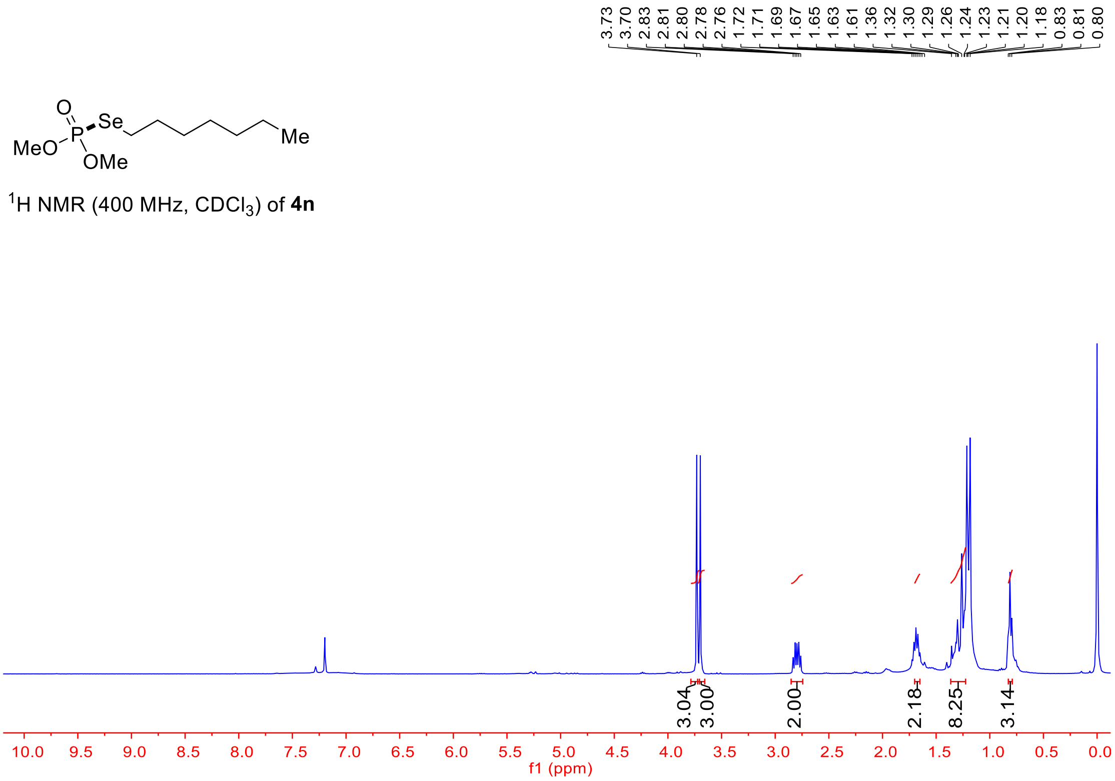

Figure S79: ^{1}H NMR spectrum for compound **4n**

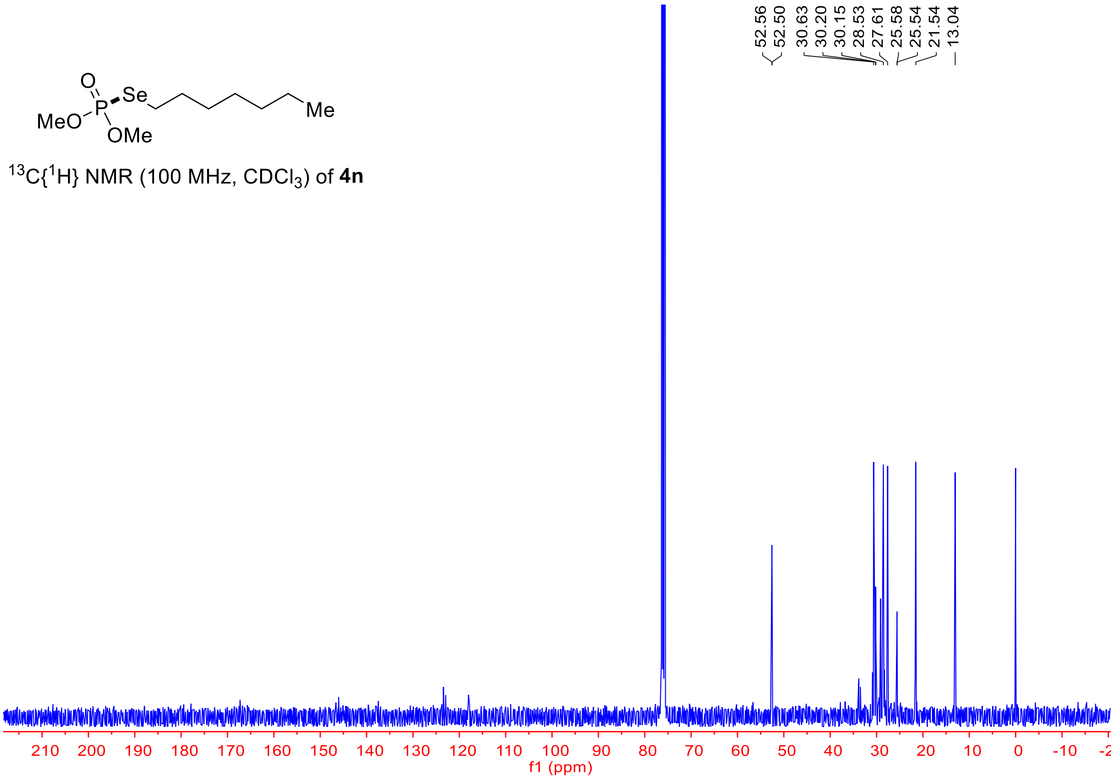

Figure S80: ^{13}C NMR spectrum for compound **4n**

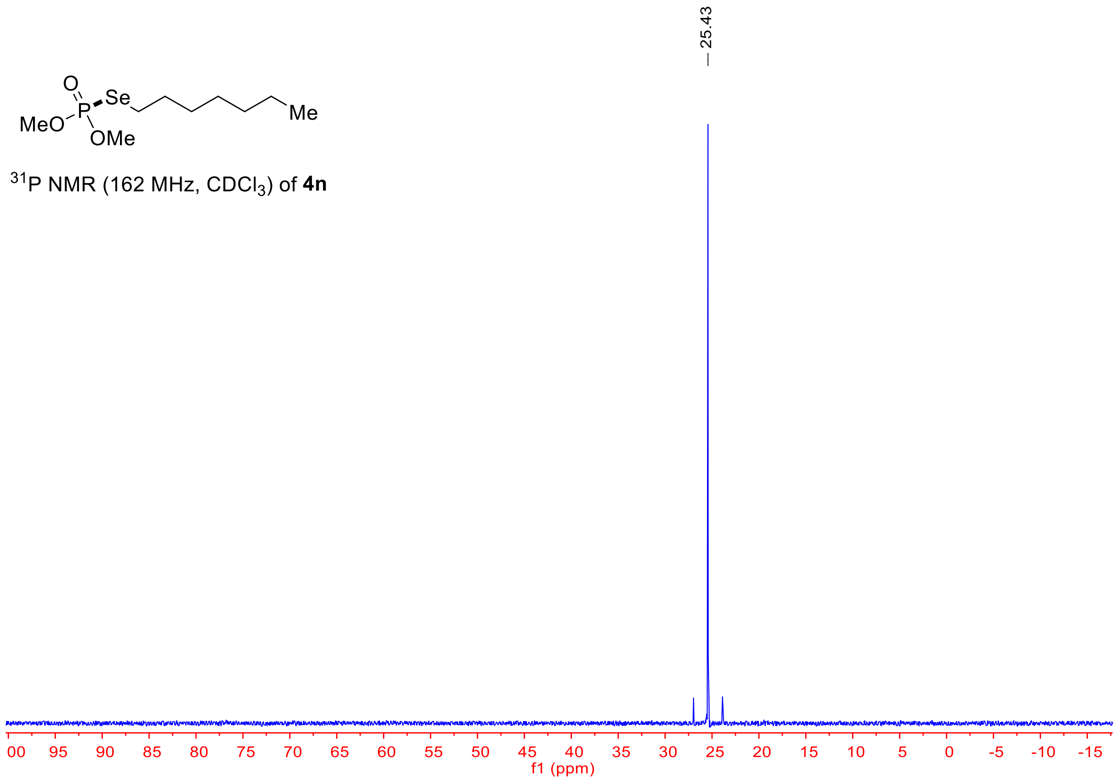

Figure S81: ^{31}P NMR spectrum for compound **4n**

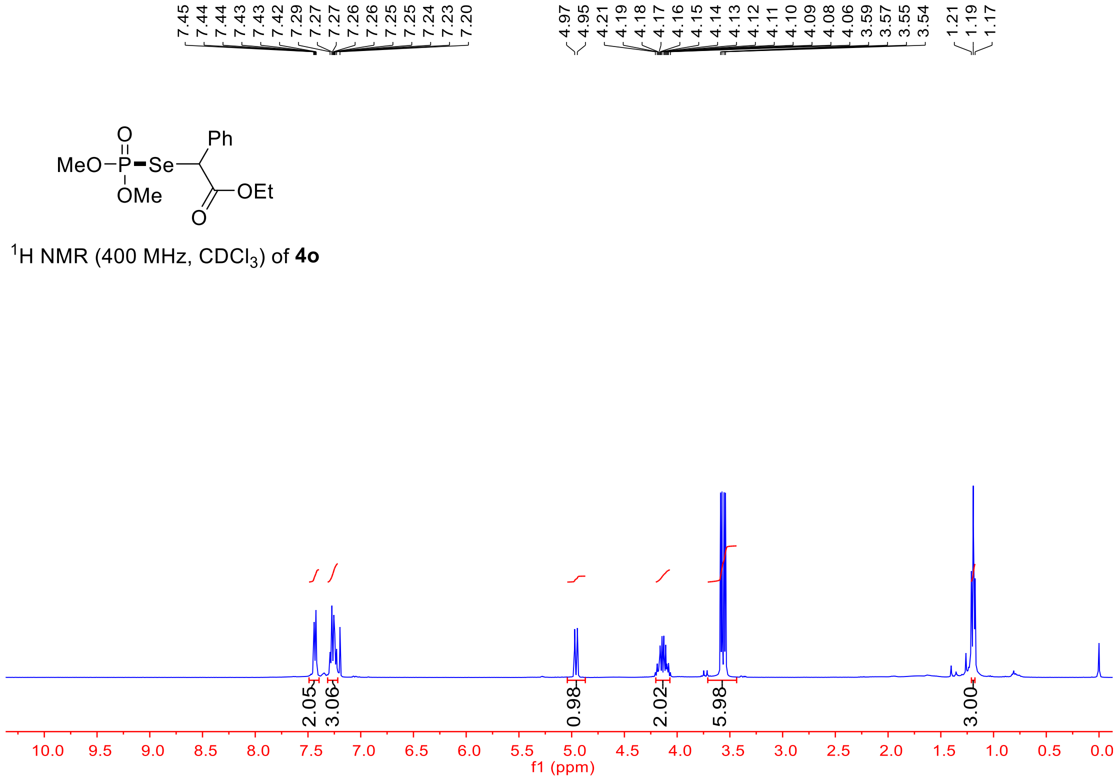

Figure S82: ^{1}H NMR spectrum for compound **4o**

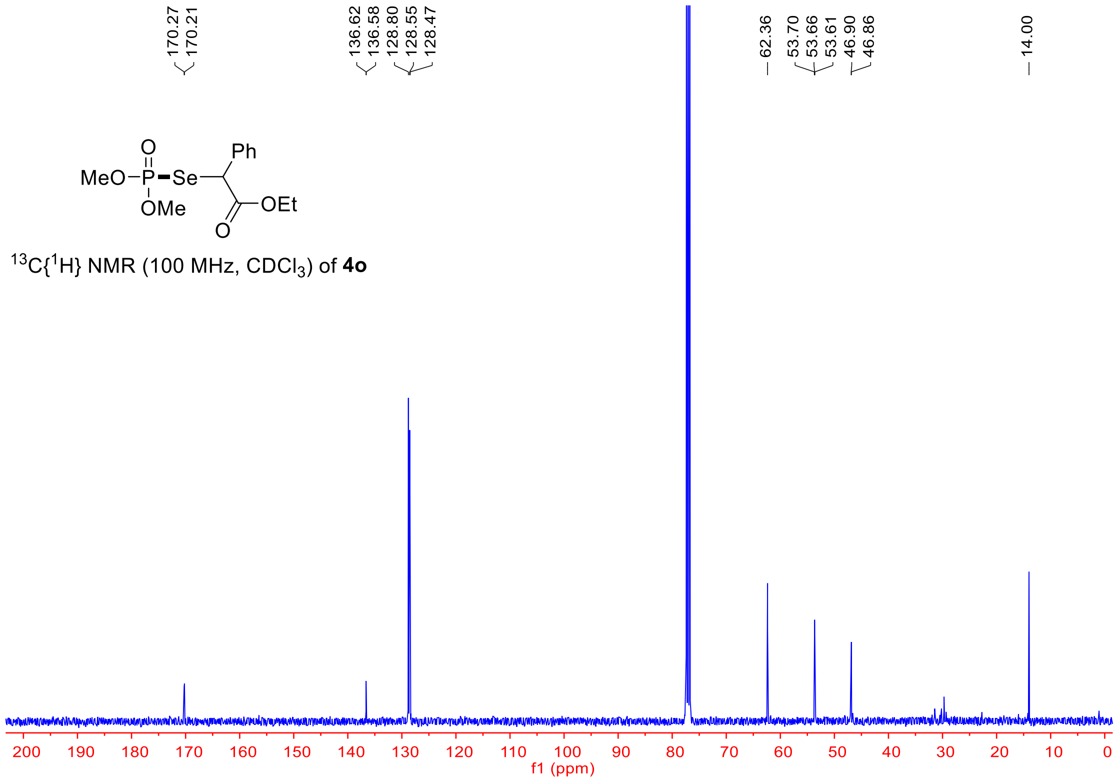

Figure S83: ^{13}C NMR spectrum for compound **4o**

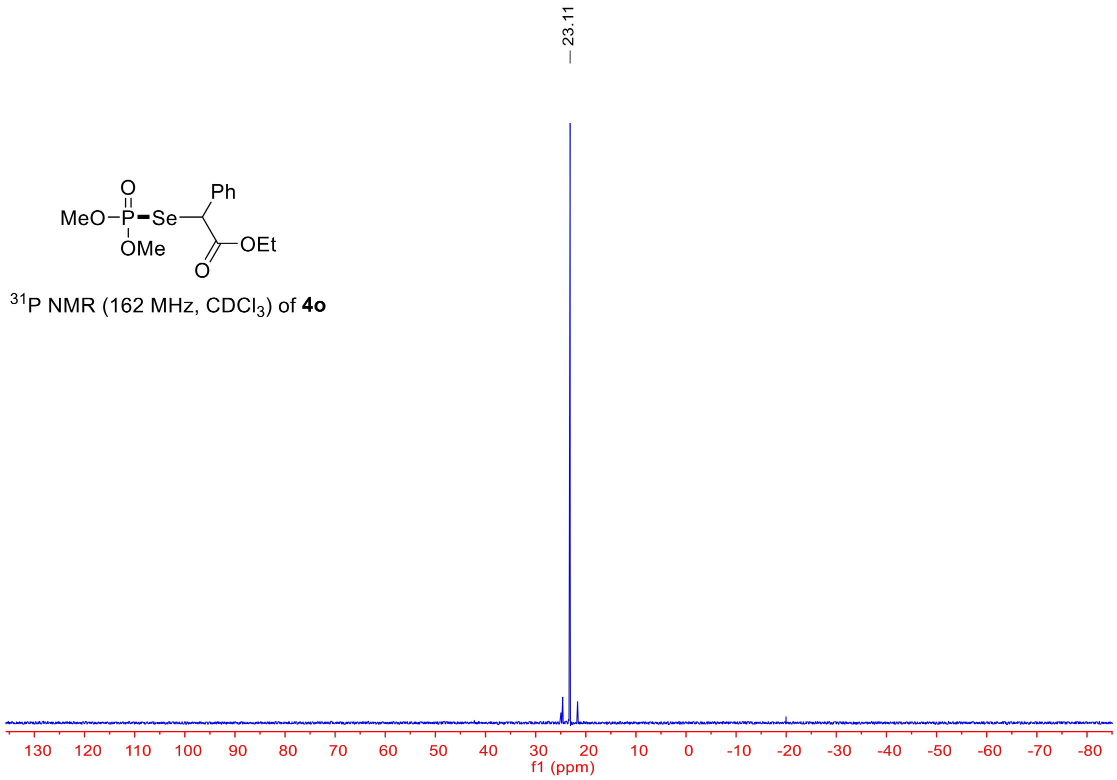

Figure S84: ^{31}P NMR spectrum for compound **4o**

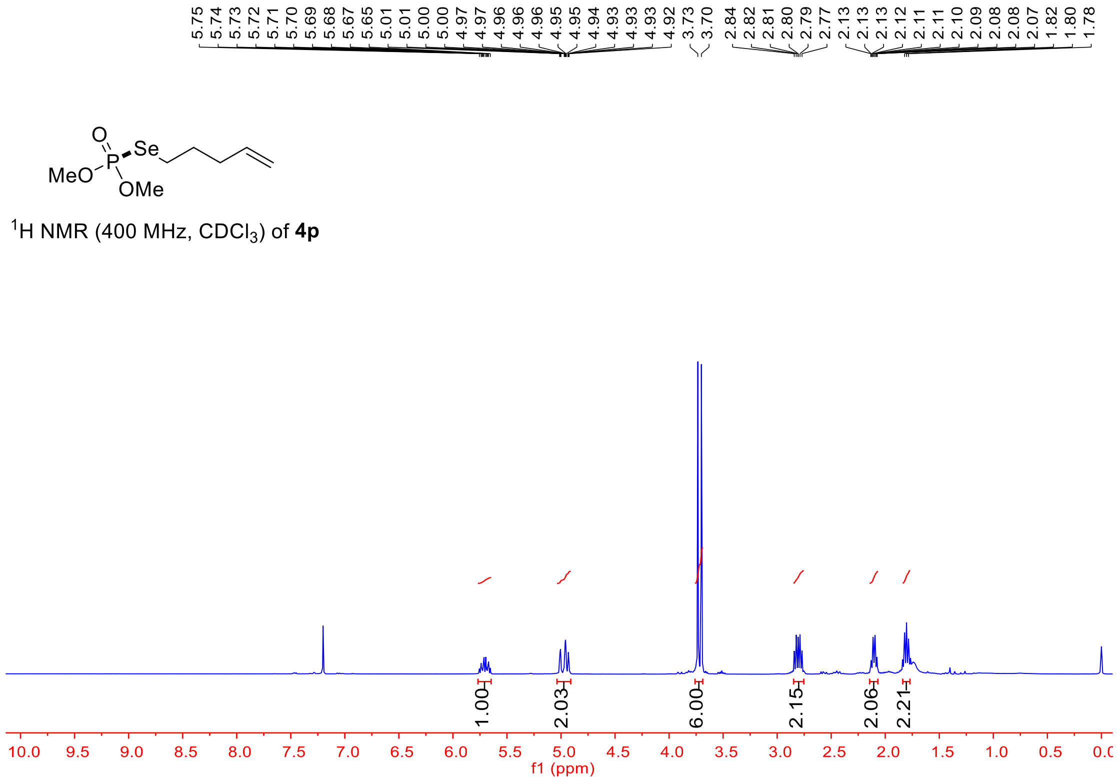

Figure S85: ^{1}H NMR spectrum for compound **4p**

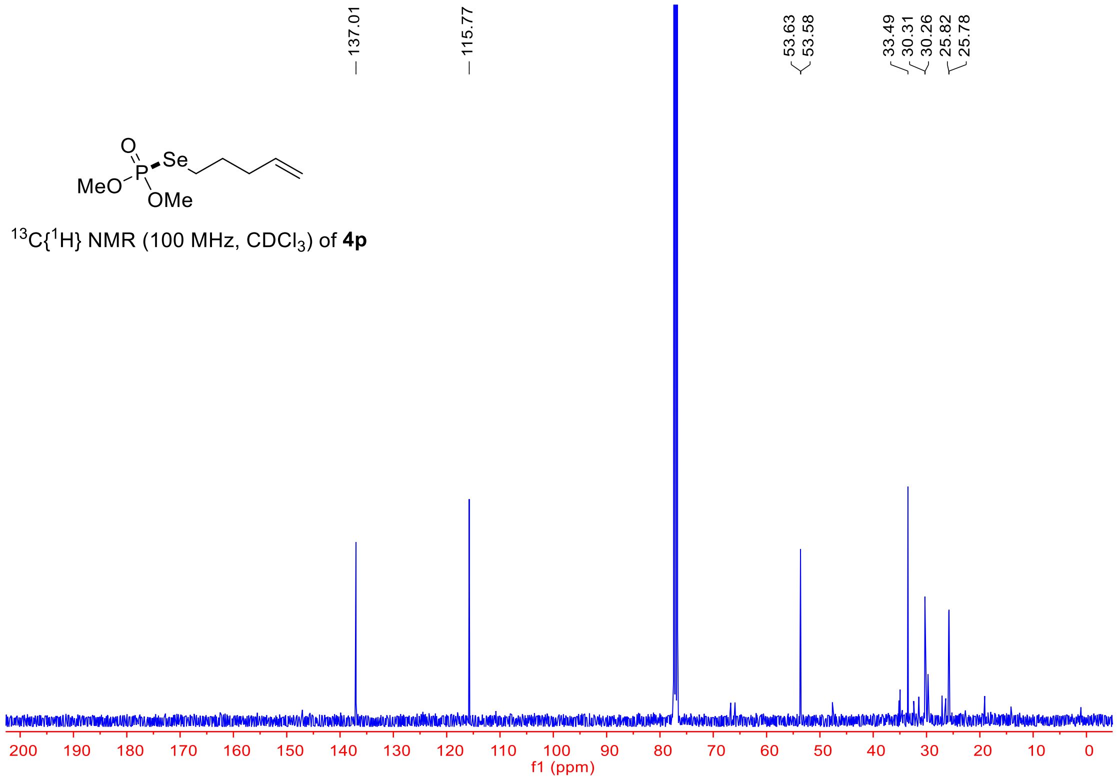

Figure S86: ^{13}C NMR spectrum for compound **4p**

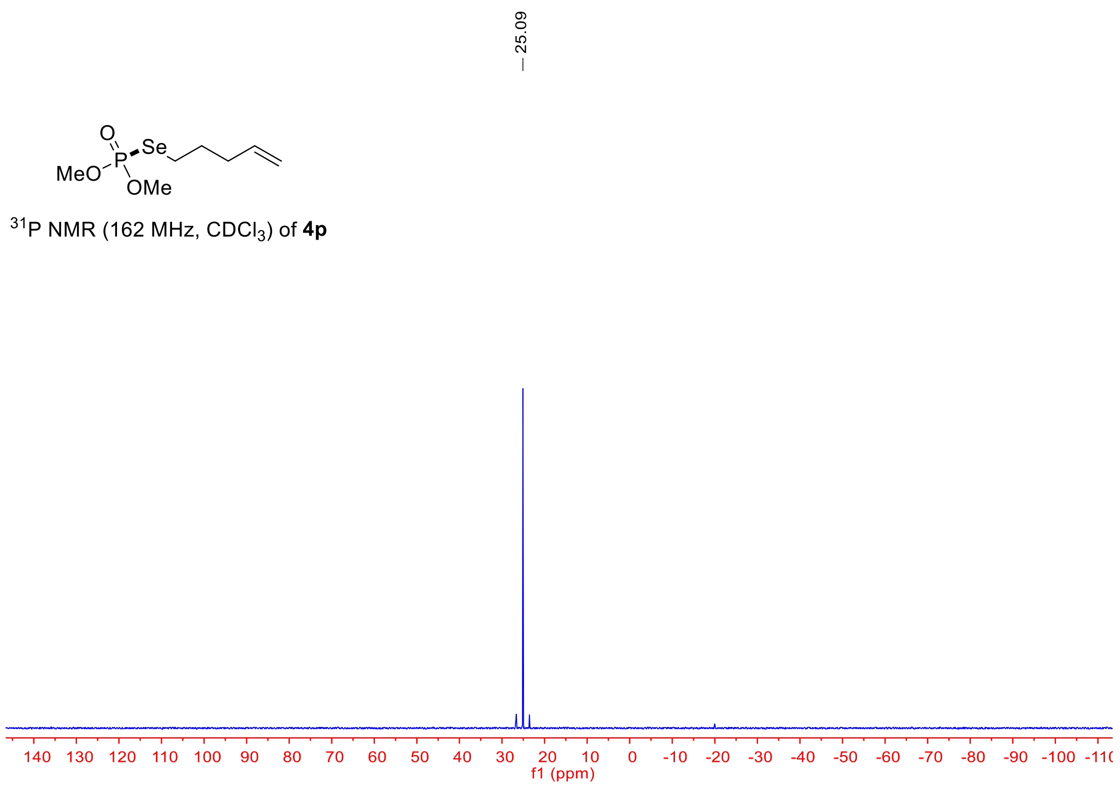

Figure S87: ^{31}P NMR spectrum for compound **4p**

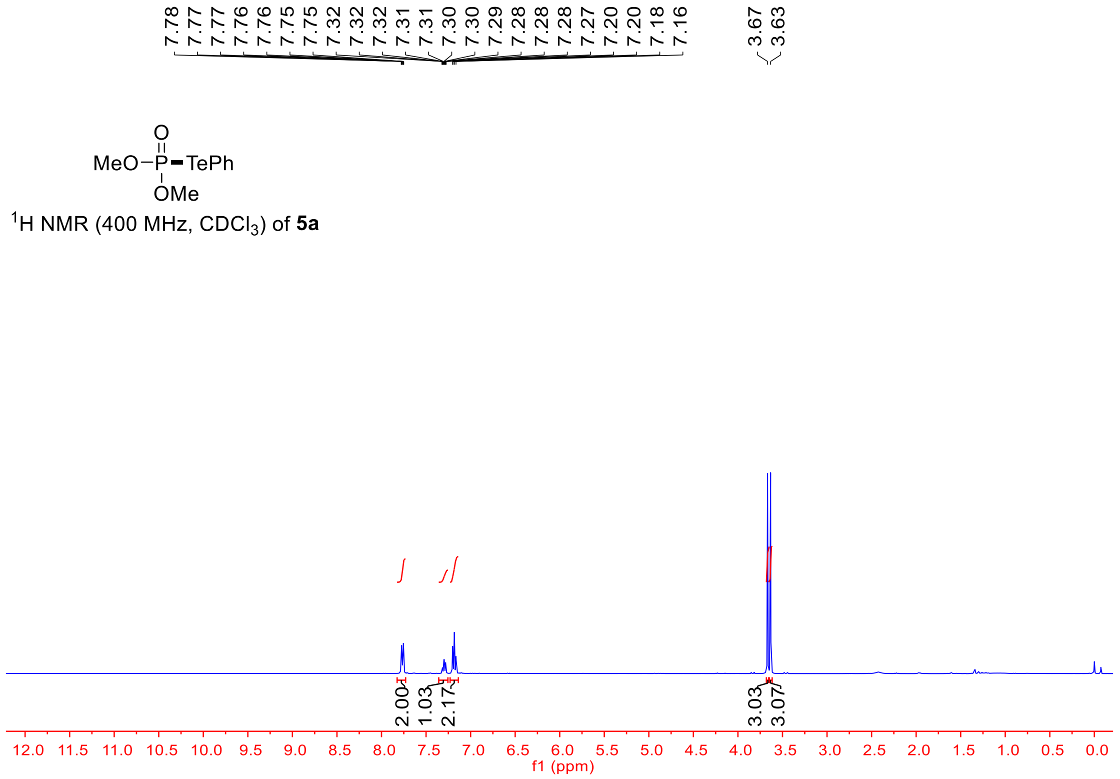

Figure S88: ^{1}H NMR spectrum for compound **5a**

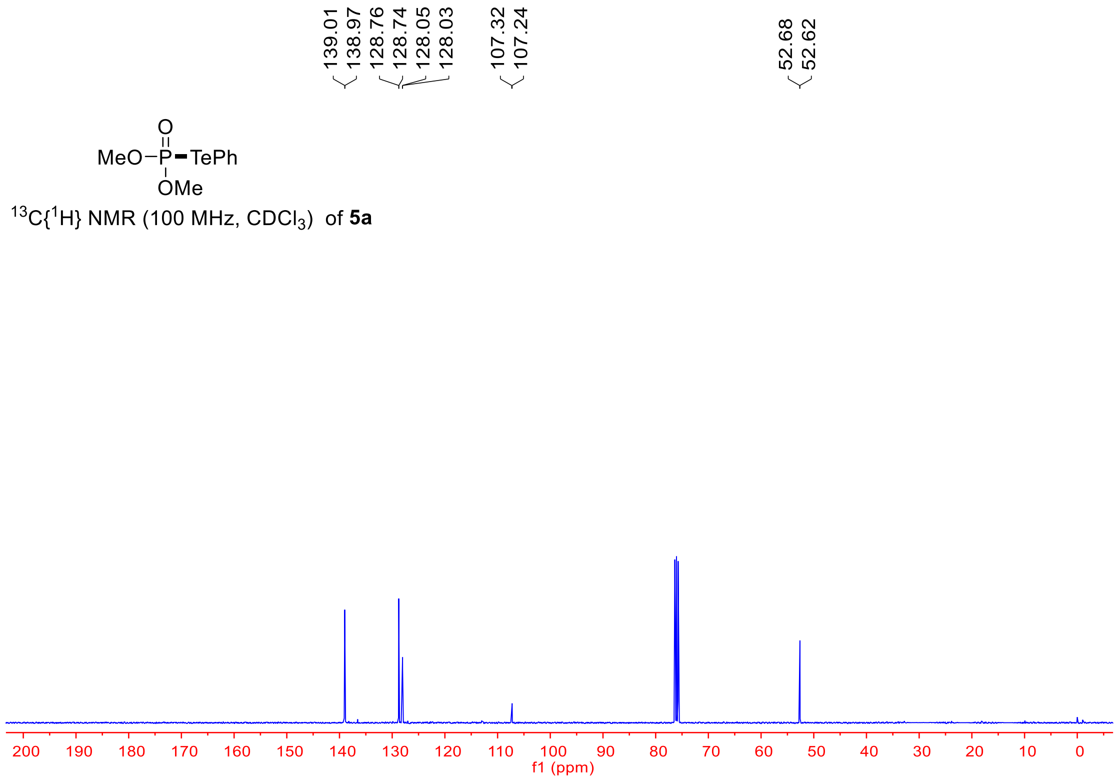

Figure S89: ^{13}C NMR spectrum for compound **5a**

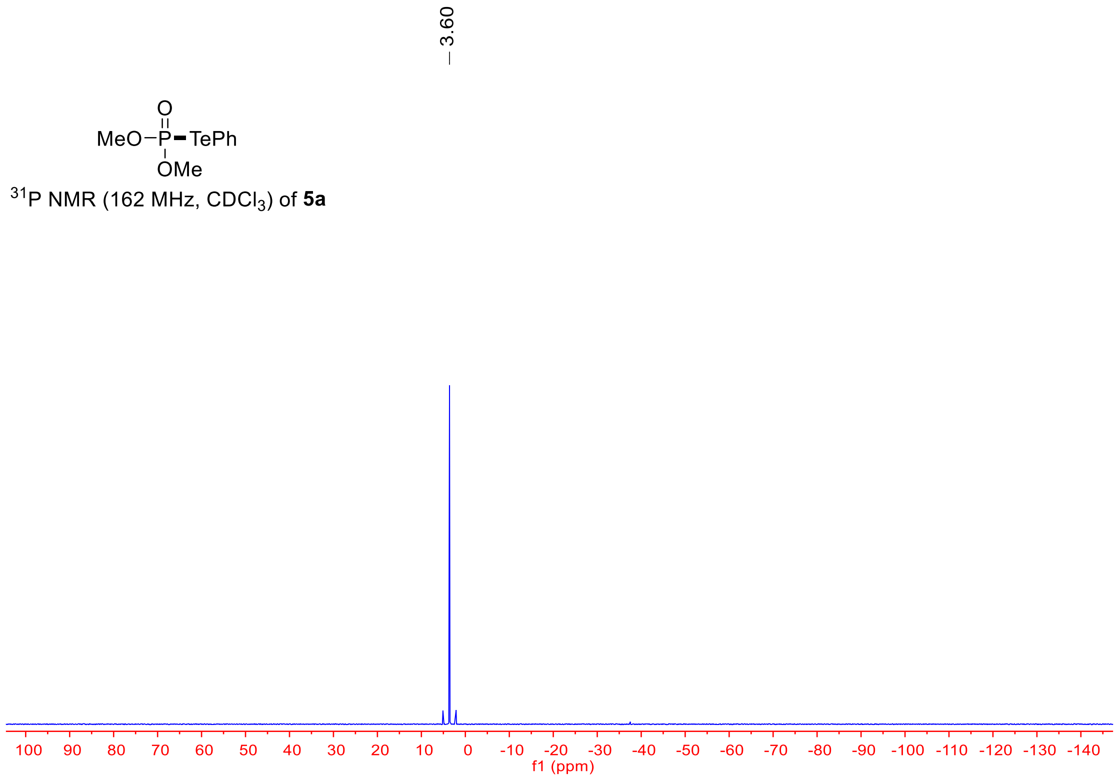

Figure S90: ^{31}P NMR spectrum for compound **5a**

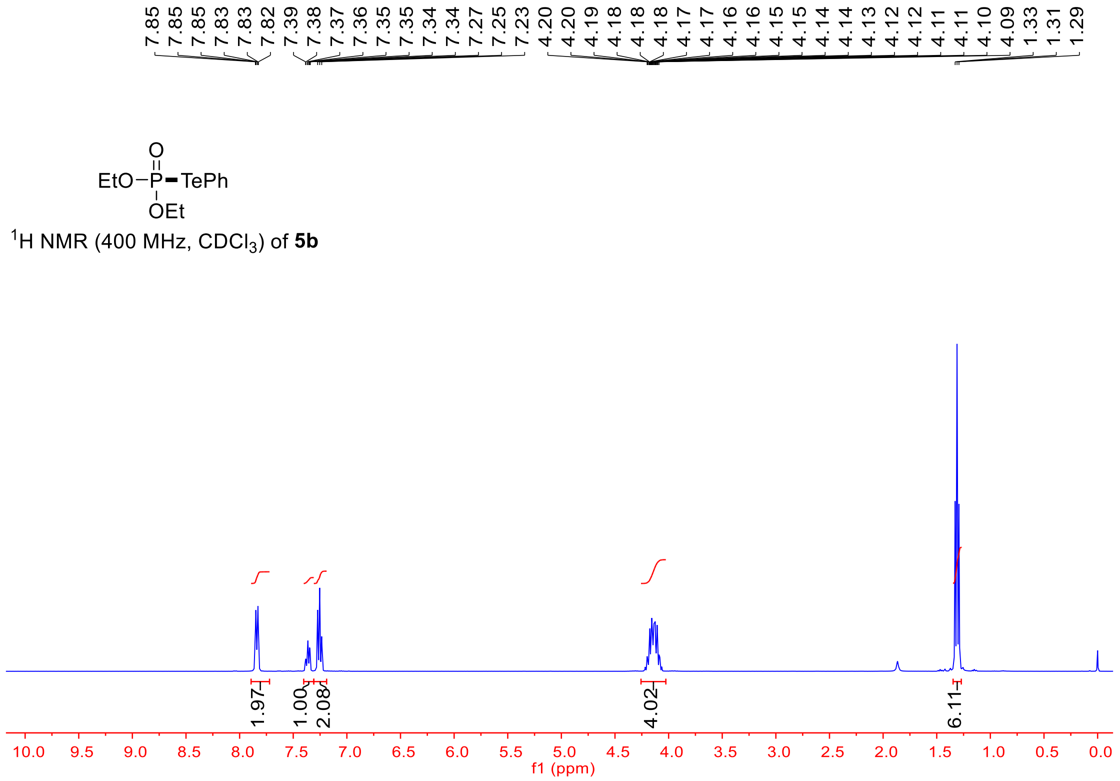

Figure S91: ^{1}H NMR spectrum for compound **5b**

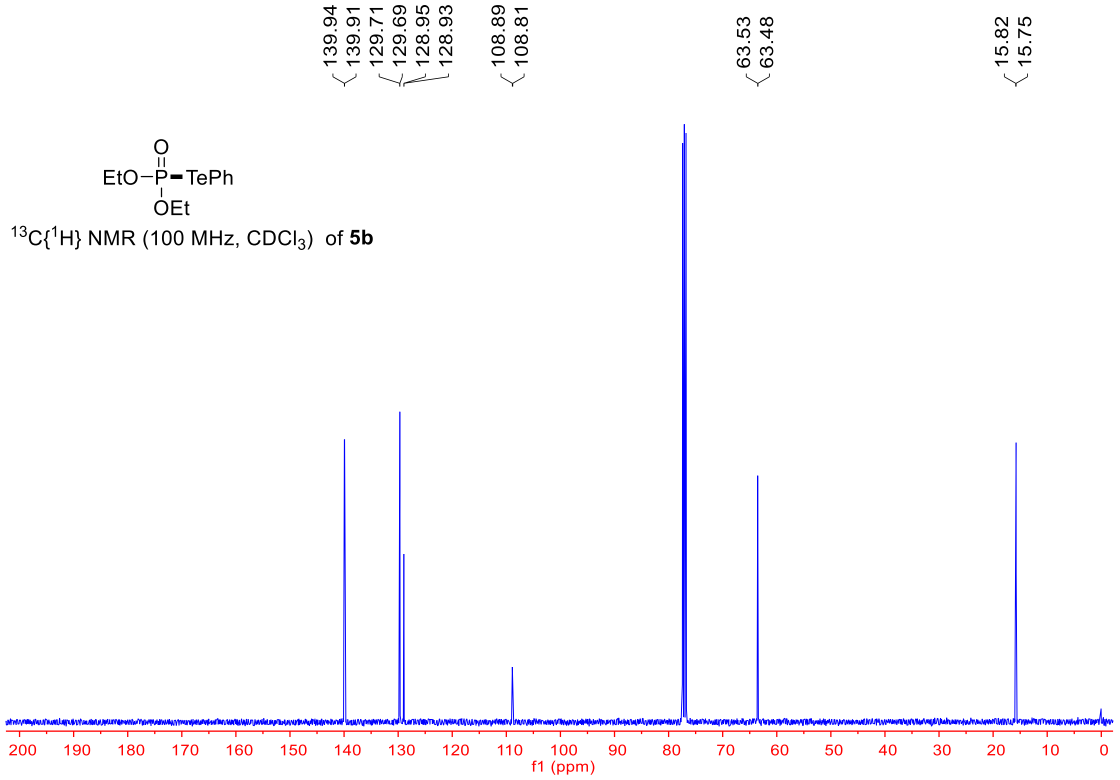

Figure S92: ^{13}C NMR spectrum for compound **5b**

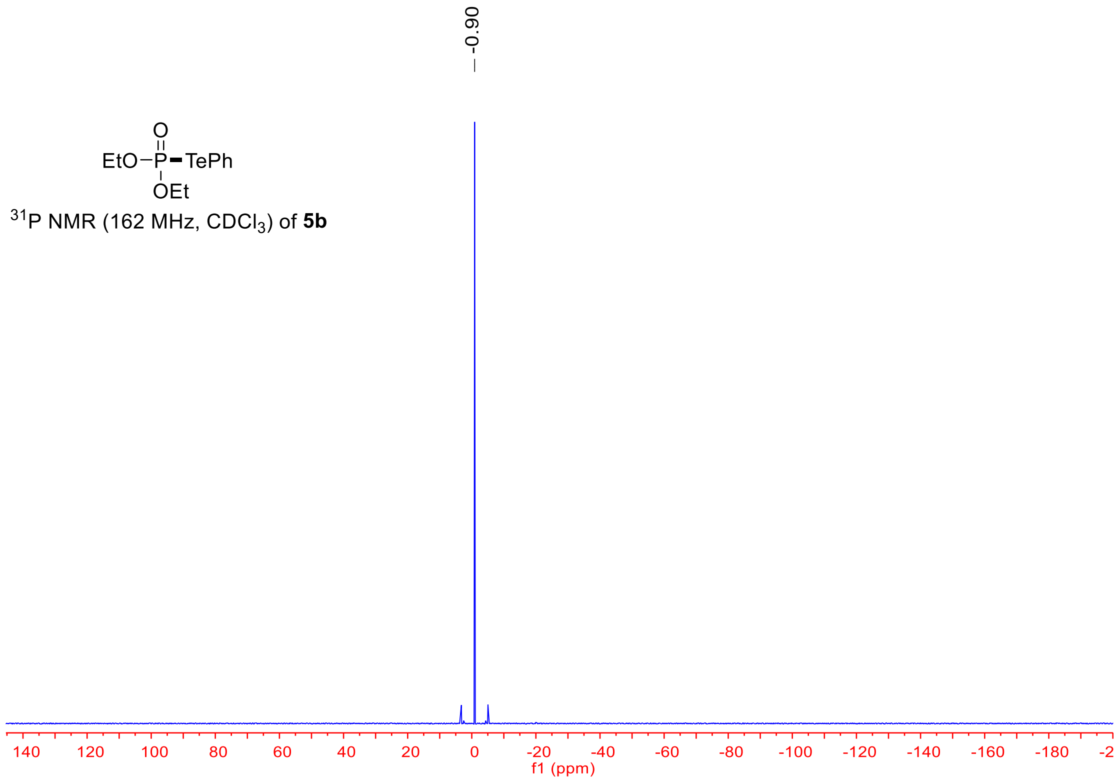

Figure S93: ^{31}P NMR spectrum for compound **5b**

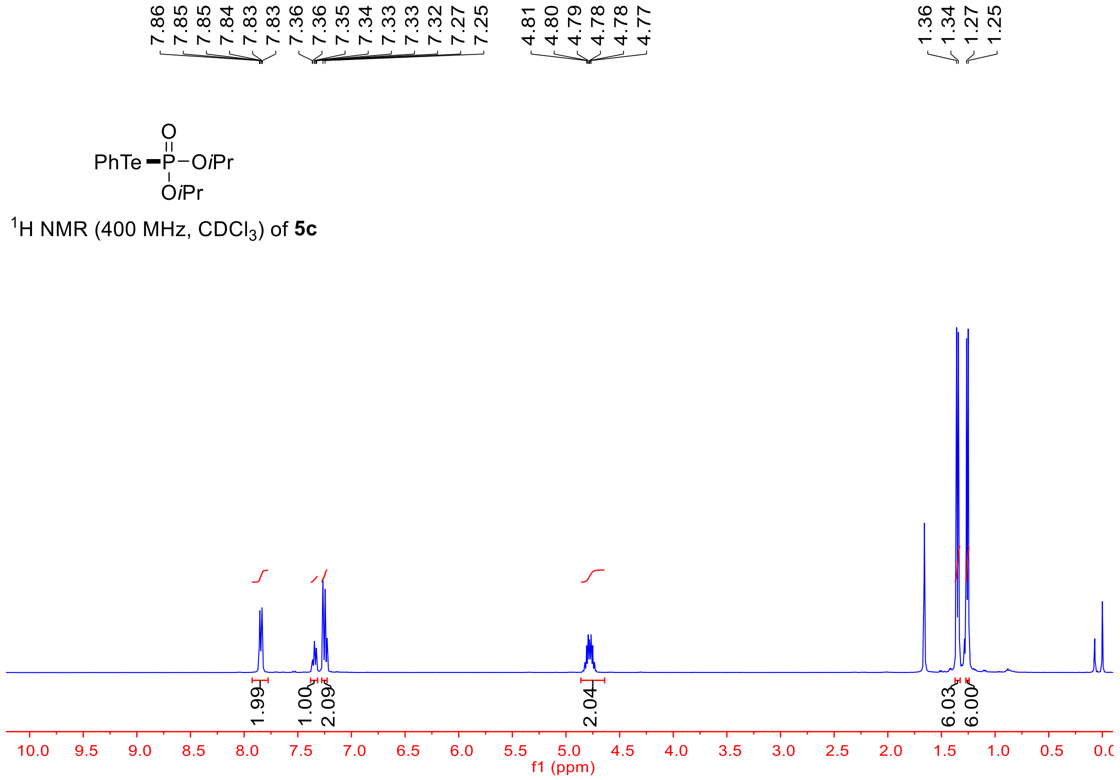

Figure S94: ^{1}H NMR spectrum for compound **5c**

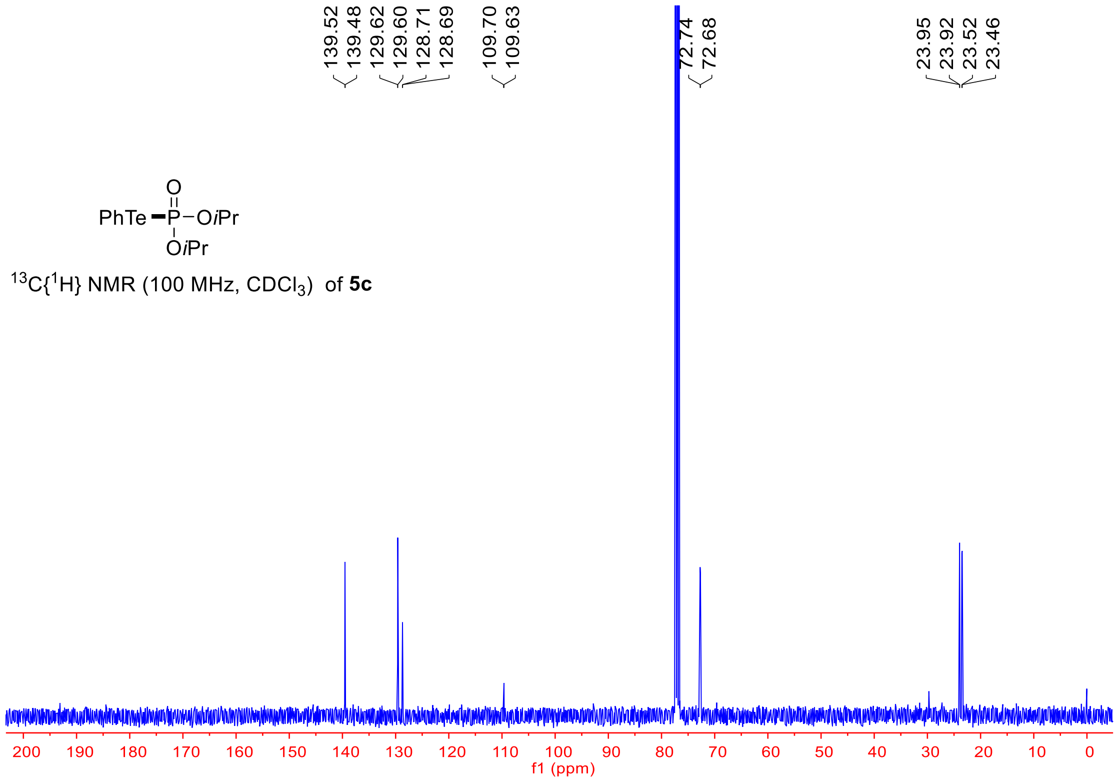

Figure S95: ^{13}C NMR spectrum for compound **5c**

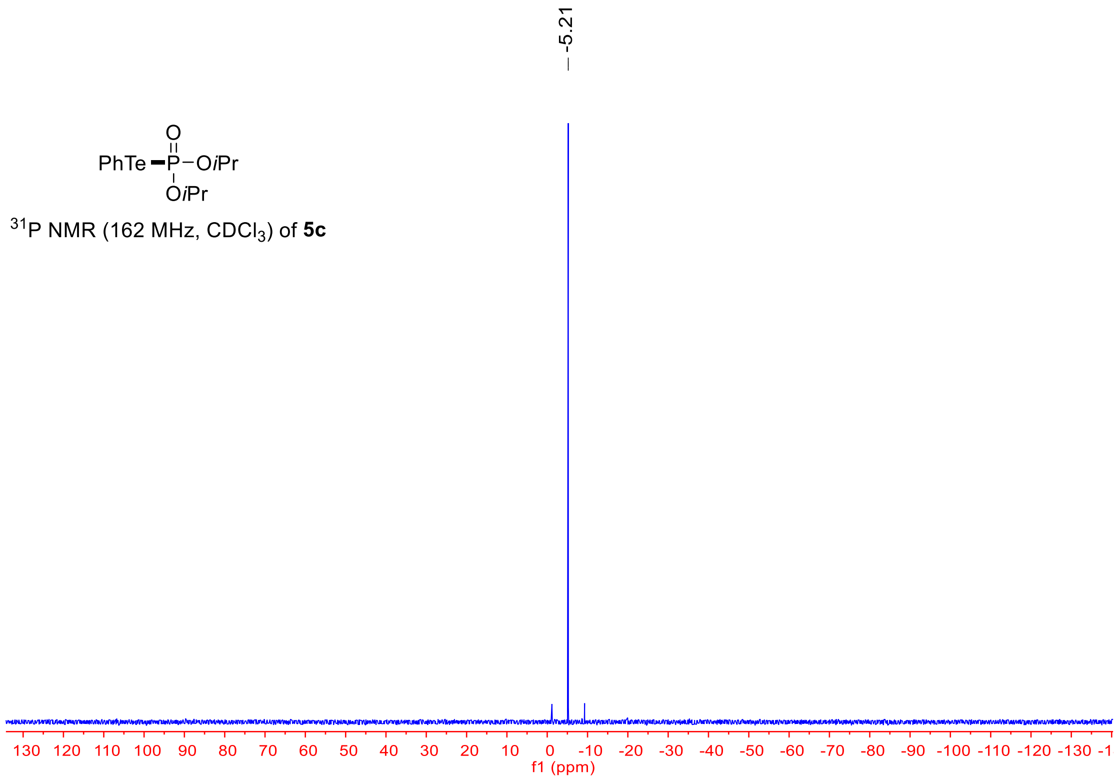

Figure S96: ^{31}P NMR spectrum for compound **5c**

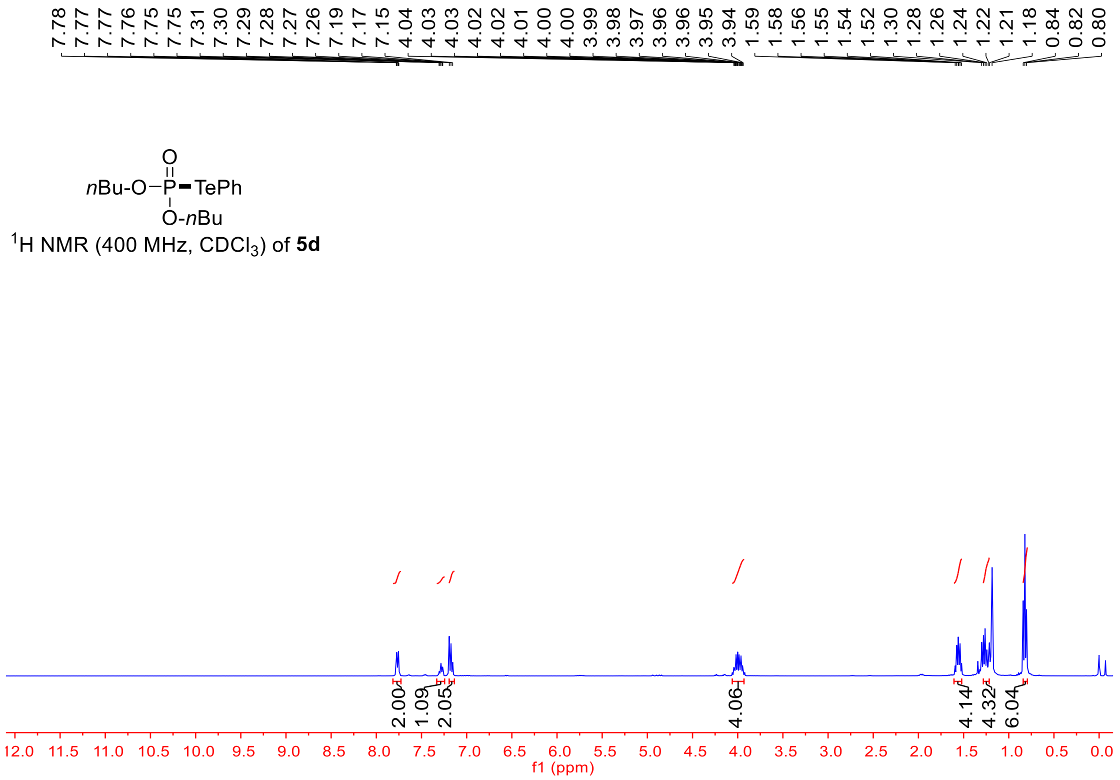

Figure S97: ^{1}H NMR spectrum for compound **5d**

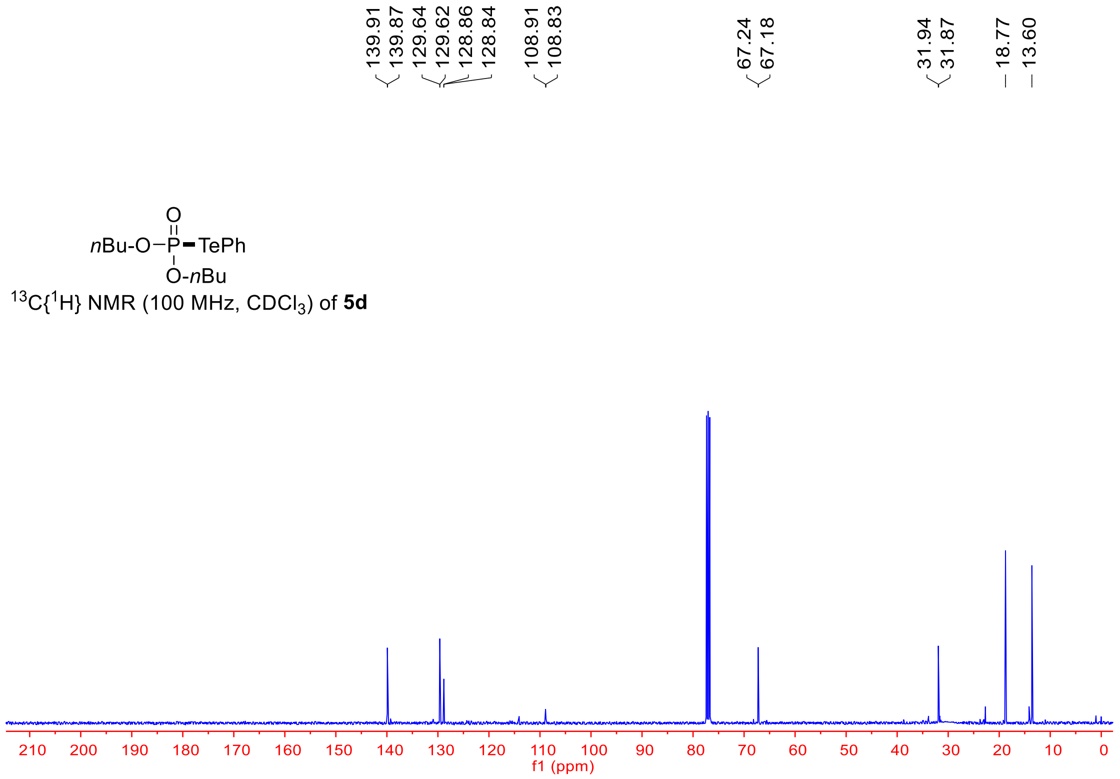

Figure S98: ^{13}C NMR spectrum for compound **5d**

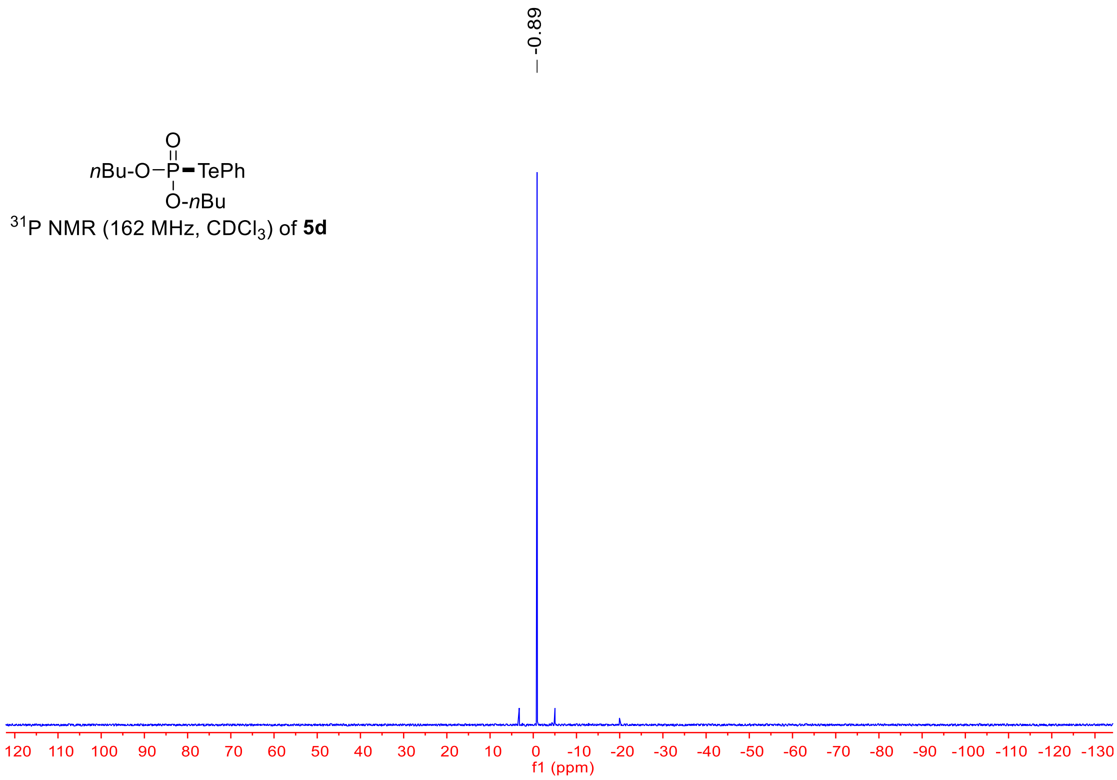

Figure S99: ^{31}P NMR spectrum for compound **5d**

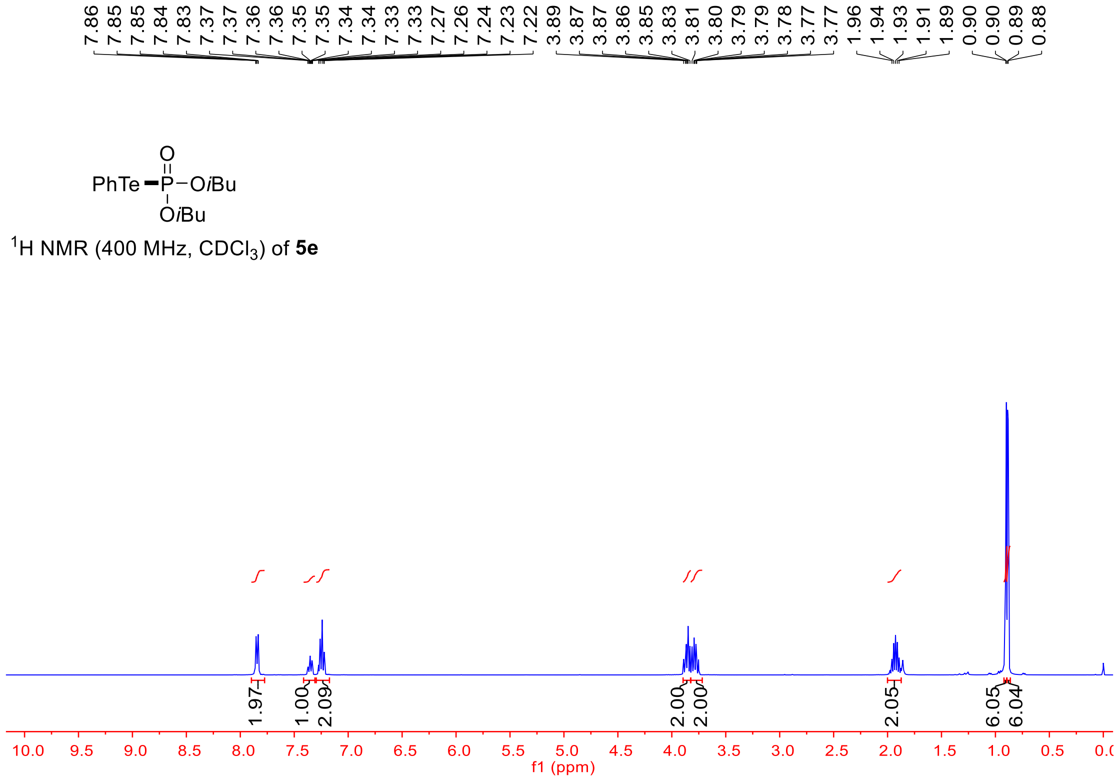

Figure S100: ^{1}H NMR spectrum for compound **5e**

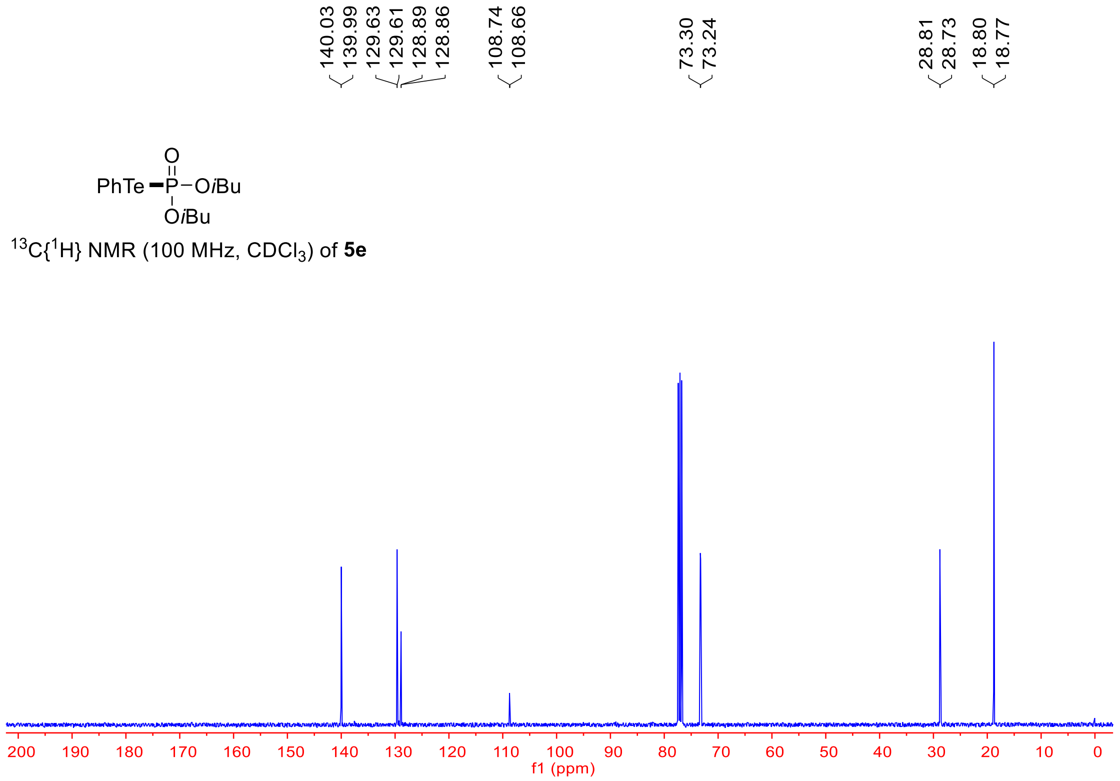

Figure S101: ^{13}C NMR spectrum for compound **5e**

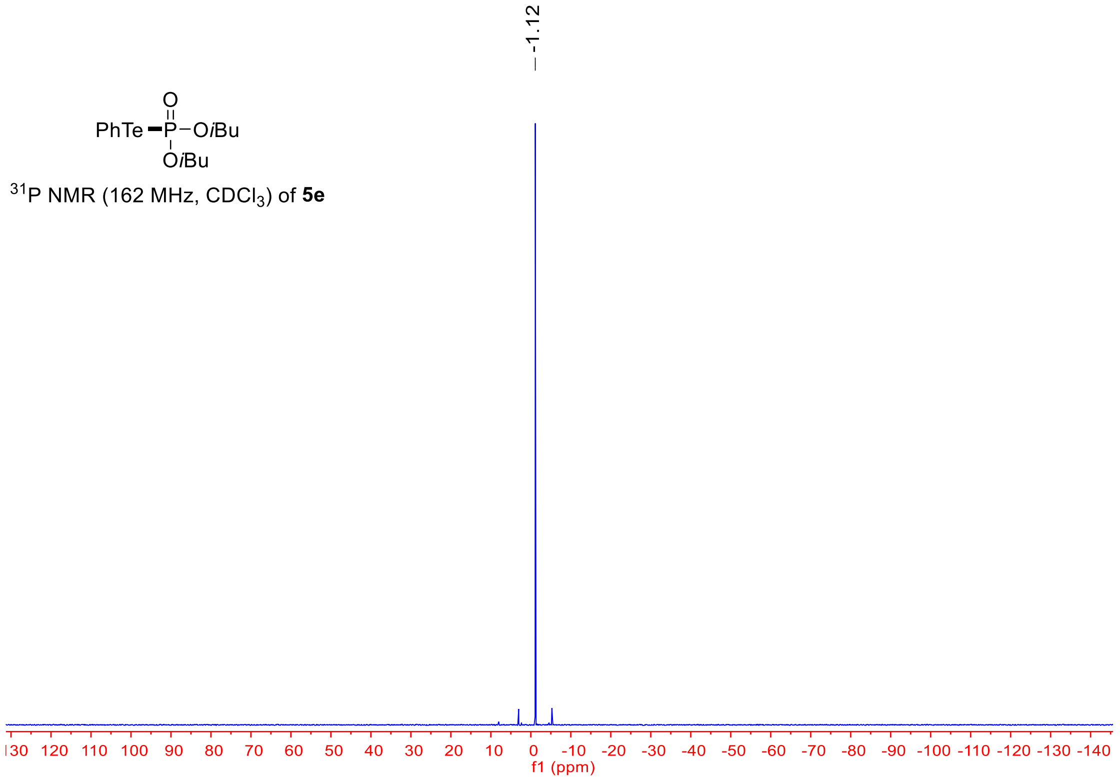

Figure S102: ^{31}P NMR spectrum for compound **5e**

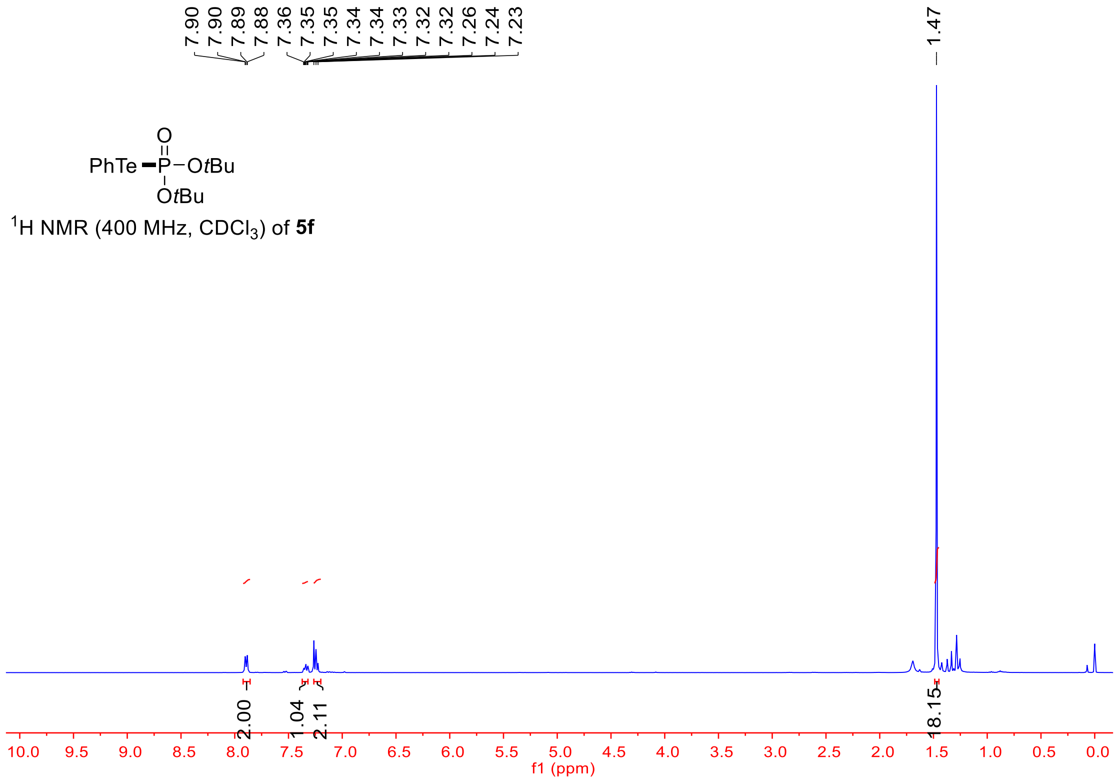

Figure S103: ^{1}H NMR spectrum for compound **5f**

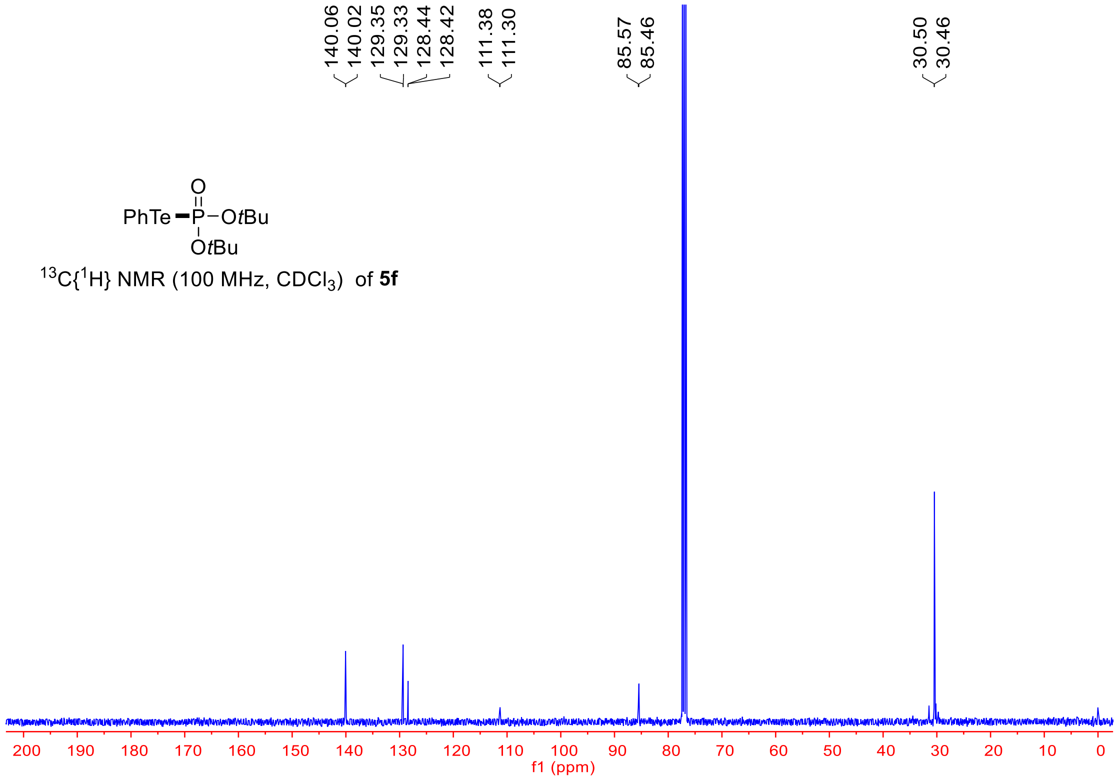

Figure S104: ^{13}C NMR spectrum for compound **5f**

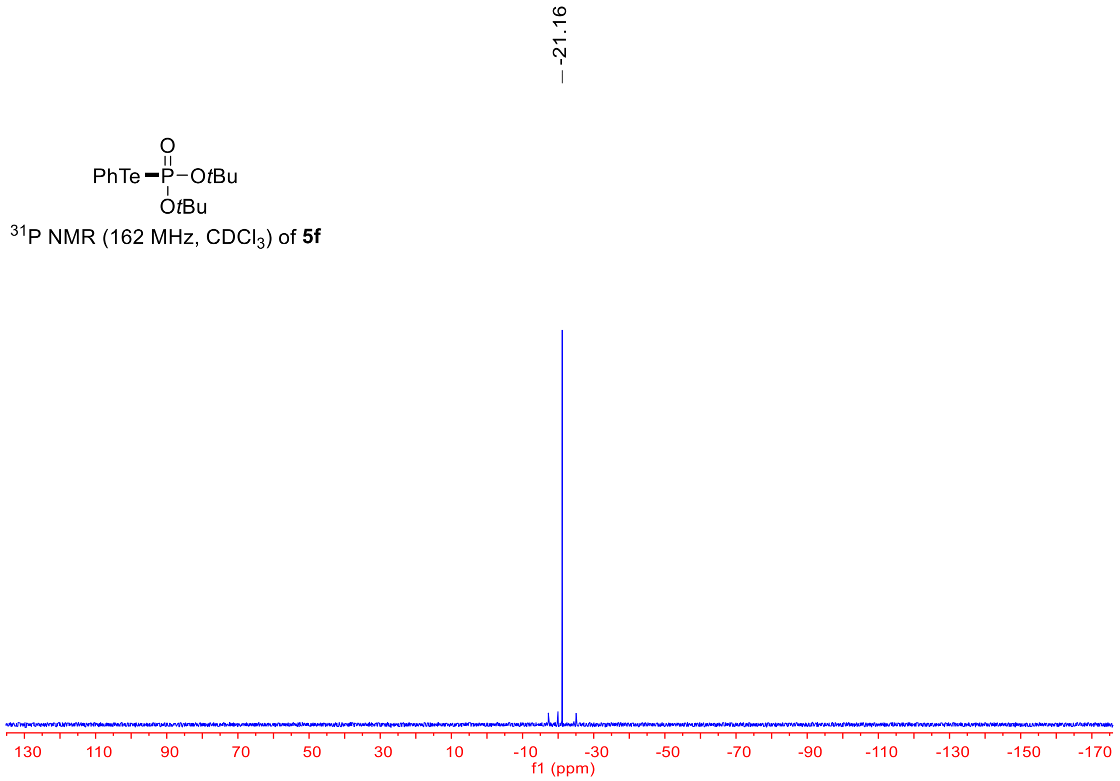

Figure S105: ^{31}P NMR spectrum for compound **5f**

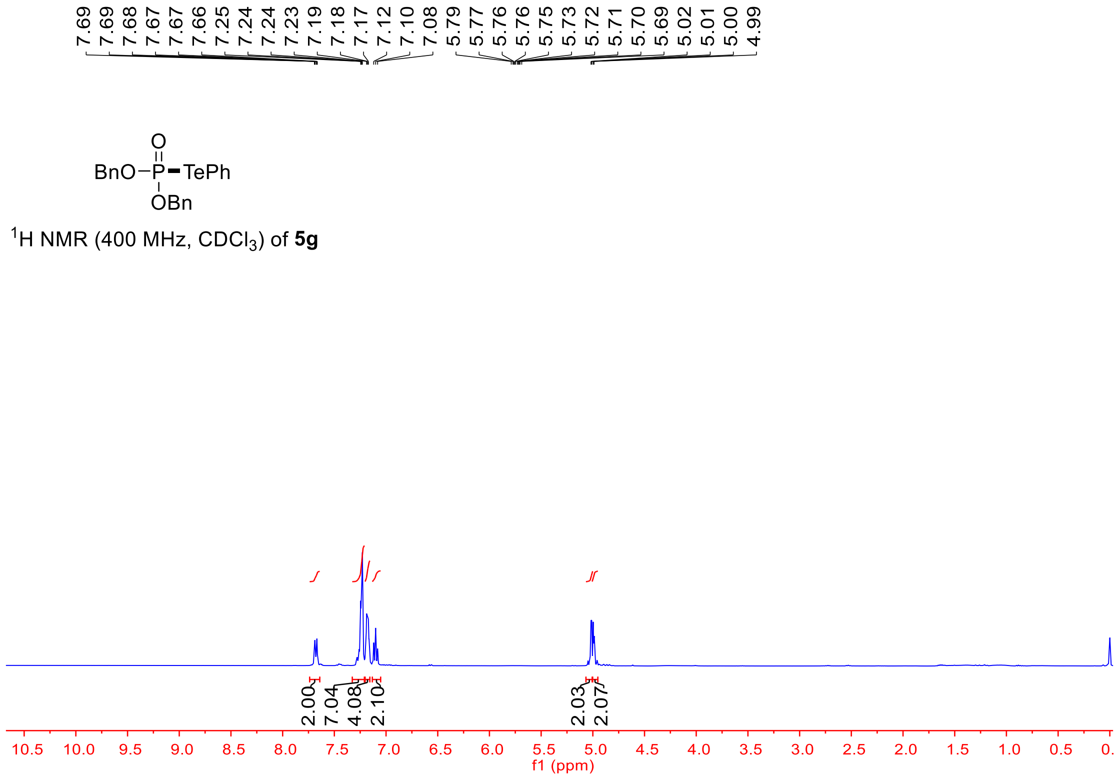

Figure S106: ^{1}H NMR spectrum for compound **5g**

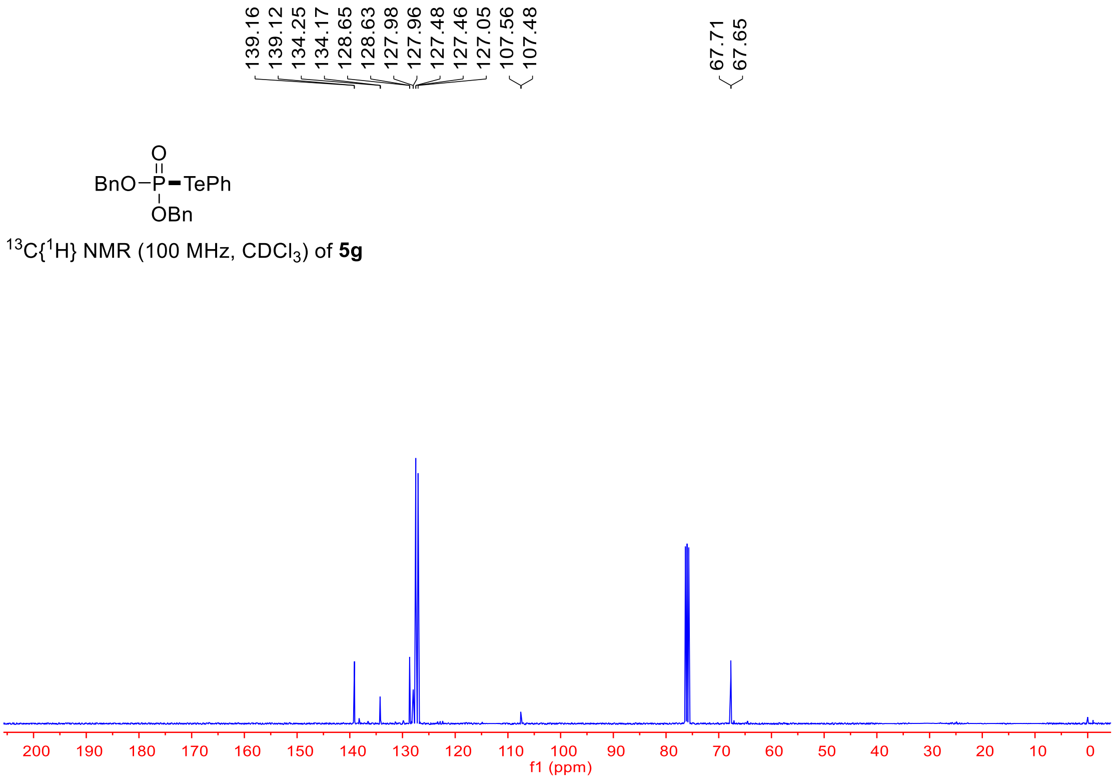

Figure S107: ^{13}C NMR spectrum for compound **5g**

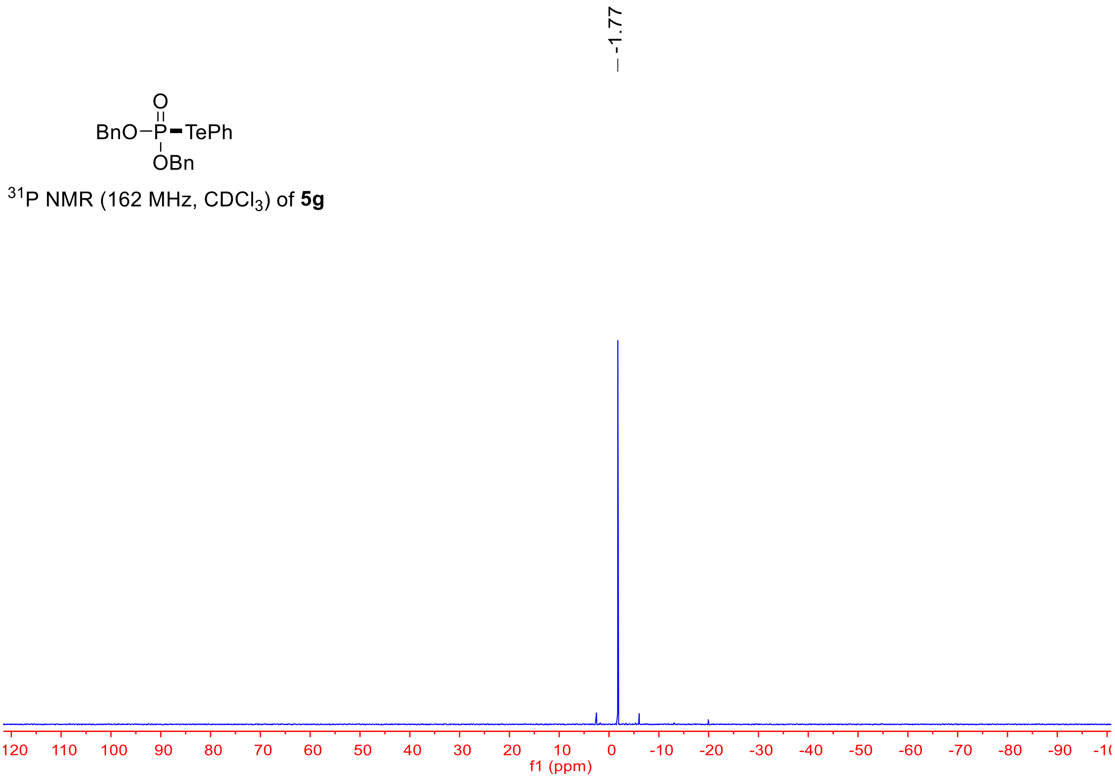

Figure S108: ^{31}P NMR spectrum for compound **5g**

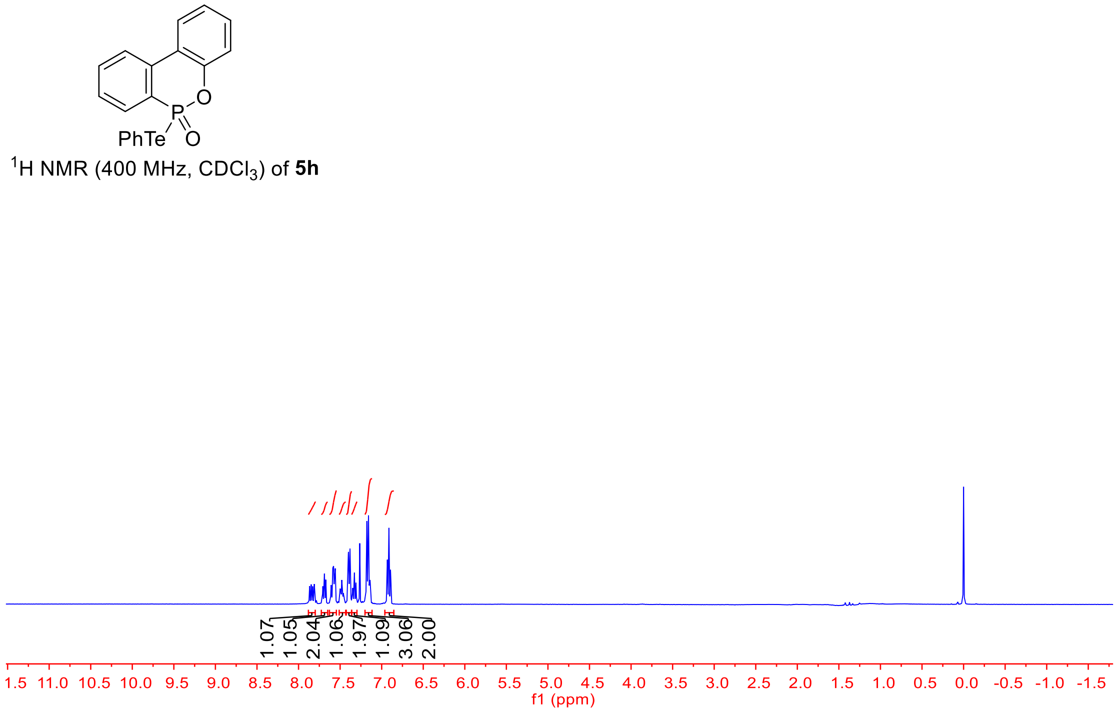

Figure S109: ^{1}H NMR spectrum for compound **5h**

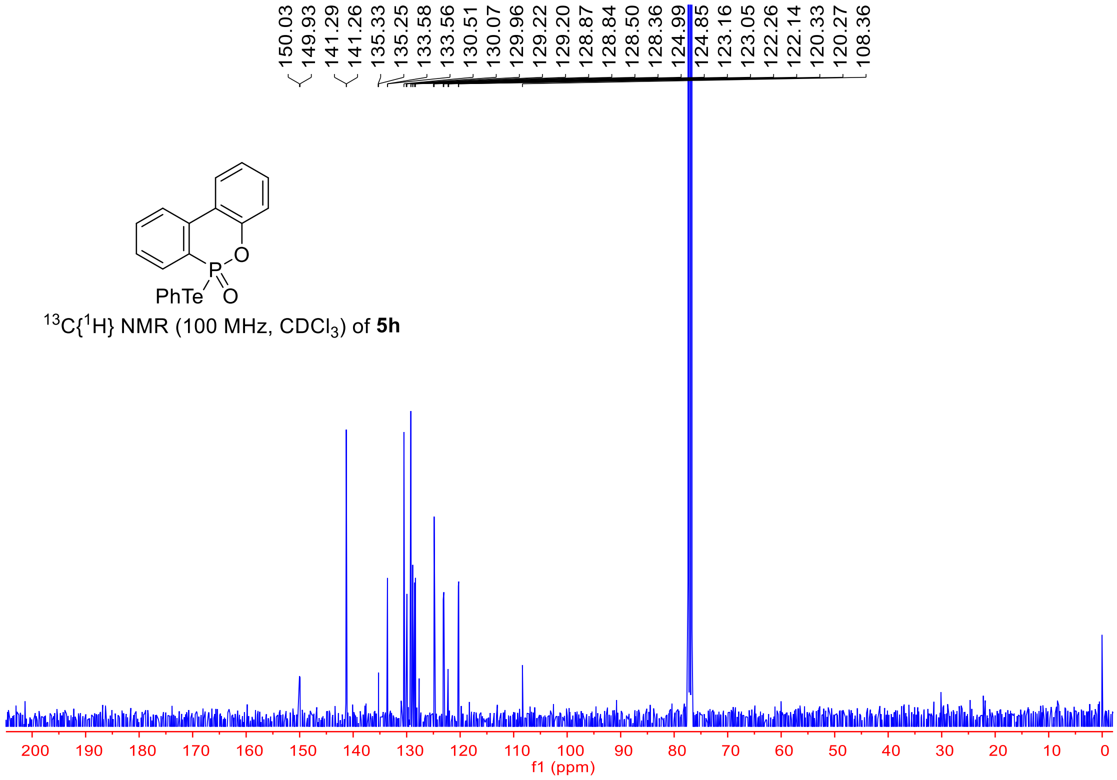

Figure S110: ^{13}C NMR spectrum for compound **5h**

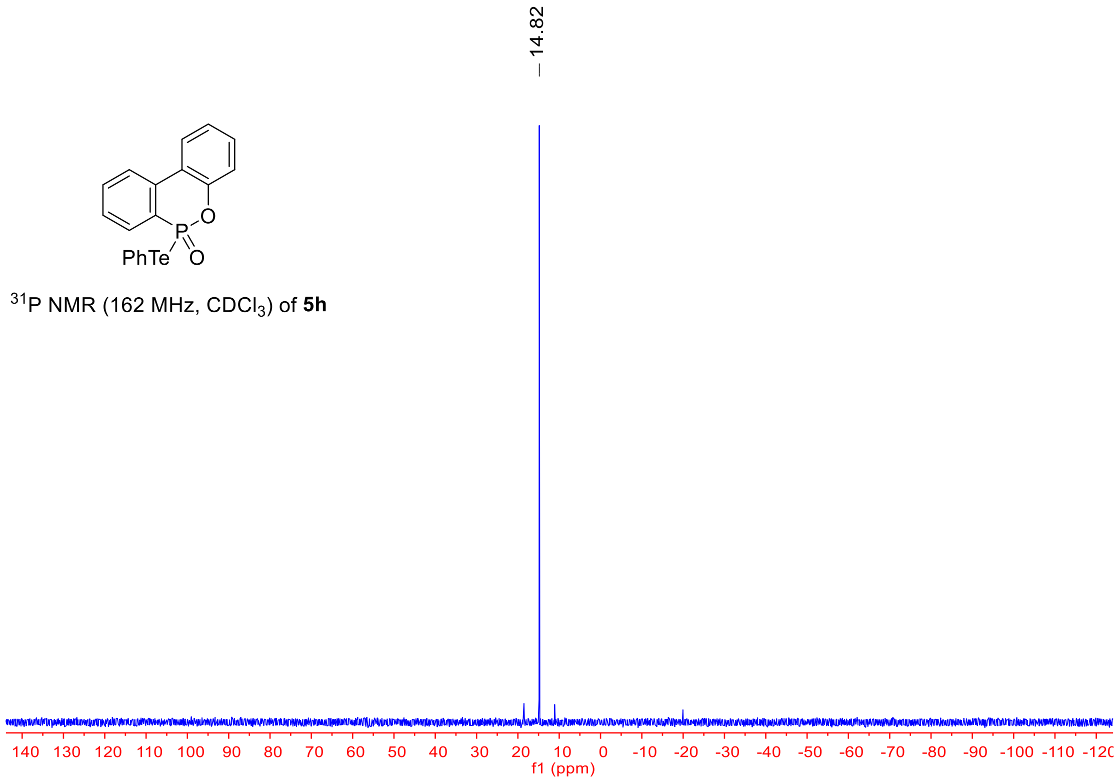

Figure S111: ^{31}P NMR spectrum for compound **5h**
